# A case for a binary black hole system revealed via quasi-periodic outflows

**DOI:** 10.1126/sciadv.adj8898

**Published:** 2024-03-27

**Authors:** Dheeraj R. Pasham, Francesco Tombesi, Petra Suková, Michal Zajaček, Suvendu Rakshit, Eric Coughlin, Peter Kosec, Vladimír Karas, Megan Masterson, Andrew Mummery, Thomas W.-S. Holoien, Muryel Guolo, Jason Hinkle, Bart Ripperda, Vojtěch Witzany, Ben Shappee, Erin Kara, Assaf Horesh, Sjoert van Velzen, Itai Sfaradi, David Kaplan, Noam Burger, Tara Murphy, Ronald Remillard, James F. Steiner, Thomas Wevers, Riccardo Arcodia, Johannes Buchner, Andrea Merloni, Adam Malyali, Andy Fabian, Michael Fausnaugh, Tansu Daylan, Diego Altamirano, Anna Payne, Elizabeth C. Ferraraa

**Affiliations:** ^1^ Kavli Institute for Astrophysics and Space Research, Massachusetts Institute of Technology, Cambridge, MA 02139, USA.; ^2^ Physics Department, Tor Vergata University of Rome, Via della Ricerca Scientifica 1, 00133 Rome, Italy.; ^3^ INAF Astronomical Observatory of Rome, Via Frascati 33, 00040 Monte Porzio Catone, Italy.; ^4^ INFN—Rome Tor Vergata, Via della Ricerca Scientifica 1, 00133 Rome, Italy.; ^5^ Department of Astronomy, University of Maryland, College Park, MD 20742, USA.; ^6^ NASA Goddard Space Flight Center, Code 662, Greenbelt, MD 20771, USA.; ^7^ Astronomical Institute of the Czech Academy of Sciences, Prague, Czech Republic.; ^8^ Department of Theoretical Physics and Astrophysics, Masaryk University, Brno, Czech Republic.; ^9^ Aryabhatta Research Institute of Observational Sciences (ARIES), Manora Peak, Nainital, 263002, India.; ^10^ Department of Physics, Syracuse University, Syracuse, NY 13244, USA.; ^11^ Astrophysics, Department of Physics, University of Oxford, Oxford, UK.; ^12^ The Observatories of the Carnegie Institution for Science, 813 Santa Barbara St., Pasadena, CA 91101, USA.; ^13^ Department of Physics and Astronomy, Johns Hopkins University, Baltimore, MD 21218, USA.; ^14^ Institute for Astronomy, University of Hawaii, Honolulu, HI 96822, USA.; ^15^ School of Natural Sciences, Institute for Advanced Study, 1 Einstein Drive, Princeton, NJ 08540, USA.; ^16^ NASA Hubble Fellowship Program, Einstein Fellow, Space Telescope Science Institute, 3700 San Martin Dr, Baltimore, MD 21218, USA.; ^17^ Center for Computational Astrophysics, Flatiron Institute, 162 5th Avenue, New York, NY 10010, USA.; ^18^ Charles University, Prague, Czech Republic.; ^19^ Racah Institute of Physics, The Hebrew University of Jerusalem, Jerusalem, Israel.; ^20^ Leiden Observatory, Leiden University, Leiden, Netherlands.; ^21^ University of Wisconsin Milwaukee, Milwaukee, WI 53211, USA.; ^22^ Department of Physics, Technion–Israel Institute of Technology, Haifa, Israel.; ^23^ Sydney Institute for Astronomy, School of Physics, The University of Sydney, New South Wales 2006, Australia.; ^24^ ARC Centre of Excellence for Gravitational Wave Discovery (OzGrav), Hawthorn, Victoria, Australia.; ^25^ Center for Astrophysics | Harvard & Smithsonian, 60 Garden St, Cambridge, MA 02138, USA.; ^26^ European Southern Observatory, Santiago, Chile.; ^27^ Max-Planck Institute for Extraterrestrial Physics, Garching, Germany.; ^28^ University of Cambridge, Cambridge, UK.; ^29^ Department of Physics and McDonnell Center for the Space Sciences, Washington University, St. Louis, MO 63130, USA.; ^30^ University of Southampton, Southampton, UK.; ^31^ Center for Research and Exploration in Space Science & Technology II (CRESST II), NASA/GSFC, Greenbelt, MD 20771, USA.

## Abstract

Binaries containing a compact object orbiting a supermassive black hole are thought to be precursors of gravitational wave events, but their identification has been extremely challenging. Here, we report quasi-periodic variability in x-ray absorption, which we interpret as quasi-periodic outflows (QPOuts) from a previously low-luminosity active galactic nucleus after an outburst, likely caused by a stellar tidal disruption. We rule out several models based on observed properties and instead show using general relativistic magnetohydrodynamic simulations that QPOuts, separated by roughly 8.3 days, can be explained with an intermediate-mass black hole secondary on a mildly eccentric orbit at a mean distance of about 100 gravitational radii from the primary. Our work suggests that QPOuts could be a new way to identify intermediate/extreme-mass ratio binary candidates.

## Teaser

Quasi-periodic outflows from the center of a galaxy reveal a potential supermassive–intermediate-mass black hole binary.

## INTRODUCTION

**Fig. 1.**
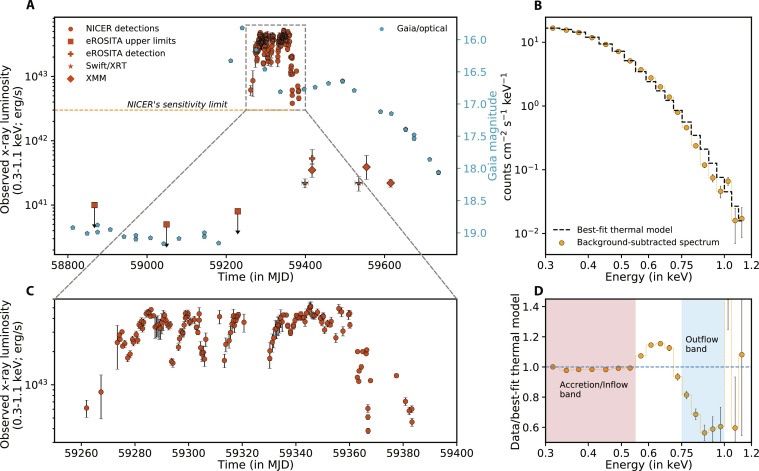
ASASSN-20qc’s long-term evolution and a sample x-ray spectrum highlighting the outflow. (**A**) ASASSN-20qc’s observed x-ray and optical evolution. Orange data represents x-ray (0.3 to 1.1 keV) data acquired by various instruments. The blue data show the Gaia magnitude. The horizontal (dashed) line represents NICER’s sensitivity limit of 3 × 10^42^ erg s^−1^ for a source at redshift, *z*, = 0*.*056. (**B**) Combined x-ray spectrum using all NICER data acquired over epochs of high absorption (yellow) and the best-fit emission model (black histogram). (**C**) Zoom-in of the outburst near the x-ray peak. (**D**) Ratio of the average energy spectrum using all NICER data acquired over epochs of minima in ODR and the best-fit thermal model. The outflow band is defined as the 0.75- to 1.00-keV band, while the inflow/accretion band is defined as the bandpass where the ratio is near 1, i.e., 0.30- to 0.55-keV band.

ASASSN-20qc (*1*) is an astrophysical flare that originated from the nucleus of a galaxy at a redshift of 0.056 (luminosity distance of 260 Mpcs). It was discovered by the All-Sky Automated Survey for SuperNovae [ASAS-SN; (*2*, *3*)] on 20 December 2020. Throughout the paper, we reference times with respect to this discovery date of modified Julian date (MJD) 59203.27. A follow-up optical spectrum revealed the presence of several hydrogen and oxygen emission lines, which facilitated the estimate of the redshift (*4*) (Materials and Methods, “Data and reduction” section) and a supermassive black hole (SMBH) mass of log $\left(M_{•}/M_{⊙}\right)=7.5_{-0.3}^{+0.7}$ (Materials and Methods, “Optical spectral modeling and black hole mass” and “ASASSN-20qc’s host galaxy properties and black hole mass” sections; table S2). A luminosity of 6 × 10^40^ erg s^−1^ from archival eROSITA data (Fig. 1A and Materials and Methods, “Data and reduction” section) indicates that before the outburst it was a low-luminosity active galactic nucleus (AGN; see Materials and Methods, “ASASSN-20qc’s location in the BPT and the WHAN diagrams suggests that it is an AGN” section) accreting at *<*0.002% of its Eddington limit.

Roughly 52 days after ASASSN-20qc’s optical discovery, the Neil Gehrels Swift observatory (Swift) observed it and detected x-rays. Following this detection, the Neutron star Interior Composition ExploreR (NICER) started a high-cadence (one to two visits per day) monitoring program (Fig. 1, A and C). We analyzed the NICER soft x-ray (0.3 to 1.1 keV) energy spectra in the early phases of the outburst and found that the spectrum was thermal (accretion disk dominated) and contained systematic residuals reminiscent of a broad absorption trough (Fig. 1D). We also obtained an XMM-Newton observation on 14 March 2021 (MJD 59287.34), roughly a month after the first NICER exposure, noting the presence of broad absorption residuals. Subsequent NICER spectra taken at various epochs of the outburst revealed that this absorption was variable throughout the outburst. A detailed photoionization modeling indicates that the dominant absorption feature is due to O VIII transitions in the 0.75- to 1.00-keV observed energy band blueshifted with a mildly relativistic velocity of about 30% of the speed of light. This evidence is indicative of an ultrafast outflow (UFO) (*5*). See fig. S4 and Materials and Methods, “X-ray energy spectral modeling: NICER and XMM-Newton detect an ultrafast outflow” section, for a detailed discussion on x-ray spectral modeling.

## RESULTS

**Fig. 2.**
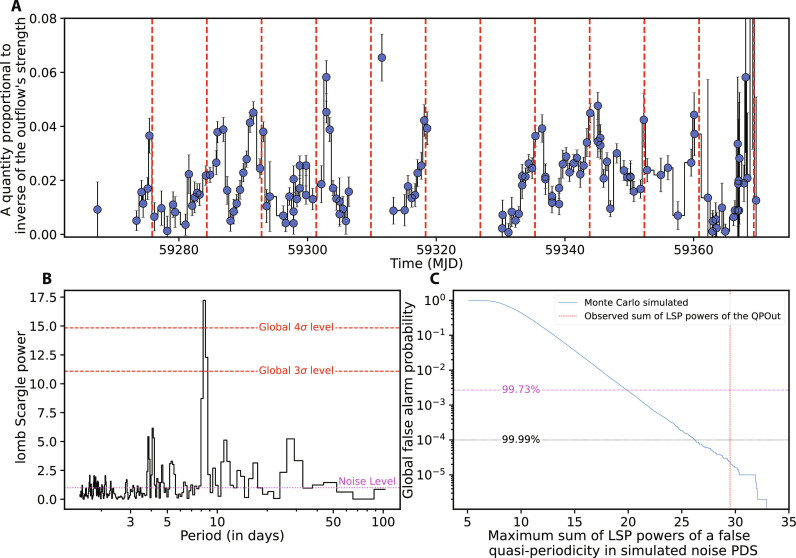
Summary of ASASSN-20qc’s timing analysis. (**A**) ASASSN-20qc’s ODR versus time. ODR is defined as the ratio of background-subtracted count rates in 0.75- to 1.00-keV (outflow) and 0.3- to 0.55-keV (continuum) bands. A lower ODR value implies a stronger outflow and vice versa. The dashed vertical red lines are uniformly separated by 8.5 days. (**B**) Lomb-Scargle periodogram (LSP) of the ODR. The strongest signal is near 8.5 days. The horizontal dashed red lines show the 3 and 4σ global false alarm probabilities as per (*6*). The noise in the periodogram is consistent with white with a mean LSP power value of 1 (see Materials and Methods, “Values in the LSP are consistent with white noise” section). (**C**) Global (trials-accounted) false alarm probability. This curve was generated using extensive Monte Carlo simulations (see Materials and Methods, “ODR timing analysis” section). The global statistical significance of the 8.5-day quasi-periodicity is *>*4.2σ.

To probe the interplay between the variable outflow and the thermal continuum emission, we calculated the ratio of the observed, background-subtracted count rates in the energy band dominated by the outflow (0.75 to 1.00 keV) and the continuum emission (0.30 to 0.55 keV) (see shaded regions of Fig. 1D). This quantity, which we define as the outflow deficit ratio (ODR), quantifies the amplitude of the outflow variability with respect to the continuum, and it is shown in Fig. 2A. The ODR curve showed repeating variations with a ≈8.5-day quasi-periodicity, which are not present in the unabsorbed continuum emission (figs. S5 and S6).

To quantify the variability and search for quasi-periodic signals in Fig. 2A, we computed the Lomb-Scargle periodogram [LSP; (*6*, *7*)] of the ODR curve (Fig. 2B). The highest power in the LSP is at 8*.*3 ± 0*.*3 days in multiple neighboring bins and is consistent with the time series in Fig. 2A. To estimate the false alarm probability that takes into account multiple bins, i.e., the chance probability of generating a signal as strong as the one observed from noise, we devised a detailed Monte Carlo method (see fig. S9 and Materials and Methods, “ODR timing analysis” and “Values in the LSP are consistent with white noise” sections). The global false alarm probability of the observed ≈8.5 days quasi-periodicity is *<*2 × 10^−5^ (*>*4.2σ; see Fig. 2C). Global refers to a blind search for signal over all the frequencies sampled, i.e., ∼a day to 100 days (see Fig. 2B).

To further probe the nature of this quasi-periodicity, we extracted and fitted time-resolved NICER x-ray spectra from individual maxima and minima in the ODR curve (see Materials and Methods, “Extracting composite spectra from NICER data” and “NICER time-resolved energy spectral analysis shows the same strong-weak outflow oscillatory pattern” sections, table S5, and fig. S10). Notably, the outflow has an order of magnitude higher column density (*N_H_*) during the minima phases of the ODR curve with respect to the maxima: median value of (12*.*6 ± 5*.*5) × 10^21^ cm^−2^ and (1*.*8 ± 0*.*7) × 10^21^ cm^−2^ for the minima and the maxima, respectively. The ionization parameter, defined as logξ = *L/nr*^2^, in units of erg s^−1^ cm, where *L* is the ionizing luminosity between 1 and 1000 Ryd (1 Ryd = 13.6 eV), *n* is the number density of the material, and *r* is the distance of the gas from the central source, is on average only slightly higher during the minima than during the maxima. Instead, the outflow bulk velocity is stable at around 0.35*c*, where *c* is the speed of light (see fig. S10).

On the basis of the above timing analysis and the time-resolved spectral modeling, we conclude that ASASSN-20qc exhibits quasi-periodic outflows (QPOuts) about once every 8.5 days (precisely 8.3 ± 0.3 days). By the term QPOuts, we denote quasi-periodic variations of the outflowing material (see Fig. 2).

## DISCUSSION

We considered several theoretical models to interpret the above observations including a precessing inner accretion disk, clumpy or slow outflow, x-ray reflection, accretion disk instabilities, quasi-periodic eruptions, and repeating partial tidal disruption event (TDE), but disfavor them based on several independent lines of arguments. See Materials and Methods, “Data and reduction,” “X-ray energy spectral modeling: NICER and XMM-Newton,” “A single clumpy outflow is disfavored,” and “The outflow is present even at 200 times lower x-ray luminosity” sections for more details and Table 1 for a summary of the strengths and weaknesses of these various models.

**Table 1.** A summary of the strengths and the weaknesses of various models considered in this work to explain ASASSN-20qc’s observed x-ray spectro-timing variability. See Materials and Methods, “X-ray energy spectral modeling: NICER and XMM-Newton detect an ultrafast outflow,” “A single clumpy outflow is disfavored,” and “The outflow is present even at 200 times lower x-ray luminosity” sections, and the Supplementary Materials for more details.

Model/class of models	Strengths	Weaknesses	Notes
Inner disk precession	Thought to be commonly seen in stellar-mass black hole binaries (*105*)	The lack of strong continuum modulation and the observed changes from high column, high ionization parameter to low column, low ionization along with constant outflow speed More importantly the lack of a strong quasi-periodicity in the 0.3–0.55 keV continuum variations are inconsistent with precession with all known types of outflows (*118*–*122*)	Disfavored based on physical reasoning
Clumpy outflow	–	The outflow geometry would need to be fine-tuned to have uniformly separated clumps. The probability of formation of such clumps by chance is less than 1 in 50,000	Disfavored due to low likelihood
Slow outflow	Slow outflow can, in principle, produce similar spectral signatures	The XMM-Newton/RGS and EPIC/pn spectrum rule out a slow outflow that can produce such a broad feature A typical slow outflow is distant from the SMBH and cannot produce a rapid (∼week timescale) quasi-periodic variability seen here	Disfavored based on physical reasoning (see Materials and Methods, “The broad absorption residuals cannot be explained with slow outflows” section)
X-ray reflection by a corona	Seen in several highly accreting AGN with an x-ray corona (*119*)	Lack of a comptonizing corona/power-law component in the x-ray spectrum	Disfavored based on lack of evidence in data (see Materials and Methods, “X-ray energy spectral modeling: NICER and XMM-Newton detect an ultrafast outflow” section)
X-ray reflection by a disk	Argued to operate at least in one changing-look AGN (*95*)	Lack of a geometrically thick surface for reflection, would require a fine-tuned disk geometry Unphysically large fraction of reflected emission compared to the primary thermal emission	Disfavored based on statistical argument and physical grounds (see Materials and Methods, “X-ray energy spectral modeling: NICER and XMM-Newton detect an ultrafast outflow” section, for more discussion)
Magnetically arrested accretion disk	Preliminary work by (*120*) suggests that outflows can be produced through repeated magnetic reconnection events	Based on state-of-the-art high-resolution simulations, it is unclear if such outflows would be quasi-periodic in nature Such regular outflows are not seen in lower-resolution simulations Lack of strong quasi-periodicity in the continuum variations	Viable but no clear indication in the state-of-the-art simulations [but see figure 8 of (*120*)]
Quasi-periodic eruptions (QPEs)	Seen in a small sample of AGN	QPEs manifest as large amplitude flux bursts as opposed to changes in ODR Variable outflows have not been reported in known QPE sources.	Disfavored because the observed signal is distinct (see Materials and Methods, “A spectral model with two thermal components akin to quasi-periodic eruptions is ruled out” section)
Repeating partial stellar tidal disruption	Argued to operate in at least systems (*53*, *121*, *122*)	The expected orbital period would be orders of magnitude longer than what is seen here (*103*) No evidence for a similar variability in the optical light curve A stellar core’s influence radius would be too small to produce the observed outflow	Disfavored based on physical reasoning
Stellar debris stream	Could provide obscuration when highly inclined	Stellar debris would be tidally spread along the whole orbit, turning off the periodicity; the material would need to be continuously replenished (see partial TDE above)	Disfavored based on physical reasoning
Radiation pressure driven outflows	Observed in a sample of accreting stellar-mass black holes (*123*)	The persistence of the outflow over a factor of >200 change in x-ray flux suggests negligible radiation driving Fine-tuning of the disk properties for obtaining short-enough instability period (*124*) No evidence for a similar variability in the soft x-ray continuum	Disfavored based on the need for fine-tuning
A scaled-up version of quasi-periodic oscillations	Occurring in stellar-mass black-hole binaries [e.g., (*125*)]	The lack of a strong quasi-periodicity in the thermal continuum (0.3–0.55 keV band)	Disfavored due to lack of a precedent
An orbiting object repeatedly perturbing the SMBH accretion disk	Can explain QPOuts Ultrafast outflow production supported by GRMHD simulations Consistent with TDE statistics and production rates of SMBH-IMBH binaries (*126*)	An IMBH distance of ∼100 *r*_g_ makes full 3D GRMHD simulations computationally expensive For 2D simulations, magnetorotational instability enabling accretion tends to stop operating after sometime (within a few ×10,000 *M* which corresponds to ∼100days), which makes comparison with data limited at later epochs	Viable but no precedent (see sections S2 and S3; see section S4.4 for caveats)

**Fig. 3.**
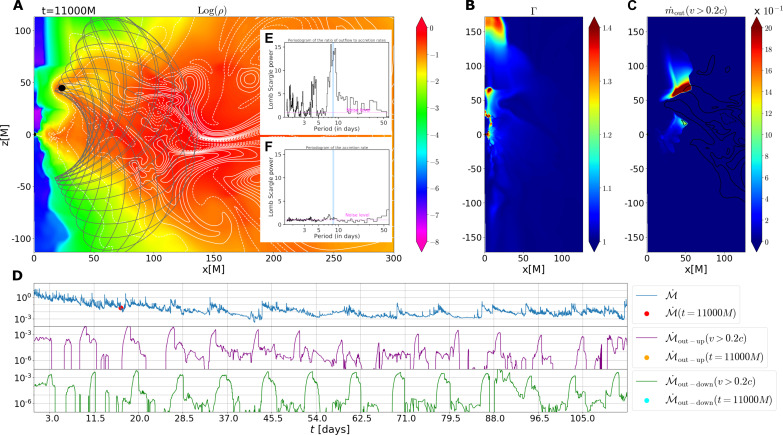
A sample snapshot from our GRMHD simulation (2D HARM, run 14 from table S7). For this case, the SMBH mass was set to 10^7.^4^^*M*_⊙_ and the perturbing companion is in an elliptical orbit (eccentricity, *e* = 0.5) with an observed orbital period of 8.5 days and has an influence radius of three gravitational radii [1*M* = *GM*_•_/*c*^2^ = 0.25(*M*_•_/10^7.4^*M*_⊙_)*AU*]. (**A**) Spatial distribution of the logarithm of mass density expressed in arbitrary units. The horizontal and the vertical axes are spatial coordinates expressed in gravitational radii (units of *M*). The white contours indicate the magnetic field configuration. The position and size of the perturber are shown by the black circle, while the gray line displays its trajectory in the 2D slice. (**B**) Spatial distribution of the Lorentz factor of the gas bulk motion. (**C**) Spatial distribution of the mass outflow rate with *v >* 0*.*2*c*. The outflow rate is color-coded using arbitrary units according to the color bar to the right. (**D**) Temporal profiles of the inflow rate (blue), the outflow rate through the upper funnel (purple), and the outflow rate through the lower funnel (green). The inflow and outflow rates are expressed in arbitrary units. The time is expressed in days in the observed frame. The colored points/dots indicate the time of the snapshot. Vertical lines are uniformly separated by 8.5 days. (**E** and **F**) LSP of the ratio of the outflow to the inflow rates (E) and the accretion rate (F) from run 14 sampled exactly as the real data. The peak signal in (E) is broad with a value of $8.5_{-1.1}^{+0.7}$ days and is consistent with the observed value of 8.3 ± 0.3 days (shaded blue band), while no such signal is present in the accretion rate periodogram (F), i.e., an elliptical binary can reproduce the observed quasi-periodicity in the outflow strength without similar variations in the continuum.

Instead, we propose a viable model with an orbiting, inclined perturber that repeatedly crosses the inner accretion flow. This scenario can explain the presence of QPOuts if the perturber is characterized by a sufficiently large influence radius at a given distance (*8*, *9*). To further verify this model, we performed extensive two-dimensional (2D) general-relativistic magnetohydrodynamic (GRMHD) simulations of an object in orbit around an SMBH using the HARMPI code (*10*, *11*) based on the original HARM code (*12*, *13*) (see Supplementary Materials, “Perturber-induced outflow scenario” section for details). Regardless of the specific setup, QPOuts are triggered by the passing perturber once per its orbit (see Fig. 3 and table S7 for an overview). The simulations predict a persistent magnetized outflow from the inner flow with a roughly constant radial velocity profile, which is mass-loaded periodically when the secondary crosses the primary disk. This is consistent with the observation of a persistent outflow in the maxima, which is boosted during the minima of the ODR. For all the cases, the perturber is highly inclined with respect to the equatorial plane of the accretion flow, which leads to the recurrent, periodic, mildly relativistic outflow regardless of the background accretion-flow state. An ordered and stable poloidal magnetic field in the funnel region accelerates the ejected matter to mildly relativistic velocities. Furthermore, a mildly eccentric orbit with an eccentricity of 0*.*5 to 0*.*7 can naturally induce departures from strict periodicity, which is evident from the LSP peak full width at half maximum (FWHM) of ∼1 day as well as from the outflow-rate temporal profiles in the bottom panels in Fig. 3. One caveat of the 2D GRMHD simulations is that while magnetorotational instability (MRI)—which is responsible for accretion onto the SMBH—is active at the distance of the perturber, it decays after a few ×10,000 *M* (or ∼100 days) in the inner regions of the accretion flow (∼a few gravitational radii). Thus, making direct comparisons of simulations to data beyond 100 days becomes challenging. However, since the observed QPOuts span about 100 days, our simulations with active MRI were performed on similar timescales and they show that such a scenario provides a potential mechanism for producing QPOuts. Further work using 3D GRMHD simulations where MRI does not decay with time are needed to track such systems for extended periods (see section S4.4 for more discussion).

The observed ratio of the outflow to the inflow rate of about 20% during the ODR minima is consistent with a perturber influence radius of *R* ∼ 3 gravitational radii when compared to the analogous ratio derived from GRMHD simulations (see section S4 for details). Independent of the GRMHD simulations, simple analytical reasoning yields a similar estimate (see the second paragraph of section S4). Taking into account that the ejected outflow clumps originate in the underlying flow, which can be treated as an advection-dominated accretion flow [ADAF: (*8*, *14*)], and their sizes are comparable to *R*, such a length scale would be in agreement with the inferred column density of 10^22^ cm^−2^ of the spectroscopically detected UFO. Considering the tidal (Hill) length scale of a massive perturber as well as the radius, within which the surrounding gas comoves with the perturber, we arrive at a rather broad range of the perturber masses ∼10^2^ − 10^5^*M*_⊙_. This broad range already includes the uncertainty in the primary SMBH mass (see section S4 for further discussion and figs. S13 and S15).

**Fig. 4.**
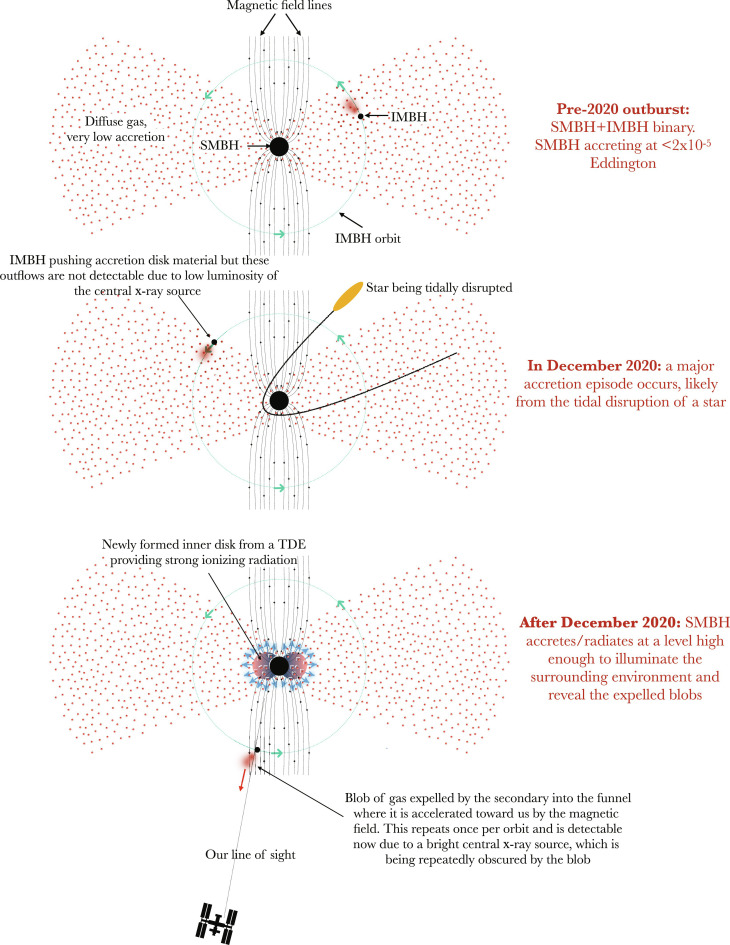
Schematic of a potential model for ASASSN-20qc. A gravitationally bound (preexisting) IMBH located at roughly 100 R*_g_* from the central SMBH can explain the repeated outflows seen here. The overall outburst could have been triggered by a tidal disruption of a passing star by the SMBH, which creates a compact accretion disk that naturally enhances the x-ray emission and consequently illuminates the surrounding environment and the presence of the IMBH secondary. Secondary plunges through the preexisting (non-TDE) accretion flow, modulating the outflow on the orbital period. Relative sizes are not to scale.

Distinct from the QPOuts, the optical light curve shown in fig. S12 exhibits a smooth rise, peak, and decay on a timescale of ∼150 days. This timescale is broadly consistent with the canonical fallback time of the debris from a TDE [e.g., (*15*)] with a black hole mass of *M*_•_ ∼10^7^*M*_⊙_ and a solar-like star. The evolution of the optical/ultraviolet (UV) temperature and photosphere radius during the outburst is also very similar to those of known TDEs [compare figure S12 with figure 8 of (*16*) and figure 1 of (*17*)]. The time delay between the x-ray and the optical outbursts of a few months (see Fig. 1A) has also been seen in several TDEs [e.g., (*18*, *19*)]. Finally, the soft x-ray spectrum is also strikingly similar to thermal x-ray TDEs. Therefore, a reasonable interpretation is that the overall outburst in the optical, UV, and x-rays was induced by a TDE, which produces a bright inner accretion disk, i.e., a soft x-ray source, which is quasi-periodically obscured by the blobs driven by the orbiting perturber (see Fig. 4).

Attributing the outburst to a TDE, we can further constrain the mass of the secondary based on the argument that the gravitational wave inspiral time should be greater than the typical time for a stellar disruption in a galaxy. Using a TDE rate of 10^−4^ year^−1^ [e.g., see figure 10 in (*20*)] would require the SMBH-perturber system to have a merger timescale of ≳ 10^4^ years. This limits the perturber mass to the range of 10^2^ to 10^4^*M*_⊙_, i.e., to the intermediate-mass black hole (IMBH) range (see the bottom panel of fig. S15). For such mass and distance of the secondary, the gravitational radiation is weak and the period of the system will not evolve substantially in the next decade, making the signal lay outside the frequency range of the upcoming space-based gravitational wave observatory LISA. The unique combination of an SMBH-IMBH pair experiencing the TDE makes such observation rather rare, though not entirely implausible. Within the cosmological volume inside *z* ∼ 0*.*06, we estimate *N*_pair*,*TDE_ = 0*.*07 to 5*.*3 TDEs per year in hosts with tight SMBH-IMBH pairs (out of ∼2*.*5 million galaxies; see section S4.2 for further discussion on the estimated event rate and the detectability of the system in gravitational waves).

In summary, our work highlights the new astrophysical phenomenon of QPOuts and the importance of high-cadence optical and x-ray monitoring observations to potentially uncover electromagnetic signatures of tight binary black hole systems. The identification of such SMBH-IMBH binaries, i.e., intermediate/extreme mass ratio inspirals (I/EMRIs), has fundamental implications for multi-messenger astrophysics and for our understanding of black hole growth and evolution.

## MATERIALS AND METHODS

### Data and reduction

For this work, we acquired/used multiwavelength data in the x-ray, optical, UV, and radio bands. Data reduction for each of the telescopes/instruments is described below. Throughout this paper, we adopt a standard ΛCDM cosmology with *H*_0_ = 67.4 km s^−1^ Mpc^−1^, Ω*_m_* = 0.315, and Ω_Λ_ = 1 − Ω*_m_* = 0.685 (*21*). Using the cosmology calculator of (*22*), ASASSN-20qc’s redshift of 0.056 corresponds to a luminosity distance of 259.5 Mpcs.

#### X-ray

ASASSN-20qc’s x-ray data used in this work were acquired by six different instruments: NICER’s X-ray Timing Instrument [XTI; (*23*)], XMM-Newton’s European Photon Imaging Camera’s (EPIC) pn (*24*) and MOS (*25*) detectors, XMM-Newton’s Reflection Grating Spectrometer [RGS; (*26*)], Swift’s X-Ray Telescope [XRT; (*27*, *28*)], and the eROSITA instrument (*29*) on-board the Russian/German Spectrum-Roentgen Gamma (SRG) mission. NICER provided high-cadence monitoring data of the majority of the outburst, while XMM-Newton performed five exposures: one near the peak of the outburst (MJD 59287.34) and four after its luminosity decreased by a factor of ≳200 compared to the peak (on MJDs 59416.76, 59552.55, 59556.75, and 59615.36; see Fig. 1). A few Swift exposures were taken early in the outburst, and two sets of high-cadence monitoring—with one exposure per day lasting 1 to 2 ks—were performed for 15 and 20 days after the source faded in x-rays, i.e., between MJD 59391.19 to 59406.72 and 59525.39 to 59544.44, respectively (see Fig. 1A). eROSITA provided limits on x-ray flux from before the optical outburst and a detection during the decline phase.

##### NICER’s XTI

The NICER x-ray observatory has been operating on board the International Space Station (ISS) since July of 2017. Its primary instrument is the XTI, which is made up of 56 coaligned x-ray concentrators (XRCs) that focus x-rays into apertures of focal plane modules (FPMs). Each FPM consists of a single-pixel (nonimaging) silicon drift detector [SDD; (*30*)] with a field of view area of roughly 30 arc min^2^. At the beginning of science operations, 52 of 56 FPMs were active. The combination of these detectors provides a nominal bandpass of 0.3 to 12 keV with a peak effective area of ∼1900 cm^2^ near 1.5 keV. This large effective area in the soft x-rays, good spectral resolution (*E*/∆*E* ∼ a few tens) (see https://heasarc.gsfc.nasa.gov/docs/nicer/mission_guide/) combined with rapid maneuvering capability, makes NICER an ideal facility to perform spectral monitoring studies of variable soft x-ray phenomena like TDEs.

NICER started monitoring ASASSN-20qc on 13 February 2021 as part of an approved guest observer program (principal investigator: D.R.P., program number: 3139) performing multiple visits per day when possible. In this work, we include 162 observation IDs (obsIDs) totaling ≈300 ks of exposure time spread across 1921 good time intervals (GTIs) before any data screening was applied.

We started NICER data reduction with the raw data, i.e., unfiltered (uf) event files, publicly available on the High Energy Astrophysics Science Archive Research Center (HEASARC)’s archive: https://heasarc.gsfc.nasa.gov/cgi-bin/W3Browse/w3browse.pl. These data were reduced/cleaned using the NICER data reduction tools packaged as NICERDAS, which itself is part of the High Energy Astrophysics Software (HEASoft). We used HEASoft version 6.29c (released on 1 September 2021) with the latest NICER calibration files xti20210707 (20 July 2021). NICER version 2021-08-31 V008c was used.

NICER data are organized in the form of obsIDs where often each obsID contains multiple exposures taken over a period of 1 day. The initial data reduction to produce the unfiltered but calibrated event files (ufa), cleaned event files (cl), and GTIs was done on a per obsID basis using the standard nicerl2 tool. We used the following filters to extract the GTIs: nicersaafilt = YES, saafilt = NO, trackfilt = YES, ang dist = 0.015, st valid = YES, elv = 15, br earth = 30, cor range *=* “-,” min fpm = 38, under only range = “*-*,” overonly range = “*-*,” overonly expr = “NONE.” Except for the under only range, overonly range, and overonly expr parameters, the rest are the default values as recommended by the NICER data analysis guide: https://heasarc.gsfc.nasa.gov/lheasoft/ftools/headas/nimaketime.html. Instead of screening GTIs based on the underonly range, overonly range, and overonly expr values—which are proxies for screening out epochs of optical light leak and high particle background—we chose to screen them based on background-subtracted rates in the so-called S0-band (0.2 to 0.3 keV) and the HBG band (13 to 15 keV) as suggested by (*31*). Screening this way at a later stage, i.e., after computing the background spectrum, minimizes the total amount of data loss.

After extracting the unfiltered but calibrated (ufa) event files, calibrated (cl) event files, and the GTIs, we performed further analysis on a per GTI basis. First, we identify all the so-called “hot” detectors in each GTI, i.e., those affected by optical light leak and produce spuriously large amounts of charge. This is done by first estimating the mean count rate in the 0.0- to 0.2-keV band for each of the active FPMs in a given GTI. This array of 52 values is sigma-clipped, and detectors with values more than 4σ above the median of the sigma-clipped values are marked as hot for a given GTI. This information is also used further down the analysis pipeline while extracting time-resolved energy spectra (see Materials and Methods, “NICER time-resolved energy spectral analysis shows the same strong-weak outflow oscillatory pattern” section). Using the 3c50 background model (*31*), we estimated a background for each GTI by taking care to exclude the hot detectors. As per the recommendation given by (*31*), a given GTI is considered valid only if the following two conditions are met: (i) absolute value of the background-subtracted count rate in S0-band, i.e., 0.2 to 0.3 keV, is less than 10 cps, and (ii) absolute value of background-subtracted count rate in HGB band, i.e., 13 to 15 keV, is less than 0.1 cps. Finally, we also require that the observed 15- to 18-keV rate in a given GTI be within one SD of the distribution of all observed 1518-keV rates to exclude false flares. GTIs that do not satisfy these conditions are discarded and not included in further analysis. After the data screening, we were left with 239 ks of exposure spread over 364 GTIs. As recommended by the NICER data analysis guide (https://heasarc.gsfc.nasa.gov/docs/nicer/analysis_threads/cal-recommend/), we impose a conservative systematic uncertainty of 1.5%, i.e., systematic 0.015 in XSPEC, during all spectral modeling.

##### XMM-Newton EPIC

For all the XMM-Newton observations (obsIDs: 0852600301, 0891800101, 0891803701, 0891803801, 0893810701; principal investigator: D.R.P.; see table S4), we started our data reduction with their raw observation data files (ODFs). Using XMM-Newton’s science analysis software (xmmsas version 19.1.0), we reprocessed the EPIC-pn and MOS data using the standard tools epproc and emproc, respectively. During the first observation (obsID: 0852600301/XMM#1), because the MOS data were taken in the small window mode, there was no source-free area on the CCD (charge-coupled device) to extract a background from. Because of this reason, we decided to exclude MOS data from obsID 0852600301. The rest of the observations were taken in the full window mode with ample area to estimate a background. To enhance the signal to noise of the resulting spectra, we used both the pn and MOS datasets from the rest of the observations, i.e., 0891800101 (XMM#2), 0891803701, 0891803801 (XMM#3), and 0893810701 (XMM#4).

After producing the cleaned event files, we extracted GTIs without background flares (nonflare GTIs) using the 10- to 12-keV light curve as outlined in the XMM-Newton data analysis guide. For obsID 0852600301, we also extracted the instrumental GTIs for pn. By combining these two sets of GTIs (instrumental and nonflare), we extracted a set of GTIs without any background flares and when pn was actively operating. For the other four datasets, we extracted GTIs when background flaring was low and when both the pn and the MOS detectors were operating. The source spectra and event files were estimated using a circular aperture centered on the optical position of (ra, dec) = (04:13:02.450, −53:04:21.72) (J2000.0 epoch) and a radius of 33 arc sec. This radius corresponds to roughly 90% of the light from a point source as estimated by the fractional encircled energy of the EPIC-pn instrument. For the four datasets where the source decreased by more than two orders of magnitude, we used a smaller circular extraction region of 25 arc sec to minimize background contamination. Background spectra and events were extracted from two nearby circular regions, away from any point sources, each with radii of 45 arc sec. While extracting the spectra, we imposed additional filters of *#*XMMEA EP && (FLAG==0) && (PATTERN < =4) to only include the high-quality events for pn. For MOS, we used #XMMEA EM && (FLAG==0) && (PATTERN < =12).

The final spectra from obsID 0852600301 were grouped using the xmmsas tool specgroup to ensure a minimum of 20 counts per bin and an oversampling of 3. χ^2^ statistics were used for fitting spectral models. For the case of obsIDs 0891800101, 0891803701, 0891803801, and 0893810701, due to low counts, we used a minimum of one count per bin with an oversampling of three, and used the Cash statistic while spectral modeling. ObsIDs 0891803701 and 0891803801 were taken a few days apart so we modeled them together to improve the signal-to-noise.

##### XMM-Newton RGS

ASASSN-20qc was detected by the RGS only during the first observation (obsID: 0852600301). We use the latest pipeline RGS data products, which include the source and background spectral files, together with the instrument response files. We consider only the first-order spectra, which provide the highest signal-to-noise. To improve the signal-to-noise, we first stacked the RGS 1 and RGS 2 spectra. Then, using the ftgrouppha task, we binned the spectrum using the optimal scheme described by (*32*) with an additional requirement of at least one count per spectral bin. We then fit using the Cash statistics to exploit the high-energy resolution of the instruments. We focused the analysis in the observer-frame energy band of 0.35 to 0.75 keV, which is found to be clearly dominated by the source counts.

##### Swift XRT

Swift monitored ASASSN-20qc between 20 February 2021 and 26 November 2021. Between 20 February and 15 April, the source was observed once every 3 to 5 days (proposer: J.H.), while high-cadence (one visit per day) observations were made during 26 June to 11 July and 7 November to 26 November (proposer: D.R.P.). The duration of individual visits/exposures varied between 1000 and 2000 s.

We started our XRT data analysis with the raw data from the HEASARC public archives and reprocessed them using the standard HEASoft tool xrtpipeline. All XRT data were taken in the so-called photon counting (PC) data mode. We only used events with grades between 0 and 12 as recommended by the data analysis guide. Source events were extracted from an aperture of 30 arc sec. Background events were extracted in an annulus with inner and outer radii of 60 and 180 arc sec, respectively. We ensured that there were not any point sources within this background annulus.

With a mean background-subtracted 0.3- to 1.1-keV count rate of ≈1.6 × 10^−3^ counts s^−1^, ASASSN-20qc was barely detected in the individual exposures during the two high-cadence campaigns. Therefore, we combined the data from these epochs to extract one average flux measurement per campaign (see Fig. 1A).

##### SRG/eROSITA

eROSITA (*29*), the soft x-ray instrument aboard the Spectrum-Roentgen-Gamma mission (*33*), started the first of eight x-ray all-sky surveys (eRASS1 to eRASS8, each completed in 6 months) on 13 December 2019. It has since scanned over the coordinates of ASASSN-20qc in eRASS1 to eRASS4, although no source was detected with significance until eRASS4. Data were processed using eROSITA Science Analysis Software v946 [eSASS; (*34*)]. Photons were extracted around the source coordinates within a circular aperture of radius 30 arc sec, while background counts were extracted within an offset source-free circle of radius 156 arc sec.

In particular, in eRASS1, the telescope passed several times over ASASSN-20qc between 16 January 2020 (03:42:27 UTC) and 22 January 2020 (11,42:41) without detecting it (net exposure of ≈ 890 s). Assuming the spectral model obtained by NICER, a 3-sigma upper limit of the observed flux in rest frame 0*.*3 to 1*.*1 keV can be inferred at 1*.*5 × 10^−14^ erg s^−1^ cm^−2^.

The same spectral shape assumption for eRASS2 between 16 July 2020 (00:47:24) and 22 July 2020 (16:47:42, net exposure of 912 s), yields a 3-sigma upper limit of 6*.*6 × 10^−15^ erg s^−1^ cm^−2^, and for eRASS3 between 12 January 2021 (22:42:27) and 17 January 2021 (10:42:42, net exposure of 538 s), the limit is 1*.*2 × 10^−14^ erg s^−1^ cm^−2^. Data products were also extracted from the cumulative image combining all the first three eRASS scans, namely, a net exposure of 2339 s taken from 16 January 2020 to 17 January 2021: The stacked signal on the cumulative image allows the source to be as bright as 1*.*3 × 10^−14^ erg s^−1^ cm^−2^ (at 3σ). During eRASS4, the telescope scanned over ASASSN-20qc between 19 July 2021 (19:47:27 UTC) and 24 July 2021 (07:47:42). The source was detected with a total number of 14 counts in the 0*.*2- to 2*.*3-keV band in 663 s of net exposure. Fitting the spectrum with a diskbb model results in a median (and related 16th and 84th percentiles) observed flux of $72_{-1.9}^{+2.3}\times 10^{-14}$ erg s^−1^ cm^−2^ between rest frame 0*.*3 to 1*.*1 keV.

##### Optical and UV

ASASSN-20qc’s optical and UV data used here were obtained by Swift’s UV Optical Telescope [UVOT; (*35*)], the All-Sky Automated Search for SuperNovae [ASAS-SN; (*2*, *3*)], and the Transiting Exoplanet Survey Satellite [TESS; (*36*)]. To study the host galaxy, we also used archival optical, UV, and infrared (IR) data before 2020, i.e., before the optical and the x-ray outbursts (see Materials and Methods, “ASASSN-20qc’s host galaxy properties and black hole mass” section). Two optical spectra were obtained by the FLOYDS spectrograph: one near the peak of the optical light curve (*37*) and another after the source faded in x-rays. A high signal-to-noise optical spectrum was also obtained using the MagE spectrograph on the Magellan telescope (*38*) on 19 August 2021, i.e., after the x-ray outburst ended. Four more high Signal to Noise Ratio (SNR) optical spectra were obtained with LDSS-3 on Magellan. A description of the reduction procedures for these datasets is described below.

##### Swift UVOT and archival data

Swift UVOT (*39*) images were taken simultaneously with XRT observations (Materials and Methods, “Swift XRT” section). We reduce the observations using the uvotsource task in HEAsoft using a 5-arc sec aperture.

To estimate the host galaxy properties (see Materials and Methods, “ASASSN-20qc’s host galaxy properties and black hole mass” section) and to subtract its contribution to the Swift UVOT photometry, we compile the host galaxy spectral energy distribution (SED) using archival observations in the UV through IR bands. In the mid-IR, we use WISE (*40*) W1, W2, and W3 magnitudes. We also use DES (*41*) Kron magnitudes in g, r, i, z, and Y optical bands, while for the UV, we performed aperture photometry on the GALEX (*42*) NUV and FUV images with gPhoton package (*43*) using a 5-arc sec aperture.

##### ASAS-SN

ASAS-SN began surveying the sky in 2013 with the goal of identifying bright transients across the whole sky with an untargeted survey. From 2013 to 2017, ASAS-SN expanded from two to eight telescopes with *V*-band filters mounted on two mounts at two stations: Haleakala Observatory (Hawaii) and Cerro Tololo International Observatory (CTIO; Chile). In late 2017, we added 12 additional telescopes on three additional mounts at one at McDonald Observatory (Texas), one at South African Astrophysical Observatory (SAAO, South Africa), and a second station at CTIO. Our stations are hosted by the Las Cumbres Observatory Global Telescope Network [LCOGT; (*44*)]. Finally, in late 2018, we switched the eight original telescopes from *V*-band to *g*-band and we scan the entire visible sky down to *g* ∼ 18*.*5 mag nightly.

ASAS-SN units use FLI ProLine Cooled 2 *k* × 2 *k* CCD cameras with 14-cm aperture Nikon telephoto lenses. The units’ field of view is 4.5° on a side (20°^2^) with pixel size of 8.0 arc sec. Ideally, each observation epoch consists of three dithered 90-s exposures, although we are currently averaging 2.7 exposures per epoch due to scheduling and weather events. Furthermore, our observations are split between those taken with legacy *V*-band filters and *g*-band filters, which we plan to use going forward. The limiting *V*- and *g*-band magnitudes are *m* ∼ 17*.*5 and *m* ∼ 18*.*5, respectively. The original CTIO and Hawaii stations used *V*-band filters for observations up until the spring of 2019 when they were switched to *g*-band. The latter three stations have been using *g*-band filters since beginning of their operations.

##### TESS

Fortuitously, TESS captured the rise of the outburst in the optical band at an unprecedented cadence of one exposure every 30 min. We extracted a light curve following the procedures in (*45*). Briefly, we use the ISIS image subtraction software (*46*, *47*) to subtract a median “reference” image from individual TESS full frame images (FFIs) after convolving with a spatially variable kernel. This provides a correction for instrumental systematic errors due to pointing jitter, pointing drift from velocity aberration, and intrapixel sensitivity variations. Combined with some additional postprocessing steps to remove scattered light from the Earth/moon and nonuniform pixel sensitivity due to CCD “straps,” difference imaging has been shown to perform well in the background-dominated regime for TESS data [see, for example, (*48*)]. We then perform forced photometry at the location of the transient in the differenced TESS images using a model of the instruments Pixel Response Function at that location.

Previously, TESS captured the rise of a stellar TDE ASASSN-19bt and it was found that the optical brightness rose as *t*^α^, where α = 2*.*10 ± 0*.*12 (*49*). This value is similar to the “fireball” model commonly used to the fit the early rises of supernovae (*50*). Two other normal TDEs have had their rise slopes measured, albeit not with high-cadence TESS data. These are ASASSN-19dj, with a rise slope of $\alpha =1.90_{-0.36}^{+0.42}$ measured from ASAS-SN *g*-band data (*51*), and AT2019qiz, with a rise slope of α = 1*.*99 ± 0*.*01 measured from the bolometric light curve (*52*). Additionally, several other nuclear transients have had rise slopes measured with TESS, with flatter rises than these TDEs. These include the repeating TDE ASASSN-14ko, with a rise slope of α = 1*.*01 ± 0*.*07 (*53*) for the first flare observed by TESS when assuming a single power-law model, and α = 1*.*10 ± 0*.*04 and α = 1*.*50 ± 0*.*10 for the first and the second flares observed by TESS, respectively, when assuming a curved power-law model (*54*), and the ANT ASASSN-20hx, with a rise slope of α = 1*.*05 ± 0*.*06 (*55*).

We modeled ASASSN-20qc’s TESS light curve with a function of the form flux ∝ (*t* − *t*_0_)^α^ excluding data after MJD 59199, when the TESS background flux began to dominate the signal. We find a best-fit *t*_0_ of MJD = 59189*.*5 ± 0*.*3 and power-law index α of 1*.*35 ± 0*.*09, flatter than the three TDEs with measured rise slopes but steeper than either ASASSN-14ko or ASASSN-20hx.

##### FLOYDS optical spectra

Two spectra were taken by Las Cumbres Observatory (*44*), using the FLOYDS spectrograph on the 2.0 m Faulkes Telescope South. Spectra cover a wavelength range of 3500 to 10,000 Å at resolution *R* ≈ 300 to 600. Data were reduced using floyds_pipeline: https://github.com/lcogt/floyds_pipeline, which performs cosmic ray removal, spectrum extraction, and wavelength and flux calibration using standard IRAF/PyRAF routines as described in (*52*).

##### Magellan/MagE optical spectrum

ASASSN-20qc was observed on 19 August 2021 with the Magellan Echellete spectrograph (MagE), mounted on the Magellan Baade telescope located at Las Campanas Observatory, Chile. The observation was 3600 s long, and we used a 0.7 arc sec slit, which delivers an FWHM spectral resolution of 50 km s^−1^ at 4000 Å. The spectrum was reduced using the dedicated MagE data reduction pipeline (*56*, *57*). The flux calibration was performed using a spectrophotometric standard star Feige 110 observed during the night.

##### Magellan/LDSS-3 optical spectrum

We obtained four spectra in 2021 and 2022 (9 November 2021, 25 January 2022, 10 March 2022, and 16 August 2022) using the Low-Dispersion Survey Spectrograph 3 (LDSS-3) on the 6.5-m Magellan Clay telescope. Each set of observations included four 1200-s exposures of the target using a 0.9-arc sec slit and the VPH-All grism and was taken at parallactic angle. We used IRAF to reduce our LDSS-3 spectra following standard procedures, including bias subtraction, flat-fielding, 1D spectral extraction, wavelength calibration using a comparison lamp spectrum, and median combination of the individual exposures into a single final spectrum. We flux-calibrated our spectra using observations of spectrophotometric standard stars obtained on the same nights as our science spectra.

##### Radio

The position of ASASSN-20qc was observed by the Australian SKA Pathfinder (ASKAP) Telescope as part of the Rapid ASKAP Continuum Survey [RACS; (*58*, *59*)] and the ASKAP Variables and Slow Transients survey [VAST; (*60*, *61*)]. Overall, there are 11 observing epochs of ASASSN-20qc with ASKAP (1 with RACS and 10 with VAST). All of the observations were conducted at a central frequency of 887.5 MHz with a bandwidth of 288 MHz. The data were reduced using the VAST pipeline (*62*), and the full set of measurements is presented in table S1.

The source is detected in the first ASKAP epoch (RACS data) on 2019 May 4. It then seems to fluctuate (note also that the image rms level is also fluctuating between epochs) until the last ASKAP observation undertaken more than 2 years later on 22 August 2021. However, given the large flux density errors, it is not possible to determine statistically whether these observed fluctuations originate from variability of the source or are merely statistical fluctuations. There is also no significant change in the radio flux density in the two observing epochs following ASASSN-20qc’s optical discovery. The observed mean flux density of the source is 1.13 mJy, which translates to a luminosity of 3*.*7 × 10^37^ erg s^−1^.

### Optical spectral modeling and black hole mass

A fundamental parameter of probing the underlying physics is the black hole mass. We estimated this from the optical spectra. First, we rescaled all the spectra based on the photometric magnitude obtained from ASAS-SN automated pipeline. Then, we performed multi-component spectral decomposition using PYQSOFIT developed by (*63*) to measure the spectral information. A detailed description of the spectral decomposition method is given in (*64*). In brief, first, we corrected the spectrum for galactic extinction using the Milky Way extinction law of (*65*) with R_v_ = 3.1 and the (*66*) map. Then, the spectrum was transformed to the rest frame using a redshift of 0.056. The continuum was modeled using a combination of AGN power-law (*f*_λ_ = *A*λ^α^_λ_) and optical Fe II template from (*67*) to represent various blended Fe II emission lines. As stellar absorption features were not visible in the spectra, decomposition of the host galaxy contribution was not attempted. During the continuum fitting, all the strong Balmer emission lines were masked. The best-fit continuum model (PL + FeII) was subtracted, resulting in a pure emission line spectrum, which was modeled using multiple Gaussian components.

Emission lines were decomposed into broad and narrow components where each narrow component was modeled using single Gaussian with a maximum FWHM of 900 km s^−1^ to separate type 1 AGN from type 2 AGN following previous studies [e.g., (*64*)], while the broad components were modeled using two Gaussians each having FWHM larger than 900 km s^−1^. The velocity and width of the narrow components were tied together within an emission line complex. The broad Hβ and Hα components were modeled using two Gaussians, and [O III]λλ5007,4959 doublets were modeled using two Gaussians (one for the core and another for the wing). During the fit, the flux ratio of [O III] and [N II] doublets was fixed at the theoretical values, i.e., *F*(5007)/*F*(4959) = 3 and *F*(6585)/*F*(6549) = 3. All the emission lines in a given line complex were fitted together. The emission line information from spectral decomposition is given in table S2.

The FWHM of Hβ and Hα was measured to be 2108 ± 183 km s^−1^ and 2654 ± 441 km s^−1^, respectively, at the epoch of 11 January 2021 when the source was in the high state with monochromatic luminosity at 5100 Å (log *L*_5100_) of 43*.*97 ± 0*.*01 erg s^−1^. The AGN continuum was very blue. However, the source became fainter by 30 July 2021 with log *L*_5100_(erg s^−1^) = 43*.*84 ± 0*.*01. A strong, very broad component in Hα was found. He I 5876 Å, which was undetectable in January 2021, also became stronger. A very broad component (of FWHM ∼10*,*000 km s^−1^) in Hα is clearly visible in the August and the November spectra. Compared to the January spectrum, the later spectra show stronger Hβ and Hα emission lines. R4570, defined as the flux ratio between Fe II emission in the wavelength range of 4435 to 4685 Å to the Hβ broad component, is found to be ≈0.6. This value is typical for narrow-line Seyfert 1 galaxies (*68*).

The black hole mass was estimated from the monochromatic luminosity at *L*_5100_ and the width of the Hβ broad component using virial relation given by (*69*). The black hole mass estimates are found to be consistent in all epochs with an average value of **M*_•_* = 10^7.5^*M*_⊙_. The reported error bars on the black hole mass in table S2 only include measurement uncertainties. They do not include the uncertainty (*>*0.4 dex) associated with the virial relation due to the systematics involved in the calibration, unknown geometry, and the kinematics of the broad line region (BLR).

The virial mass measurements have several caveats and biases, e.g., the virial assumption evidence of which has been found in several AGNs with multiple emission lines and from the velocity resolve reverberation mapping, host galaxy subtraction, unknown geometry and kinematics, radiation pressure effect, and the use of different line width indicators: FWHM versus line dispersion [a detailed discussion can be found in (*70*)]. The validity of the virial assumption can be tested if, for an increase in the luminosity of the source, the line width decreases given enough response time. However, the limited dynamic range in the variability and the measurement errors in the spectral parameters, especially in the line widths, make this a challenging task for ASASSN-20qc.

### ASASSN-20qc’s location in the BPT and the WHAN diagrams suggests that it is an AGN

The BPT (*71*) and WHAN diagrams (*72*) are commonly used tools to classify different class of emission line objects based on the narrow line fluxes of [N II]6584/*H*α, [OIII]5007/Hβ, and Hα equivalent width (see table S2).

For the BPT diagram, we took the error weighted average of all the epochs if measurements are reliable better than 1-σ uncertainty. We note that due to strong blending of the narrow and the broad components in Hα and Hβ regions, estimation of the narrow components flux is challenging and the uncertainty in the flux measurement is large. By overplotting the Kewley (*73*) extreme starburst curve, Kauffmann (*74*) empirical relation, and Schawinski (*75*) separation line of LINER and AGNs, we find that ASASSN-20qc clearly falls in the AGN region.

For the WHAN diagram, we use the three measurements where the error bars are reasonable. Similar to the BPT diagram, the WHAN diagram also suggests that ASASSN-20qc is an AGN.

### ASASSN-20qc’s host galaxy properties and black hole mass

To estimate the host properties, we model the pre-flare SED (table S3) using the flexible stellar population synthesis module [FSPS: (*76*)]. We also included a nonstellar power-law continuum, available on FSPS, that represents an AGN contribution to the SED before ASASSN-20qc. We use the Prospector (*77*) software to run a Markov chain Monte Carlo (MCMC) sampler (*78*). In the Prospector fitting, we assume an exponentially decaying star formation history (SFH) and a flat prior on the six free model parameters: stellar mass (*M*_★_), stellar metallicity (*Z*), color excess due to dust extinction E(B-V), assuming the extinction law by (*79*), the stellar population age (*t*_age_), the e-folding time of the exponential decay of the SFH (τ_sfh_), and the fraction of the total light that is produced by the AGN (*f*_AGN_). From the best-fit template spectrum, we derive the following:$\text{log}\left(M_{★}/M_{⊙}\right)=10.13_{-0.01}^{+0.02},\text{log}\left(Z/Z_{⊙}\right)=-0.55_{-0.04}^{+0.02},E(B-V)=0.01_{-0.01}^{+0.01}\;\text{mag}$, $t_{\text{age}}=3.23_{-0.28}^{+0.22}$ Gyr, $\tau_{\text{sfh}}=0.43_{-0.05}^{+0.04}$ Gyr, and $f_{\mathrm{AGN}}=0.05_{-0.01}^{+0.01}$. The color excess is in complete agreement with the galactic value *E*(*B* − *V*) = 0*.*0137 mag (*80*) requiring no additional extinction from the host galaxy. We estimate the host galaxy fluxes in the UVOT bands from the posterior distribution of the population synthesis models. The host contribution was then subtracted from the UVOT measured photometry (table S3). The uncertainty on the host galaxy model was propagated into our measurements of the host-subtracted fluxes.

We also estimate *M*_•_ from the host galaxy mass by applying the (*81*) relation: log *M*_•_/*M*_⊙_ = 7.56 + 1.39[ log (*M*_★_/*M*_⊙_) − 10.48]. This results in log $M_{∙}/M_{⊙}=7.06_{-0.01}^{+0.02}\pm 0.79$, where 0.79 dex is the intrinsic scatter of the relation. This values agrees with the virial mass measurements in Materials and Methods, “Optical spectral modeling and black hole mass” section, and fig. S2.

#### ASASSN-20qc dominates the x-ray emission in NICER/XTI’s field of view

NICER/XTI is a single-pixel (nonimaging) instrument with a field of view of roughly 30 arc min^2^ in area. Therefore, to rule out a contaminating point source, we extracted an image from the combined Swift/XRT images, which shows a single point source coincident with the optical coordinates. This demonstrates that ASASSN-20qc dominated the x-ray emission in NICER’s field of view, and contamination by other sources was negligible.

### X-ray energy spectral modeling: NICER and XMM-Newton detect an ultrafast outflow

We started our x-ray energy spectral analysis with an average spectrum derived from the first few weeks of NICER data. This spectrum was soft with essentially no source x-ray events above ≈1.1 keV. Following this revelation early in the outburst, we requested for a 50-ks XMM-Newton observation to get a deep x-ray snapshot of ASASSN-20qc (XMM#1). For this, we triggered an approved XMM-Newton guest observer program 085260 (principal investigator: D.R.P.).

We then turned our focus to the XMM-Newton dataset for a detailed spectral study. Similar to the earlier NICER spectrum, XMM-Newton/EPIC-pn spectrum was also soft, with the background becoming comparable to the source beyond roughly 1.5 keV. To avoid uncertainties from background estimation and to match with NICER’s bandpass, we only considered the energy range of 0.3 to 1.1 keV for further analysis. For spectral modeling, we used the XSPEC spectral fitting package (*82*) and a Python interface to XSPEC known as PyXspec.

We started by modeling the XMM-Newton/EPIC-pn spectrum from XMM#1 with simple phenomenological models: a thermal accretion disk modified by Milky Way’s neutral absorbing column and a power-law modified by Milky Way’s neutral absorbing column of 1.2 × 10^20^ cm^−2^. The Milky Way column along the direction of ASASSN-20qc was estimated using the HEASARC nH calculator: https://heasarc.gsfc.nasa.gov/cgi-bin/Tools/w3nh/w3nh.pl (*83*). These two models were defined as tbabs*zashift(diskbb) and tbabs*zashift(pow) in XSPEC. The zashift component accounts for the host galaxy redshift of 0.056. The former model resulted in a χ^2^ of 187.5 with 19 degrees of freedom (df), while the latter yielded a χ^2^ of 2026.8 with 19 df. In both cases, strong systematic residuals were evident (see fig. S4, A and B). In the case of the power-law model, the best-fit photon index was roughly 6. Adding a Gaussian to the power-law model improves the fit, resulting in a χ^2^/df of 13.4/15 (fig. S4C). However, again, the best-fit power-law index is extreme with a value of 8.5 ± 0.2. Typically, AGN has a power-law index value of ≈1.8 with extreme values up to 3 (*84*). An index value of 8.5 is unphysical because, when extrapolated to lower energies, it would imply an unrealistically high intrinsic luminosity. Also, such a steep index can be explained by the fact that in the narrow bandpass of 0.3 to 1.1 keV, we are fitting the Wien’s portion of the black body emission, which naturally leads to a steep index when modeled with a power-law. Using a thermal disk plus a power-law model, i.e., tbabs*zashift(diskbb + pow), does not improve the fit significantly when compared with the disk only model (fig. S4D). The best-fit power-law normalization value is pushed to a value close to zero. Given the soft nature of the spectrum, we proceed with the thermal model for the continuum, i.e., tbabs∗zashift(diskbb), which gives an inner disk temperature of roughly 90 eV.

The residuals show a systematic behavior with an excess near 0.65 keV and a deficit near 0.85 keV (fig. S4A). These are remarkably similar to the residuals seen in the early x-ray spectra of TDE ASASSN-14li, which has been interpreted as a newly launched UFO (*85*). Similar residuals at energies between 0.3 and 10 keV have been seen in x-ray spectra of numerous AGN, and these are also interpreted as UFOs [see, e.g., (*5*, *86*, *87*)]. Motivated by these previous studies, we fit the residuals by first adding an absorption line. As customary in x-ray spectral analysis in XSPEC, we model the absorption feature adding an inverted (negative intensity) Gaussian line. This is mathematically equivalent of including a multiplicative Gaussian absorption line. The overall χ^2^/df improved from 187.5/19 with tbabs*zashift(diskbb) to 43.1/16 with tbabs*zashift(diskbb + gauss absorption) (fig. S4E). Except for the redshift of the host galaxy and the Milky Way column, all the other model parameters were allowed to be free for the above fits. Because there are still systematic deviations near 0.65 keV (fig. S4E), we added a Gaussian emission line, which improves the fit to 11.8/13 (fig. S4G). We also fitted by adding the Gaussian emission line first, which yielded a χ^2^/df of 50.7/16 (fig. S4F). We also experimented with considering a multiplicative Gaussian component for the absorption line, modeled as “gabs” in XSPEC, tbabs∗zashift(gabs∗diskbb + gauss), which yielded a similar good fit with χ^2^/df of 11.8/13 (fig. S4H).

Encouraged by the above Gaussian fits, we implemented in XSPEC a physically motivated XSTAR (*88*) table model consisting of ionized gas between the illuminating central x-ray source and us, the observer (see details of the XSTAR model in Materials and Methods, “XSTAR energy table models” section). This model gives a good fit with χ^2^/df of 19.9/16, and the best-fit parameters imply the presence of a UFO moving toward us at ≈0.33*c*, where *c* is the speed of light (fig. S4I).

To rule out that the feature is not an artifact of limited bandpass, we also fit the 0.3- to 2.0-keV bandpass of EPIC-pn spectrum. This gave results consistent with the above parameters. We also tested if a power-law component maybe present after adding the UFO. Adding the power-law improved the χ^2^ by five with two additional dfs. On the basis of the Akaike information criterion, we conclude that a power-law in not statistically required by the data.

We also analyzed the combined RGS1 and RGS2 spectrum (0.35 to 0.75 keV) from XMM#1, which has roughly a factor of 30 higher resolving power than EPIC-pn. We find clear evidence for two narrow outflow components in the RGS spectrum. These can be modeled with an XSTAR table model with a velocity broadening of 100 km s^−1^. The derived column density and ionization parameter values are $\left[\left(4.6_{-3.2}^{+4.5}\right)\times 10^{21}{\text{cm}}^{-2},2.7_{-0.4}^{+0.5}\;\text{erg}\;s^{-1}\;\text{cm}\right]$ and $[\left(2.0_{-1.2}^{+7.9}\right)\times 10^{21}{\text{cm}}^{-2}1.5_{-0.9}^{+0.5}\;\text{erg}\;s^{-1}\;\text{cm}]$, for the two outflows. The velocity shift is consistent with an outflow velocity of ∼1000 km s^−1^ for the first one and with zero for the second one. These parameters are consistent with warm absorbers (WAs) typically detected in local Seyfert galaxies [e.g., (*89*)] and are discussed in detail in a separate paper (*90*).

The XMM-Newton dataset does not allow us to reliably perform a joint RGS and EPIC-pn fit for several reasons. (i) Because the spectrum is extremely soft, this limits the RGS band to 0.35 to 0.75 keV and the EPIC-pn to 0.3 to 1.1 keV. In this overlapping, very soft band, the two instruments are known to have significant cross-calibration uncertainties [e.g., (*91*)]. (ii) The very soft source spectrum and limited energy band does not allow us to use the typical method of using the RGS data below 1.5 to 2 keV and the EPIC-pn from 1.5 to 2 keV up to 10 keV. (iii) If we perform a joint fit using the RGS between 0.35 to 0.75 keV and the EPIC-pn between 0.7 to 1.1 keV, the source continuum would not simply extend from the RGS to the EPIC-pn band. This is because the broad absorption feature would significantly affect the continuum shape and intensity in the 0.7- to 1.1-keV band. Consequently, we cannot use a simple cross-normalization constant between the two instruments. Therefore, for the aforementioned reasons, we performed separated fits to the EPIC-pn and RGS data.

#### The broad absorption residuals cannot be explained with slow outflows

While a detailed study of these WAs will be presented in a separate work (*90*), we address three specific questions here. First, can the slow-moving outflows found in RGS data explain the residuals seen in the low-resolution EPIC-pn and NICER spectra? To answer this, we fit a model consisting of thermal emission modified by two slow outflows to the pn spectrum. The parameters of the outflows were constrained to be within 99% of the best-fit values from RGS modeling. The exact model we used was tbabs * WA1 * WA2 * zashift(diskbb), where WA1 and WA2 are the two WAs. This model gives a very poor best-fit χ^2^/df of 79.6/15 with similar residuals as without WA1 and WA2 (fig. S4J). From this, we conclude that the x-ray spectral residuals seen in fig. S4A cannot be explained by the two slow outflows seen in the RGS data.

Second, can we explain the residuals in fig. S4A with a third WA? Adding a third slow outflow to the EPIC-pn data improves the fit and results in a χ^2^/df of 19.2/13, which is, however, still worse than the case of a single mildly relativistic outflow. The best-fit column density and ionization parameter of this third WA are $1.2_{-0.4}^{+0.6}\times 10^{23}{\text{cm}}^{-2}$ and $4.1_{-0.6}^{+0.5}\;\text{erg}\;s^{-1}\;\text{cm}$, respectively. Because it is a slow outflow by definition and EPIC-pn spectrum does not have the sufficient spectral resolution in the soft x-ray band to discriminate velocity shifts lower than ∼10,000 km s^−1^, its velocity shift was fixed to zero. Now, the presence of such a putative very high column third WA component in the EPIC-pn should lead to intense narrow absorption lines and ionization edges in the RGS data in the 0.35- to 0.75-keV band. To test for this, we modeled the RGS data with three WAs, with the third WA having the same parameters inferred from the EPIC-pn data. Adding the third WA to the RGS data provides a much worse fit (C-stat/df of 677.7/337) with respect to just two WAs components (C-stat/df of 523.9/337). Therefore, we conclude that the presence of a third very high column WA is excluded by the RGS data and that the broad residual feature in fig. S4A is better interpreted as broad OVIII resonant absorption, with a blueshift of ∼0.3*c* and broadening of ∼30,000 km s^−1^, instead of OVII-VIII edges with a low velocity shift and broadening of ∼100 km s^−1^.

Finally, we also address the question: how does the inclusion of the two RGS WA components in the EPIC-pn spectrum alter the inferred properties of the UFO? To answer this, we fit two models, one consisting of a thermal component modified by two slow outflows and one UFO, i.e., tbabs∗WA1∗WA2∗UFO∗zashift(diskbb) in XSPEC, and another with a thermal component modified by the UFO alone, i.e., tbabs∗UFO∗zashift(diskbb) in XSPEC. In the former case, we constrained the parameters of the two WAs to be within 99% of the best-fit RGS values. The best-fit column, ionization parameter, and the line of sight velocity values of the UFO with and without the slow components are consistent with each other within the 90% uncertainties.

Thus, we conclude that the two WAs detected in the RGS spectrum do not affect the properties of the UFO, and we do not include them in further modeling.

#### A spectral model with two thermal components akin to quasi-periodic eruptions is ruled out

We also tested if two slow outflows and two thermal components can fully describe the x-ray spectrum. The motivation for two thermal components comes from studies of quasi-periodic eruptions (*92*, *93*). For this, we fit tbabs∗zashift[WA1∗WA2∗(diskbb + diskbb)] to the pn spectrum. Similar to the above analysis, the parameters of WA1 and WA2 were constrained to be within the 99% uncertainty of the best-fit values from RGS. This resulted in a best-fit χ^2^/df of 69.8/13 with systematic residuals between 0.55 and 1 keV (fig. S4K). We also experimented with bbody for the second thermal component but that did not improve the fit (fig. S4L). From this analysis, we concluded that two thermal components plus two slow outflows cannot explain ASASSN-20qc’s x-ray spectrum.

#### Relativistic reflection is disfavored

X-ray reflection in the inner regions of the accretion flow can, in principle, produce residuals similar in shape to those seen in fig. S4A. The typical picture in the AGN context is that there is a compact corona that emits a nonthermal (power-law) x-ray spectrum. Part of this coronal emission reflects off the inner accretion disk, where general relativistic effects are strong, to produce relativistically broadened emission features [see, for example, (*94*) and references therein]. This scenario is disfavored for ASASSN-20qc due to the lack of an obvious power-law continuum emission from a putative compact x-ray corona around the black hole, required to effectively illuminate the accretion disk.

Alternatively, it has been argued that x-ray reflection can also occur in the absence of a compact corona (*95*). For example, ASASSN-18el is a nuclear outburst lasting for over 3 years (*95*). Its x-ray spectra during the early phases of the outburst were soft with negligible emission beyond 3 keV, i.e., a weak corona. When fit with a thermal model, these spectra result in broad residuals between 0.7 and 2 keV [see figure 2 of (*95*) and figure 2 of (*96*)]. Masterson *et al*. (*95*) have modeled ASASSN-18el’s residuals with relativistically broadened reflection. The underlying picture in their model is that the inner accretion disk produces the overall thermal continuum, and because the system is accreting near the Eddington limit, a powerful outflow is launched off the disk. Thus, the total emission reaching us consists of two components: direct disk/thermal emission and thermal emission reflected off the outflow. Because the outflow is launched from very close to the black hole, the reflected emission is subject to relativistic effects. For this scenario, Masterson *et al*. (*95*) developed xillverTDE, a reflection model in which the incident spectrum is a soft thermal continuum instead of a power-law/nonthermal emission from a corona. We also considered xillverTDE for ASASSN-20qc and, as a starting point, applied a model similar to ASASSN-18el: tbabs * ztbabs * [zashift(diskbb) + relconv(xillverTDE)]. Here, diskbb and xillverTDE are the direct and reflected emission, respectively. Relconv accounts for relativistic broadening, which is necessary given the broad residuals. Considering all the spectra corresponding to the phases where the residuals are strong, i.e., the so-called min phases in ODR curve (see Materials and Methods, “ODR timing analysis” section), this model results in a combined χ^2^/df of 99.7/72. Here, we allowed Fe abundance, inner disk inclination, density, and ionization parameter of the material facilitating reflection to be free across all spectra. The redshift of the xillverTDE was fixed at the redshift of the host galaxy. Taken at face value, this reflection model implies an improvement in χ^2^ of 11.6 at a expense of 36 additional dfs when compared with the UFO model (table S5). Tying the Fe abundance across all the min spectra results in a χ^2^/df of 96/81. Tying the density of the reflecting material or the ionization parameter across all the spectra results is a worse fit (reduced χ^2^ *>* 2.5). Following our in-depth spectral modeling with xillverTDE, we disfavor the reflection model for this source for the following reasons.

1) In ASASSN-18el, Masterson *et al*. (*95*) suggested that a clumpy outflow could provide a reflecting medium. However, in the case of ASASSN-20qc, the best-fit reflection model does not require an outflow, i.e., the redshift of xillverTDE component is fixed at the host galaxy value of 0.056 while modeling. Allowing it to be free results in positive (redshifted) values, which would imply material falling into the black hole, and is therefore inconsistent with the reflection scenario.

2) Photons emitted from an accretion disk may be gravitationally bent over the black hole and subsequently illuminate the “far side” of the accretion flow. It is possible therefore that the disk’s thermal emission could itself be the source of a reflection spectral component. However, only the photons emitted very close to the black hole undergo sufficient ray bending to illuminate the far side of the accretion disk, and for a Schwarzschild black hole, the fraction of the liberated energy that is then reabsorbed is on the order of 1% (*96*). For higher black hole spins, this fraction increases, but is still limited to ∼10%, even for the most rapidly rotating (*a* = 0*.*99) black holes [see figure 2B of (*96*)]. From fitting the XMM-Newton and the NICER spectra with relativistic reflection, i.e., tbabs∗zashift(diskbb + relconv∗xillverTDE) in XSPEC, we find that the reflected component dominates the observed flux by a factor of few to up to 10 over the direct thermal component. This is inconsistent with reflection in the gravitational light bending and disk illumination scenario for a standard disk. To produce such high fluxes in the reflected component would require a fine-tuned disk geometry, which seem contrived.

3) Lack of a suitable interpretation for the observed variability. For instance, a putative disk precession was already disfavored. Furthermore, there is no clear separation between the best-fit parameters (disk inclination, column, etc.) between the min and max spectra.

4) Finally, from a statistical point of view, the reflection model improves χ^2^ only marginally for a large number of additional parameters. Therefore, the reflection model is not statistically superior to the UFO absorption model for this source.

#### The outflow is not an artifact of averaging data

The absorption feature near 0.85 keV is present in the majority of the phase-resolved NICER spectra. Because these spectra are obtained by combining data over a certain period (a few days in some cases), it is, in principle, possible that 0.85 keV could be an artifact of varying spectral properties (blackbody temperature and normalization). However, the presence of the same residuals in a few hours of XMM-Newton snapshot affirms that the feature near 0.85 keV is not an artifact of a varying spectrum.

#### On the nondetection of an emission line

We note that the ratios of the data with respect to the continuum may, in principle, be reminiscent of a P-Cygni profile, where the emission appears redshifted and the absorption appears blueshifted [e.g., (*5*, *97*)]. However, we do not find a statistically significant requirement for an emission component after including the XSTAR absorption table.

There can be two reasons why the data do not require an additional emission feature associated with a putative P-Cygni profile. One reason is that the variable outflow observed along the line of sight is in the form of a cloud or its physical extent is limited. Therefore, its emission would be expected to be intrinsically weak.

A second reason may be that the outflow could be geometrically broad and extended, but the emission line arising from a ∼0.3*c* outflow would be so broadened due to Doppler effects (with a width of up to ∼1 keV), resulting in a very marginal contribution over the continuum. The narrow energy band (*E* = 0.3 to 1.1 keV) of the source spectrum and the limited SNR of the observations would make the detection of such a very broad emission feature currently impossible. The detection of such a broad and faint emission feature would require x-ray spectrometers with a much higher effective area and energy resolution than currently available, consistent with those proposed for the Athena and the Lynx x-ray observatories.

#### Computing the observed luminosity versus time curve

The individual NICER GTIs do not have enough counts to compute the observed luminosity and other parameters of the outflow. Therefore, we use the mean count rates and the observed flux measurements from time-resolved spectra from table S5 and Materials and Methods, “NICER time-resolved energy spectral analysis shows the same strong-weak outflow oscillatory pattern” section. We compute the 0.3- to 1.1-keV observed luminosity of a GTI by scaling the 0.3- to 1.1-keV count rate to the value of luminosity in a given epoch. The resulting curve is shown in Fig. 1A.

### The temperature of the ASASSN-20qc blackbody continuum emission

The x-ray spectrum of ASASSN-20qc is very well modeled with a single blackbody continuum component with a temperature of *T* ≃ 90 eV. This is consistent with what is usually found for x-ray spectra of TDEs [see the right panel of figure 2 of (*98*)]. This phenomenological modeling is required to characterize the continuum shape and normalization, but we do not derive physical conclusions from it. The phenomenological blackbody emission is only used as the input ionizing continuum in the XSTAR photoionization code to calculate the absorption tables (see Materials and Methods, “XSTAR energy table models” section, for more details on table models).

From a phenomenological point of view, we note that a hybrid accretion disk solution combining an ADAF-type hot flow and a standard thin disk is often suggested as a description of the emission for low-luminosity AGN [e.g., (*99*)]. Depending on the thin disk truncation radius and the temperature, geometry, and extent of the inner ADAF, it is plausible that black body optical/UV disk photons are up-scattered by the hot ADAF gas, similarly to the putative x-ray corona in more luminous AGN. In the case of ASASSN-20qc, being the disk quite limited in spatial extent, the resultant spectrum would most likely be approximated with a single blackbody with increased temperature.

Moreover, classical estimates based on steady-state accretion theory do not apply to a disk system undergoing a large amplitude outburst like ASASSN-20qc, where the disk is not in inflow equilibrium. A disk system out of the steady state can have a higher surface density in its innermost regions, leading to a higher temperature at the inner edge of the disk. This is particularly true for TDEs around higher mass black holes, where the incoming star’s tidal radius approaches the black hole’s Innermost Stable Circular Orbit (ISCO).

To further demonstrate that the blackbody temperature inferred for ASASSN-20qc is consistent with a TDE-disk system, we simulate mock 0.3- to 1.1-keV x-ray spectra for time-dependent and fully relativistic accretion disk systems, using the techniques described in (*100*, *101*). These mock spectra were produced using full-photon ray-tracing calculations and therefore include all leading order relativistic effects. By fitting these mock x-ray spectra with a phenomenological blackbody profile, a temperature of the spectrum can be extracted. We compute the fitted temperature of the x-ray spectrum, produced for a black hole mass *M* = 10^7^*M*_⊙_ and disk mass *Md* = 0.5*M*_⊙_, for a range of black hole spins and disk-observer inclination angles. Note that this fitted temperature will differ from the physical temperature of the inner disk primarily due to the effects of Doppler and gravitational shifts, and the color correction of the disk emission [as discussed in (*101*)]. We only consider inclination angles consistent with the obscuring UFO scenario (θ_inc_ *<* 22°). Each x-ray spectrum was produced at a time corresponding to the peak of the disk bolometric light curve. We find that a temperature of 85 eV is within an acceptable parameter space.

### A single clumpy outflow is disfavored

A steady and clumpy outflow launched at the onset of the x-ray outburst is disfavored due to the presence of the quasi-periodicity. This is because, to produce the observed modulations in the ODR curve, the clumps would need to be arranged in a preferred manner around the central black hole. This is highly unlikely for any intrinsically random distribution of clumps.

The timing analysis in Materials and Methods, “ODR timing analysis” section, already computes the odds of this happening to be less than 1 in 50,000.

### The outflow is present even at 200 times lower x-ray luminosity

To test the strength of the outflow as a function of observed luminosity, we also obtained XMM-Newton exposures after the initial outburst ended. While the first few XMM-Newton exposures were too short, i.e., low signal-to-noise, we detect the same UFO signature in XMM#3. The C-stat/df without the UFO was found to be 125.8/89. Including the outflow improved the C stat/df to 97.9/86, i.e., ∆C-stat of 27.9 for three additional dfs, which corresponds to a confidence level of 99.99%. The unfolded spectrum along with the residuals is shown in fig. S11.

### Extracting composite spectra from NICER data

To study the spectral properties during the epochs of ODR maxima and minima, we combined exposures and obtained composite energy spectra. While combining the data, we remove the detectors marked as hot based on the 0.0- to 0.2-keV count rate as described above. The main steps for extracting time-resolved NICER energy spectra are as follows.

1) First, we extract the combined ufa and cl event files using the start and end times of all GTIs within a given epoch.

2) Then, we use the 3c50 model on these combined ufa and cl files to estimate the average background and source spectra. All the detectors marked as hot at least once in any of the individual GTIs are excluded.3) Using the tools nicerarf and nicerrmf, we extract arf and rmf for each epoch.4) Then, we group the spectra using the optimal binning criterion described by (*32*), also ensuring that each bin has at least 25 counts

### NICER time-resolved energy spectral analysis shows the same strong-weak outflow oscillatory pattern

We modeled the energy spectra of the individual maxima and minima in the ODR curve using the ionized outflow model. The results are shown in table S5. It is evident that both the absorbing column and the ionizing fraction are more than an order of magnitude higher during the epochs of ODR minima than during the maxima (fig. S10).

Some of the spectra, during some maxima, did not require an outflow component. In these spectra, the χ^2^/df was close to 1 with a thermal component alone. These are marked by shaded orange regions in fig. S10.

### Outflow energetics

As conventionally done in the literature, we conservatively estimate the outflow launching radius to be the distance at which the observed velocity is equivalent to the escape velocity from the SMBH [e.g., (*97*, *103*,*104*)]: *r* = 2*GM*_•_*/v*_out_^2^. This can be written also in units of the gravitational radius *r_g_* = *GM*_•_*/c*^2^ as *r* = 2(*v*_out_*/c*)^−2^*r_g_*. Considering a black hole mass of log(*M*_•_/*M*_⊙_) = 7.4, we estimate a gravitational radius of *r_g_* = 3*.*7 × 10^12^ cm. The mass outflow rate can be estimated using the following equation: *M*^˙^out = 4π*C*_f_*rN_H_*μ*m_p_v*_out_, where *N_H_* is the column density, μ = 1*.*4 is the mean atomic mass per proton, *m_p_* is the proton mass, and C_f_ is the global covering fraction typically assumed to be 0.5 for AGN disk outflows [e.g., (*105*–*107*)]. Then, the kinetic power of the outflow can be estimated using the following formula: *E*^˙^_out_ = 1*/*2 *M*^˙^out*v*_out_^2^. We also calculated the ratio between the outflow kinetic power and the unabsorbed luminosity in the 0.3- to 1.1-keV band, *E*^˙^_out_*/L*. From this, we also estimated the ratio between the mass outflow rate and the mass accretion rate *M*^˙^_out_*/M*^˙^_acc_, considering *M*^˙^_acc_ = *L/*η*c*^2^ and a typical radiative efficiency η = 0*.*1.

Using the best-fit parameters reported in tables S4 and S5, we show the estimates for the outflows detected in the time-resolved NICER analysis and in the XMM-Newton spectra in table S6. We note that we are not reporting error bars in our calculations, as they are considered as order-of-magnitude estimates. However, the important point here is not the absolute value of each parameter, but the difference between the average parameters in the min and max phases of the ODR, which is independent on the model assumptions and overall uncertainties. For the NICER min phases, we derive the following average quantities: launching radius *r* ≃ 18*r_g_*, mass outflow rate *M*_out_ ≃ 0.002*M*_⊙_/year, kinetic power *E*^˙^_out_ ≃ 6 × 10^42^ erg s^−1^, a ratio *E*^˙^_out_*/L* ≃ 10%, and a ratio *M*^˙^_out_*/M*^˙^_acc_ ≃ 18%. For the NICER max phases, we derive the following average quantities: launching radius *r* ≃ 18*r_g_*, mass outflow rate *M*_out_ ≃ 0.0003*M*_⊙_/year, kinetic power *E*^˙^_out_ ≃ 0*.*9 × 10^42^ erg s^−1^, a ratio *E*^˙^_out_*/L* ≃ 1%, and a ratio *M*^˙^_out_*/M*^˙^_acc_ ≃ 2%. Comparing the estimates in the NICER min and max phases, we see that the outflow launching radius is consistent, but the overall mass flux and energetics are one order of magnitude lower for the latter.

From these estimates, we can infer some information regarding the potential impact of the outflow on the accretion flow and on its host galaxy feedback. The outflow is launched from the disk along the boundary of the accretion flow and the much less dense, but highly magnetized funnel. Therefore, simulations show that it will not significantly interfere with the accretion flow. This is supported by the estimate of the instantaneous mass flux, reported in table S6, which is limited to about 20% of the mass accretion rate. On the other hand, given its mildly relativistic velocity, the outflow is found to have a power reaching up to about 10% of the peak luminosity. This relatively high power suggests that it could temporarily drive feedback into the host galaxy (*108*).

Regarding the XMM-Newton spectra, they are not exactly placed in the min and max phases, so their values are not directly comparable to the NICER time-resolved analysis. From table S6, we see that the outflow was statistically detected in two of four XMM-Newton phases. In XMM1, the outflow has values comparable to the NICER max phases. Instead, for XMM3, which was performed much later, during the low-luminosity state of the source, the values of the outflow mass flux and energetics seem comparable to the NICER min phases. However, we note that the high values of the ratios *E*^˙^_out_*/L* and *M*^˙^_out_*/M*^˙^_acc_ in XMM3 may indicate that the outflow could likely be magnetically accelerated and that the disk radiative efficiency may be lower than the typical value assumed for the high-luminosity state of the source.

### ODR timing analysis

It is evident from NICER’s soft x-ray light curve that the source underwent a major outburst, increasing by a factor of *>*600 and thereafter decreasing by a factor of roughly 200. Also, near the peak, i.e., between MJD 59260 and 59370, the source is variable. With an unprecedented high-cadence soft x-ray coverage, NICER data provide a unique opportunity to study the coevolution, if any, of the UFO with the accretion (thermal continuum). Therefore, to track the evolution of the outflow with respect to thermal continuum, we extracted a hardness ratio defined as the ratio of the count rate in the outflow band, 0.75 to 1.0 keV, over the continuum band, 0.3 to 0.55 keV, and refer to it as the ODR. The ODR versus time plot shows repeated flares that appear to recur roughly once every 8.5 days (see Fig. 2A). As the ODR is inversely proportional to the strength of the outflow, a lower value would imply a stronger outflow and vice versa. To verify that the LSP signal near 8.5 days in Fig. 2B is robust against the choice of the period-finding algorithm, we also implemented the phase dispersion minimization algorithm (*109*) and the weighted wavelet Z-transform (*110*, *111*) (see figs. S7 and S8, respectively). They both found the signal at the same frequency as the LSP, confirming the signal’s robustness against algorithm selection. For all further timing analysis, we use the LSP throughout the rest of the paper. To test the statistical significance of the peak near 8.5 days in the LSP, we first establish that the power values in the LSP are consistent with white noise.

#### Values in the LSP are consistent with white noise

To test for the presence of a quasi-periodicity in the ODR curve, we computed its LSP (*6*, *7*). The LSP was sampled at *N_i_* independent frequencies as per equation 13 of (*7*). Consistent with the ODR curve, the highest peak in the LSP is near 8.5 days (see Fig. 2B). To assess the global statistical significance (false alarm probability) of this LSP excess near 8.5 days, we perform more analyses. We first turn our focus to understand the nature of the underlying noise in the LSP because an accurate characterization of the noise in the LSP is of utmost importance for estimating the statistical significance.

The ODR tracks the outflow’s relative strength compared to the thermal continuum. By construction, because we are dividing by 0.3- to 0.55-keV flux, i.e., the band dominated by accretion-driven fluctuations, we expect to suppress any red noise present in the continuum. By eye, the ODR values between the flares appear to be roughly constant. To verify this more rigorously, we performed additional statistical tests.First, we normalize the LSP to have a mean value of 1 by dividing the LSP with the mean of all power values excluding bins near 8.5 days. We then compute the empirical distribution function (EDF) and the probability density function (PDF) of these LSP power values and compare them with the expected 1-*e*^−*z*^ distribution expected for LSP if the power values were derived from white noise (*6*). Here, *z* is a variable representing LSP powers. EDF and PDF shown in fig. S9 (A and B) are qualitatively consistent with the expected exponential distribution.

Next, we investigate the nature of the distribution of LSP powers quantitatively. We performed the Kolmogorov-Smirnov (K-S) and the Anderson-Darling goodness-of-fit tests under the null hypothesis that the LSP powers are white, i.e., their values between ≈1.5 and 100 days, except for bins near 8.5 days, are exponentially distributed. The underlying principle behind these statistics is that they measure the maximum deviation between the EDF of the data and that of a comparison distribution. Therefore, the better the distribution fits the data, the smaller these statistic values will be.

We computed the K-S statistic using the EDF of LSP powers and the expected 1-*e*^−*z*^ distribution for white noise. To evaluate whether this value can be used to reject or not reject the null hypothesis, we calculated the distribution of K-S statistic values of EDFs drawn from the expected exponential distribution as follows.

1) First, we randomly draw 167 values uniformly distributed between 0 and 1 (sim_arr_). Here, 167 refers to the total number of LSP continuum values between 1.5 days and 100 days excluding bins near 8.5 days.

2) Then, we evaluate the expression −log_10_(1 − sim_arr_) to give a simulated set of values that follow the expected 1-*e*^−*z*^ distribution. Combined with the above step, this procedure is sometimes referred to as the inverse sampling technique.

3) We then compute the EDF of this simulated set of values drawn from 1-*e*^−*z*^ distribution.

4) Finally, we estimate the K-S statistic of this simulated set of values using its EDF.

The above steps are repeated 100,000 times to get a distribution of the K-S test statistic values for a given sample size of 167. This is shown as an orange histogram in fig. S9C. ASASSN-20qc’s observed K-S test statistic (dashed vertical red line), which is a measure of maximum deviation between the observed EDF and the theoretical cumulative distribution function (CDF), is within 1σ deviation of the distribution. This indicates that the null hypothesis cannot be rejected even at the 90% confidence level and suggests that LSP powers in the continuum are consistent with the expected exponential distribution, i.e., the LSP is consistent with being white between 1.5 days and 100 days.

To ensure that the above conclusion is not dependent on the choice of the statistic used, we also computed the Anderson-Darling statistic. Similar to above, we computed its distribution using bootstrap simulations (fig. S9D). Again, it is evident that the statistic computed from ASASSN-20qc’s observed LSP (vertical dashed red line) is consistent with the expected exponential distribution.

On the basis of the above tests, we concluded that the ODR LSP values are consistent with white noise and proceeded to measure the global statistical significance based on this noise model.

#### Monte Carlo simulations to estimate global statistical significance

The LSP power levels corresponding to the global 3 and 4σ values can be estimated using equation 18 of (*6*). These correspond to 11.1 and 14.8, respectively. The highest bin near 8.5 days is above the 4σ value with an adjacent/second highest bin crossing the 3σ value. This suggests that the quasi-periodicity is statistically significant at greater than at least the 4σ level.

However, because the signal we are trying to test is broad, i.e., over at least two frequency bins near 8.5 days, the standard approach of estimating significance based on just the highest bin will be inadequate. Because such an estimate will not include the contribution from multiple frequency bins, it will fail to capture the true significance estimate. By true significance, we mean an estimate that accounts for the fact that the signal is distributed in multiple frequency bins. Therefore, we devise a methodology that can account for multiple frequency bins. This approach is similar to (*113*) with the additional complexity of irregular sampling. The mains steps are as follows.

1) After establishing that the ODR curve’s variability is white, i.e., frequency-independent noise, we simulate a uniformly sampled white noise light curve using the algorithm of (*113*). The time resolution and temporal baseline of this light curve are 10 s and 150 days, respectively.2) Next, we sample this light curve exactly as the window function of the real data.

3) We then extract an LSP of these data and identify the frequency bin with the highest power value.

4) The LSP is normalized by the mean of all power values excluding those near the period corresponding to the maximum value in the LSP.

5) An array of sum of two neighboring LSP powers is generated from the normalized LSP from the step above. The maximum value of this array is saved.The above steps were repeated 500,000 times to get an array of 500,000 maximum LSP sums. From these measurements, we computed the probability to exceed a certain LSP sum value, i.e., 1-CDF. This is shown in Fig. 2C. The 3 and 4σ confidence levels are indicated. The peak in the LSP near 8.5 days found in NICER data of ASASSN-20qc is statistically significant at ≈2 × 10^−5^ level, which translates to *>*4.2σ equivalent for a normal distribution.

For completeness, we also extracted the energy-resolved light curves in the 0.3- to 0.55-keV and 0.75- to 1.0-keV band. These are shown in fig. S5.

Finally, we also tested the robustness of the peak in the LSP by changing the bandpass boundaries used in the definition of ODR by ∼20%. A significant peak near 8.5 days was present in all the tested cases.

### XSTAR energy table models

We calculated physically motivated XSPEC photoionization table models using the XSTAR code v. 2.39 (*114*). We produced a grid of photoionization models varying the column density and ionization parameter in a wide range of values of *N_H_* = 10^19^ to 10^23^ cm^−2^ and logξ(erg s^−1^ cm) = 0 to 4, respectively. We considered an input SED consistent with the data, that is, a blackbody continuum with temperature *T* = 10^6^ K (*E* = 0.09 keV) and a mean unabsorbed ionizing luminosity in the 1- to 1000-Ryd band (1 Ryd = 13.6 eV) of 1*.*5 × 10^44^ erg s^−1^, which is consistent with the observed narrow range between 1*.*2 × 10^44^ and 2 × 10^44^ erg s^−1^. We considered a constant density shell of 10^10^ cm^−3^, although the actual value of the density is not strictly important for a geometrically thin shell because the code would simply scale the distance to obtain the same ionization parameter [e.g., (*115*)]. All abundances were fixed to solar values. When modeled with an inverted Gaussian at *E* ≃ 800 eV, the width of the absorption feature is very large, σ*_E_* ≃ 70 eV (σ*_v_* ≃ 25*,*000 km s^−1^), so we tested XSTAR grids with increasing velocity broadening from 100 km s^−1^ upward, finding that the maximum velocity broadening of 30,000 km s^−1^ provides the best fit to the data. Such a high-velocity broadening is not physically interpreted as due to turbulence, but it is most likely indicating a rotation of the outflow launched close to the black hole [e.g., (*116*)]. A lower limit on the velocity broadening of *>*5000 km s^−1^ is derived considering the rotational velocity at a distance of *<*6000 *r_g_*, given by the light crossing time of the 8-day modulation of the outflow. A search for best-fit solutions was performed considering a wide range of redshifts for the XSTAR table, ranging from *z* = −0*.*4 to *z* = 0*.*1, to investigate the existence of rest frame to high-velocity outflows. The XSTAR tables self-consistently take into account all resonant lines and edges for a wide range of ionic species, from H up to Ni (*103*). The smoothness and lack of sharp edges clearly point to an interpretation of the absorption as due to a broadened and blueshifted OVIII Lyα transition, with a rest frame energy of 0.654 keV. We note that our photoionization modeling is consistent with the one adopted by (*85*) for the broad absorption feature in the XMM-Newton spectrum of the TDE ASASSN-14li.

To model possible low outflow velocity x-ray WA components in the high-energy resolution RGS data, we also calculated a separate XSTAR absorption table with a typical velocity broadening for WAs of 100 km s^−1^ (*117*).

The assumption of a single temperature black body is well justified as the difference in black body temperature is found to be within 15% with respect to the average among the different spectra (table S5). We quantitatively tested for a possible dependence of the estimated parameters of the outflow on the black body temperature performing a fit of the XMM#1 and NICER’s time-resolved spectra with a *kT* = 0*.*12 keV XSTAR table, corresponding to an extremely high temperature increase of 30%. We find that the outflow is always required and the best-fit values of the parameters are always consistent within the ≃2σ level independently of the considered black body temperature.

### ASASSN-20qc’s optical/UV evolution

After subtracting the host flux and correcting for foreground galactic extinction, we fit the Swift UVOT photometry as a blackbody using MCMC and forward-modeling methods to estimate the bolometric luminosity, temperature, and effective radius evolution of ASASSN-20qc. We obtained the Swift UVOT filter response functions from the Spanish Virtual Observatory Filter Profile Service. This approach is similar to the methods of several previous studies on TDEs and ANTs [e.g., (*17*, *55*, *118*)]. After obtaining the blackbody luminosity evolution, we estimated a bolometric light curve by scaling the ASAS-SN *g*-band light curve to match the bolometric luminosity evolution from the blackbody fits. Where there were no Swift data, we assumed a constant scaling with time (i.e., a flat temperature evolution; see fig. S12).

## Supplementary Materials

### This PDF file includes:

Sections S1 to S4 Figs. S1 to S16 Tables S1 to S7 Legend for movie S1 References
