# Supplementary material for: A case for a binary black hole system revealed via quasi-periodic outflows
Source: Sci Adv. 2024 Mar 27;10(13):eadj8898. doi: 10.1126/sciadv.adj8898 (PMC10971442; doi:10.1126/sciadv.adj8898)
Supplement: Supplementary file 1 — Sections S1 to S4 Figs. S1 to S16 Tables S1 to S7 Legend for movie S1 References [file sciadv.adj8898_sm.pdf]

Science Advances
AAAS

Supplementary Materials for

A case for a binary black hole system revealed via quasi-periodic outflows

Dheeraj R. Pasham *et al.*

Corresponding author: Dheeraj R. Pasham, dheeraj@space.mit.edu

Sci. Adv. **10**, eadj8898 (2024)
DOI: 10.1126/sciadv.adj8898

The PDF file includes:

Sections S1 to S4
Tables S1 to S7
Figs. S1 to S16
Legend for movie S1
References

Other Supplementary Material for this manuscript includes the following:

Movie S1

1 Summary of alternative models

We considered several models to interpret the above quasi-periodic variations. First, we consider a scenario consisting of a precessing accretion disk. This can, in principle, produce a quasi-periodicity for a certain black hole spin, outer radius of the precessing accretion disk, and a disk surface-density profile (*131*). However, a precessing disk should also result in a strong modulation of the continuum flux. In order to test this possibility, we calculated the Lomb-Scargle Periodogram of the continuum emission in the 0.3–0.55 keV energy band. As shown in Fig. S5b it is dominated by red noise and does not show statistically significant peaks, contrary to the clear peak at $\simeq$8.5 days in the outflow band (0.75-1.00 keV) shown in Fig. 2. We note that this result is not affected by a limited signal-to-noise, because the countrate in the continuum energy band is actually much higher than in the outflow band. The fact that there is no significant modulation in the continuum band strongly disfavor an interpretation as due to accretion disk precession.

There are also other lines of argument that disfavor a precessing disk with a persistent outflow. As can be seen from Fig. S10, going from the maxima to the minima, the outflow column density and the ionization parameter increase by about an order of magnitude, while the outflow velocity remains rather stable. This observed empirical pattern is inconsistent with a change of line of sight through any disk outflow model, which disfavors a disk outflow precession interpretation. Indeed, this statement is supported by three fundamental physical reasons: (i) if the outflow is radial, as expected in purely radiation-driven scenarios (e.g., (*121*)), we would expect no variability in the outflow parameters following a change in the line of sight; (ii) considering a conical geometry, as expected for line-driven outflows (*119, 120*), following an increase in column density, we would expect a decrease in ionization parameter and an increase in outflow velocity, because the line of sight would intercept the main outflow streamline; (iii) in the MHD-driven case (e.g., (*117,118*)), the disk outflow has a specific stratified structure depending on the radial distance and polar angle, which predicts that an increase in column density, when the line of sight is closer to the equatorial region, will be followed by a significant decrease of the ionization parameter and outflow velocity.

Another scenario one can envision is that of clumpy outflow with the individual clumps intervening our line of sight roughly once every 8.5 days. However, the chance probability of encountering such a uniformly clumpy configuration is <1 in a 50,000 as shown by Monte Carlo simulations above (see Methods section 7 and Fig. 2c) and hence unlikely to be the origin of the observed quasi-periodicity.

ASASSN-20qc's QPOut properties appear distinct from the phenomenon of quasi-periodic eruptions (QPEs) found in a small sample of four systems (*92,93,132*). Firstly, QPEs are intense soft X-ray bursts with amplitudes in the range of 10-200 (*92,132*). Such large amplitude changes in the soft X-ray flux are not seen here (see Fig. S5). Secondly, QPE spectra are thermal with no reported outflows similar to those seen here. A recent study found a warm absorber in the QPE source GSN 069 (*133*). However, they concluded that this warm absorber was stable with no discernible changes over an extended period between 2010 and 2021 (*133*) (see Methods

section 5.2).

The UFO is also detected in an *XMM-Newton* observation taken around MJD 59554, roughly a year after the start of *NICER* monitoring (see Fig. S11 and Table S4). The fact that the outflow is present even after the X-ray flux decreased by more than a factor of 200 at $\sim$0.01% Eddington limit, suggests that it is unlikely to be driven by radiation pressure. There are two possibilities for the origin of the outflow in the low-luminosity state: (i) if the perturber-induced scenario described below is valid, the object is still expected to move around the SMBH after the decline of the accretion rate due to depletion of the inner TDE accretion disc. Thus, the outflows are still being launched from the outer ADAF flow and their presence would be revealed via absorption of the X-rays from inner parts of the disk. Because we have only one observation after the outburst has ended, we cannot confirm the quasi-periodicity of the outflow in the low accretion rate regime. ii) The alternative is that this could be the persistent magnetized outflow seen in our simulations of ADAF accretion flows oriented face-on.

If you consider that the quasi-periodicity of the outflow during the outburst is due to the radiation-driven instability, this quasi-periodic outflow would have a different physical launching mechanism than the outflow seen after the outburst in the low-luminosity state. During the outburst, the outflow would originate from a small dense accretion disk with a relatively high accretion rate, while after the outburst, the outflow would originate from a magnetized, dilute, and much hotter ADAF accretion flow. Therefore, we would expect different parameters of the outflow in the two regimes, including the column density and the velocity. Because the measured properties of the outflow after the outburst are within the error bars more or less similar to the outflow during the outburst (taking into account that lower flux in the low-luminosity state means higher uncertainties and that we do not know, in which phase (ODR maxima or minima) the low-state measurement was taken), this is an argument against the radiation-driven instability origin for QPOuts unless an instability can operate over several orders of magnitude change in luminosity. Moreover, to achieve such a short period with the radiation pressure instability, the properties of the disc, including its size, would have to be quite fine-tuned, with a very small radial extent of the disk (*128*), which is supposed to radiate in the soft X-rays. In that case, however, the changes of the disk in the instability cycle would manifest as quasi-periodic variability of the inflow band, which is not seen for ASASSN-20qc. The radiation-driven origin of the periodicity is thus unlikely.

High-resolution 3D GRMHD simulations of magnetically arrested disks (MADs) have suggested the possibility of variable outflows of heated plasma due to magnetic reconnection near the event horizon (*123*). However, the long-term evolution of such MAD outflows, i.e., beyond a few cycles, is unclear at present due to computationally-limited integration time. Nevertheless, episodic outflows in MAD cycles based on longer time integration (of the order of tens of cycles) at lower resolutions, show a stochastic distribution of eruptions rather than a periodicity (see the blue curve in Figure 1 of (*134*)). Furthermore, MADs are estimated to produce pronounced variability in the continuum which is not seen in ASASSN-20qc (Fig. S5).

Concerning the Changing-look classification, typical traces of the changing-look phenomenon are not found during the observational campaign, i.e. the disappearance/reappearance

of broad optical lines associated with the AGN type shift (e.g., from type 1 to type 2, and vice versa). The UV/optical continuum variability is rather typical of TDE power-law decay. However, we cannot exclude that the source indeed transitioned from a certain AGN optical type to a type 1 AGN before and after the outburst. During the observational campaign, after the outburst, the optical spectra are rather constant and they are not changing on timescales of a week, as instead clearly seen for the X-ray absorption, indicating that the AGN is not undergoing recurrent optical changing-look events. In any case, the possibility that the source went through an optical changing-look event before and after the outburst does not change the conclusions derived from its X-ray spectrum. On the other hand, the X-ray monitoring shows a quasi-periodic variability in its absorbed spectrum which could be classified as a changing-look behavior, in the sense that the source oscillates between highly- and lowly-absorbed states with a timescale of 8.5 days. The physical origin of this behavior would still be consistent with our theoretical interpretation of an orbiting object repeatedly perturbing the SMBH accretion disk.

2 Perturber-induced outflow scenario

We investigate whether ASASSN-20qc 's observed outflow quasi-periodicity of $P_{obs} \approx 8.5$ days can be induced by repetitive perturber—SMBH disk interactions (*8*). The gravitationally bound perturber can either be a stellar object with an outflow (mass-losing star or a pulsar) or a black hole. This is motivated by the fact that for both early- and late-type galaxies with stellar mass approximately equal to that of ASASSN-20qc 's host ($\log(M_{\rm host}/M_{\odot})$ = $10.13^{+0.02}_{-0.01}$, see Section 4), the occurrence rate of nuclear star clusters (NSCs) is high, between 60-80% (*135*). NSCs can be associated with stellar power-law density cusps around the SMBH (*136, 137*) which can result in a fraction of NSC objects (stars/compact objects) on tightly bound orbits around SMBHs in a manner similar to Sgr A*'s S stars (*138, 139*).

Apart from in-situ star-formation, another way to build-up NSCs is the infall of massive star-forming or globular clusters which can also host intermediate-mass black holes (IMBHs; mass range of 10^{2-5} $M_{\odot}$). These black holes can inspiral towards the central SMBH after their cluster gets disrupted by tides (*140, 141*), and it is predicted to result in SMBH-IMBH comoving merger rates of $\sim 10^{-5}$ - 3×10^{-4} Gpc^{-3} yr^{-1} in the local Universe (*130*). IMBHs with masses $\sim 10^{3-4}$ $M_{\odot}$ can also be retained in NSCs as a result of repeated stellar mass black hole–stellar-mass black hole or stellar-mass black hole–star mergers (*142, 143*).

A perturber (star or a compact object) is associated with its influence radius. This size is typically larger than the effective stellar radius in the case of a star or a compact remnant with a hard surface (neutron star or a white dwarf), and is larger than the event horizon in the case of a black hole. Theoretical work by (*8*) has shown that if the influence radius is large enough, the perturber can modulate both the inflow and the outflow rates with a characteristic periodicity. Under the "unified model" of AGN (*144*), the presence of broad optical emission lines suggests that we are viewing ASASSN-20qc close to the SMBH's rotational axis. In the repetitive perturber—disk interactions scenario two outflows are ejected per orbit (*8*). However, because

of the orientation only one outflow per orbit is expected to lead to an observable absorption event along our light of sight. The other outflow will not cause an absorption event, will be Doppler de-boosted, and will likely be obscured by the accretion disk. Therefore, in the following analysis, we set the rest-frame perturber orbital period equal to the QPOut period, i.e., $P_{orb} = P_{obs}/(1+z) \approx 8.5/(1+0.056) \simeq 8.05$ days. Consistent with the estimates in section 2, we consider a SMBH mass range of 10^{7-8} $M_{\odot}$.

Given this SMBH mass range and $P_{\rm orb}$, the characteristic radius of the perturber orbit is $r_{\rm per} = [P_{\rm orb}c^3/(2\pi G M_\bullet)]^{2/3} r_{\rm g} \sim 37\,(10^8\,M_\odot) - 172\,(10^7\,M_\odot)\,r_{\rm g}$ (in gravitational radii or M assuming a zero SMBH spin; we use $r_{\rm g}$ or M units interchangeably in derivations or plots). For the intermediate value of the supermassive black hole mass of $M_\bullet = 10^{7.4}\,M_\odot$, which we adopt for most GRMHD simulations, the orbital distance of the perturber changes only slightly for the theoretically possible maximum range of SMBH spins – from $r_{\rm per} = 92.90\,r_{\rm g}$ for the maximum prograde spin, through $r_{\rm per} = 92.97\,r_{\rm g}$ for a zero spin, up to $r_{\rm per} = 93.04\,r_{\rm g}$ for the maximum retrograde spin. For the same SMBH mass range, this characteristic orbital radius does not exclude an orbiting, perturbing object, since the tidal disruption radius for a Solar-type star is,

$$r_{\rm t} \simeq R_\star \left(\frac{M_\bullet}{m_{\rm per}}\right)^{1/3} \sim 4.37 \left(\frac{R_\star}{1\,R_\odot}\right)\left(\frac{M_\bullet}{10^{7.4}\,M_\odot}\right)^{\frac{1}{3}}\left(\frac{m_{\rm per}}{1\,M_\odot}\right)^{-\frac{1}{3}} r_{\rm g}\,, \tag{S1}$$

where $R_\star$ is the stellar radius and $m_{\rm per}$ is the stellar (perturber) mass. The tidal disruption radius given by Eq. (S1) shows that essentially only red giants with stellar radii of a few$\times 10\,R_\odot$ would be partially disrupted. If one calculates the ratio of the tidal disruption radius to the stellar orbital distance, it is of the order of unity and less for the stellar radius of

$$R_\star \lesssim 16.9 \left(\frac{P_{\rm orb}}{8.05\,{\rm d}}\right)^{2/3}\left(\frac{m_{\rm per}}{1\,M_\odot}\right)^{1/3} R_\odot\,, \tag{S2}$$

regardless of the SMBH mass. In other words, stars that satisfy Eq. (S2) are always beyond the tidal radius and are not disrupted. On the other hand, the GRMHD study of (*8*) indicates that the perturbers with influence radii

$$\mathcal{R} \gtrsim 1\,r_{\rm g} = 53.2 \left(\frac{M_\bullet}{10^{7.4}\,M_\odot}\right) R_\odot \tag{S3}$$

are necessary to induce significant perturbations (see SI sections 3 and 4) This is only possible for large red giants and asymptotic giant-branch stars, whose envelopes would be tidally disrupted, and thus the remnant cores would become too small on the orbital timescale to induce significant perturbations. The other possibility are stars with powerful stellar outflows, which are rather unlikely as we show in subsequent sections. The restrictions for stars are less severe for smaller SMBH masses, in particular for $M_\bullet \lesssim 10^{6.9}\,M_\odot$, when $\mathcal{R} \lesssim 16.8\,R_\odot$, and hence the stars around such a SMBH would have large enough relative cross-sections of $\sim 1\,r_{\rm g}$ and would not be tidally disrupted.

The necessity for having a perturber with the influence radius $\mathcal{R} \sim 1\,r_{\rm g}$ can also be inferred from the column density of the enhanced absorption due to QPOuts. If we consider the column density of the absorbing material at the ODR minima, $N_{\rm h} \approx 1.5 \times 10^{22}\,{\rm cm}^{-2}$ (see Fig. S10), one can estimate the typical line-of-sight length-scale of absorbing gas clumps as $h \approx f_{\rm g} N_{\rm h}/n_{\rm flow}$, where $n_{\rm flow}$ is the number density of the accretion flow that is perturbed and from which the blob is ejected and $f_{\rm g}$ is the geometrical factor related to the perturber orbital inclination. If we consider the SMBH mass of $M_{\bullet} = 10^{7.4}\,M_{\odot}$ and the distance $r \approx 93\,r_{\rm g}$ associated with the source-frame period $P_{\rm orb} \approx 8.05$ days, then the mean ADAF electron number density is $n_{\rm flow} \approx 6.3 \times 10^{19} \alpha^{-1} (M_{\bullet}/M_{\odot})^{-1} \dot{m}\,[r_{\rm per}/(2 r_{\rm g})]^{-3/2}\,{\rm cm}^{-3} \approx 3.95 \times 10^{9}\,{\rm cm}^{-3}$ for the Eddington ratio of $\dot{m} = 0.05$ (*14*). This gives the length-scale of the ejected blobs in terms of the gravitational radius

$$\frac{h}{r_{\rm g}} \approx 1.02 f_{\rm g} \left(\frac{N_{\rm h}}{1.5 \times 10^{22}\,{\rm cm}^{-2}}\right) \left(\frac{\alpha}{0.1}\right) \left(\frac{\dot{m}}{0.05}\right)^{-1} \left(\frac{r_{\rm per}}{93\,r_{\rm g}}\right)^{3/2}. \tag{S4}$$

Under the assumption that the radius of influence of the perturbing body should be of the order of h in order to eject the absorbing material of a comparable size, this estimate indicates the necessity for $\mathcal{R}$ to be at least of the order of a gravitational radius.

Given the difficulties to have such large stellar perturbers (see the previous estimates with stellar radii), it is more likely that the perturber is of a compact nature. The mass range can be estimated from the Hill and Bondi radii as follows,

$$\begin{aligned} m_{\rm per,Hill} &= \frac{12\pi^2 G^2}{c^6} \frac{M_{\bullet}^3}{P_{\rm orb}^2} \\ &\simeq 94 \left(\frac{M_{\bullet}}{10^{7.4}\,M_{\odot}}\right)^3 \left(\frac{P_{\rm orb}}{8.05\,{\rm days}}\right)^{-2} M_{\odot}\,, \end{aligned} \tag{S5}$$

$$\begin{aligned} m_{\rm per,Bondi} &= \frac{G^{2/3}(4\pi^2)^{1/3}}{c^2} M_{\bullet}^{5/3} P_{\rm orb}^{-2/3} \\ &\simeq 2.7 \times 10^5 \left(\frac{M_{\bullet}}{10^{7.4}\,M_{\odot}}\right)^{5/3} \left(\frac{P_{\rm orb}}{8.05\,{\rm days}}\right)^{-2/3} M_{\odot}\,, \end{aligned} \tag{S6}$$

hence the expected mass range of the compact body is $m_{\rm per} \sim 10^2 - 10^5\,M_{\odot}$ or an intermediate-mass black hole (IMBH). The perturber can launch ultrafast outflows with the required column density, while at the same time, the perturbations of the X-ray continuum due to perturber-disk interactions and shocks are rather weak.

When we adopt the model set-up of (*145*) with the perturber passing through a standard disk formed following the TDE, the flares due to ejected shocked gas blobs are typically at least an order of magnitude below the observed X-ray flux (0.3-1.1 keV) of ASASSN-20qc. Adopting the X-ray luminosity during the enhanced accretion period, $L_X \sim 5 \times 10^{43}\,{\rm erg\,s^{-1}}$, the Eddington ratio is $\dot{m} \sim \kappa_{\rm bol} L_X / L_{\rm Edd} \sim 0.1$, hence similar to the one assumed by (*145*) for their standard-disk model. When we consider an interacting IMBH of $m_{\rm per} \sim 10^4\,M_{\odot}$,

whose characteristic radius is given by the Bondi radius, the X-ray flare luminosity due to the expanding shocked ejecta can be estimated as (relation A3 in (*145*)),

$$L_{\rm flare,IMBH} \lesssim 1.4\times10^{42}\left(\frac{\dot{m}}{0.1}\right)^{1/3}\left(\frac{M_\bullet}{10^{7.4}\,M_\odot}\right)^{5/9}\left(\frac{m_{\rm per}}{10^4\,M_\odot}\right)^{2/3}\left(\frac{P_{\rm QPOut}}{8.05\,{\rm days}}\right)^{-2/9}\,{\rm erg\,s^{-1}}\,, \tag{S7}$$

where we assumed that the IMBH interacts with the standard disk twice per orbit with the period set by quasiperiodic ultrafast outflows. Therefore, Eq. (S7) can be treated as an upper limit and the flare luminosity will be weaker for a more diluted ADAF flow expected for ASASSN-20qc at the distance where the IMBH orbits the SMBH. For the same perturber mass of $10^4\,M_\odot$ as well as the relative accretion rate, the plasmoid model by (*146*) predicts the flare luminosity of $L_{\rm flare,0.3-1.1keV} \lesssim 5.3\times10^{42}\,{\rm erg\,s^{-1}}$ in the $0.3-1.1$ keV band, assuming the blackbody emission at $\sim 10^6$ K. Hence, in both models, the shock emission is at least an order of magnitude below the quiescent level. Since $L_{\rm flare,IMBH} < L_X$ by an order of magnitude, the modulation of the X-ray continuum flux is negligible. A stellar perturber of $m_{\rm per} \sim 1\,M_\odot$ interacting with the standard disk provides even smaller shock-generated X-ray flux of (relation 17 in (*145*))

$$L_{\rm flare,star} \lesssim 6.7\times10^{41}\left(\frac{R_\star}{1\,R_\odot}\right)^{2/3}\left(\frac{M_\bullet}{10^{7.4}\,M_\odot}\right)\left(\frac{\dot{m}}{0.1}\right)^{1/3}\left(\frac{P_{\rm QPOut}}{8.05\,{\rm days}}\right)^{-2/3}\,{\rm erg\,s^{-1}}\,. \tag{S8}$$

Hence, the perturber-accretion disk shocks in ASASSN-20qc can generate only weak X-ray flares, generally below the quiescent X-ray level, mainly due to a relatively wide orbit of the perturber with the recurrence period of $\sim$ 8 days. During interactions with the more diluted ADAF, the shock-induced X-ray flux will be even smaller. Hence, a massive compact perturber, such as an IMBH, orbiting at $\sim 100\,r_{\rm g}$ can naturally account for the generation of periodic ultrafast outflows with a high enough column density to cause absorption, while the continuum X-ray emission is not affected significantly by the passages.

3 GRMHD simulations of the perturbed accretion flow

As in (*8*), we model the perturber-accretion disk interactions using the global general relativistic magneto-hydrodynamical (GRMHD) 2D and 3D simulations using the code `HARMPI` code (*10, 11*) that is based on the original `HARM` code (*12, 13*). The code with some of our applied modifications solves the equations of ideal magnetohydrodynamics on the curved Kerr spacetime background. The details on the numerical scheme can be found in (*8*). The gas is polytropic with an index of $13/9$, i.e., a value between the relativistic and the non-relativistic case to capture both relativistic electrons and non-relativistic protons.

This code proves to be efficient for simulating our scenario, in which the perturber comes very close to the central SMBH, hence the relativistic regime is essential, while the amount of accreted matter is negligible compared to the mass of the SMBH, therefore the assumption of fixed background described by Kerr metric is well justified. Because studying the repetitive

transits of the star requires long integration times of the simulations, computational efficiency is desirable. Our version of the code uses non-uniform spacing of the grid, which allows us to use a single grid without mesh refinements and still resolve well the closest neighborhood of the black hole while having sufficiently large grid to capture the whole accreting torus.

At the moment, we do not consider radiative transfer and associated feedback, hence the accretion flow does not cool radiatively. Therefore, the set-up is the most suitable for ADAF-type (Advection Dominated Accretion Flow) hot flows, i.e. SMBHs that accrete significantly below the Eddington limit. This seems to be the case of ASASSN-20qc, for which the integrated intrinsic luminosity in the range 1 eV–10 keV near the peak is $L_{\rm peak} = 10^{44.2}\,{\rm erg\,s^{-1}}$. For a black hole mass in the range $M_{\bullet} = 10^7 - 10^8\,M_{\odot}$, we obtain the Eddington ratio of $\lambda_{\rm Edd} = \kappa_{\rm bol}L_{\rm peak}/L_{\rm Edd} \sim 0.01 - 0.1$, where we consider the bolometric correction factor $\kappa_{\rm bol} \sim 1$. The Eddington ratio was clearly lower, $\lambda_{\rm Edd} \sim 10^{-5}$, before the detection of the optical/X-ray outburst, which implies a low-luminosity ADAF regime. On the other hand, the detected broad lines indicate the existence of an outer optically thick, standard disk (*147*). Indeed, a hybrid accretion disk solution with an inner ADAF-type flow and an outer standard thin disk is often suggested as a phenomenological description of the emission for low-luminosity AGN (see, e.g., (*99*)).

In that case, we expect–that prior to the outburst–the inner part of the accretion disk should be geometrically thick and hot, i.e. ADAF-like optically thin solution, with the transition given by the ADAF principle, i.e. whenever the ADAF solution is permitted, the accretion transitions into the hot flow, (*148–150*),

$$R_{\rm ADAF} = 1600\,\alpha_{0.1}^{4}\dot{m}_{0.05}^{-2} r_{\rm g}\,, \tag{S9}$$

where $\dot{m}_{0.05}$ is a dimensionless accretion rate expressed in Eddington units, $\dot{m} \equiv \dot{M}_{\bullet}/\dot{M}_{\rm Edd}$ and is scaled to 0.05 intermediate between $\lambda_{\rm Edd} \sim 0.01 - 0.1$ estimated above. The parameter α is a viscous parameter scaled to 0.1. Another possibility of the ADAF formation is via the evaporation due to the electron conduction between the cold disk and the hot two-temperature corona. In that case, the transition radius would be smaller but still above the distance scale where we assume the star-disk interactions (*147*),

$$R_{\rm evap} = 191\,\alpha_{0.1}^{0.8}\beta^{-1.08}\dot{m}_{0.05}^{-0.53}\,r_{\rm g}\,, \tag{S10}$$

where $\beta = P_{\rm g}/(P_{\rm g}+P_{\rm m})$ is the magnetization parameter defined as the ratio of the gas pressure to the total pressure. For a negligible magnetic field $\beta = 1$ while for large magnetic field, $\beta\ < 1$, which leads to a larger scale of the ADAF.

The initial condition for our simulations is given by the solution of a thick torus (*151*) yielding a large torus stretching between $r = 20\,r_{\rm g}$ to $r = 500\,r_{\rm g}$, which serves as a reservoir of matter for accretion. After the initial transient time $t_{\rm in}$, the perturber is added into the evolved state of the torus.

The pertuber moves along a geodesic trajectory that is calculated using the 4th-order Runge-Kutta scheme simultaneously with the evolution of the gas using the time-step found via the

adaptive GRMHD solver. This ensures a sufficient precision of the stellar position and the velocity within the evolving gaseous environment around the SMBH. The interaction of the perturber with the polytropic gas is modelled using a sphere of influence around the object with the radius $\mathcal{R}$ that mimics the stagnation radius around a wind-blowing or a magnetized star and/or the Hill radius or the synchronization radius around a dormant compact perturber (stellar or intermediate-mass black hole). As the perturber moves along its orbit, the grid cells that lie inside the sphere of influence adopt a velocity field comoving with the object, while other MHD parameters of the gas are kept intact. This effectively captures the perturber-gas interaction around the SMBH, in particular the development of a bow shock due to a supersonic motion and the propagation of density waves, while the internal properties of the perturbing object, in particular stellar evolution, are neglected.

We performed several different runs with parameters tailored specifically to ASASSN-20qc (see Table S7). The parameters differ in terms of the initial magnetic field configuration (one loop – the disk is prone to develop the Magnetically Arrested disk or MAD in short, more loops yield a stable accretion and outflow rate, so-called Standard and Normal Evolution – SANE), the distance of the perturber from the SMBH and its influence radius $\mathcal{R}$, and the inclination of its orbit. For all the runs, the stellar orbit is inclined with respect to the equatorial plane of the flow so that it effectively reaches the torus/funnel boundary, which leads to the blob ejection and the subsequent acceleration along this boundary. Since we do not have an estimate of the SMBH spin for ASASSN-20qc, we adopted a fiducial value of $a \simeq 0.4$, i.e., the black hole rotates only mildly. The SMBH mass of $10^{7.4}\,M_{\odot}$ was adopted for most runs in accordance with the observationally inferred mean value, while the larger value of $10^{7.95}\,M_{\odot}$ reflects the uncertainty and was chosen so that the orbital period of 8.05 days corresponds to the orbital distance of $40\,r_{\rm g}$.

We calculate the inflow rate $\dot{M}$ as well as the outflow rate $\dot{M}_{\rm out}$, which is split into the "upper" and the "lower" funnels. In particular, we integrate the outflow through the sector of the sphere along the symmetry axis with the opening angle of 45° pointing "upwards" and "downwards" with a radius of $r_{\rm diag} = 300\,r_{\rm g}$. To exclude the motion of the dense but slow gas inside the accreting torus, we integrate only the material which moves away from the black hole faster than a chosen threshold – its Lorentz factor satisfies the sequence of conditions – $\Gamma > 1.005$ ($v > 0.1c$), $\Gamma > 1.02$ ($v > 0.2c$), $\Gamma > 1.05$ ($v > 0.3c$), $\Gamma > 1.091$ ($v > 0.4c$) or $\Gamma > 1.155$ ($v > 0.5c$). In configurations with different spin and magnetic field strength and geometry and orbital parameters, the outflow achieves different velocities; however, overall the simulation outcome is only weakly dependent on the SMBH spin value.

In Fig. 3, we show a snapshot of Run 14 with the density distribution, the Lorentz factor of the outflow, and the outflow-rate distribution for $v > 0.2c$. In the panel d), we also show the temporal evolution of the inflow and the outflow rates. The outflow periodicity is clearly visible, while the inflow rate is a combination of the stochastic red-noise variability and the periodic effect of the perturber. We can notice one outflowing blob induced by the perturber interactions with the disk in panel c), which shows the spatial distribution of the mass-outflow rate and the bow shock formed by the motion of the perturber in the flow in panel a). The blobs

are moving along the boundary between the dense accretion torus and the magnetized funnel, which half-opening angle is approximately 30° depending on the parameters of the gas and the spacetime.

The trajectory of the perturber, in this case, is chosen to be mildly eccentric (with $e = 0.5$). This causes the shift in the position of the individual peaks in the outflowing rate. Due to the launching mechanism in our scenario, the timing of the individual expelled blobs is given by the times, when the perturber flies out from the disk and pushes the gas into the funnel. This roughly corresponds to the maxima/minima of angle θ. The frequency of oscillations in r and θ directions differs and, as the orbit undergoes the precession, there is the phase shift of those maxima/minima. Moreover, for such eccentric orbits, the radial distance of the perturber when achieving the turning point in θ angle differs, hence the blob is launched at different distances from the black hole. Therefore, the shape and amplitude of the individual peaks differ, depending on the gas condition at the place, where the perturber flies out from the disk.

Runs 1-8 and 12-13 yield similar results as the one presented here, with different timing of the peaks due to the eccentricity. The strength of the outflow varies with the size of the perturber as we discuss below.

Since the spin of the black hole is rather mild in our simulations, we can conclude that the fast rotation of the central black hole is not necessary for our scenario. The effect is viable for both the case of non-rotating, Schwarzshild SMBHs as well as for fast rotating Kerr SMBHs.

All the simulations presented so-far were computed in 2D, i.e., under the assumption of the axisymmetry of the system. However, the perturber surely breaks this symmetry while transiting through the flow within a small azimuthal angle. In ASASSN-20qc, the perturber is located quite far from the SMBH and its orbital period is long ($\sim 5630 r_{\rm g}/c \simeq 8.05$ days in the source frame), therefore it would be computationally very demanding to perform full 3D simulations with these parameters. However, to overcome this caveat, we perform simulations with the perturber located close to the SMBH, $r = 10\,r_{\rm g}, \mathcal{R} = 1\,r_{\rm g}$ with the same parameters both in 2D and 3D and compare the strength of the measured outflow rate. We normalize the strength of the outflow from the 2D simulation by a relation expressing the ratio of the azimuthal width of the perturbing body to the full angle.

$$\dot{M}_{\rm out} = \frac{2\mathcal{R}}{2\pi r_{\rm min}} F_n \dot{M}^{\rm 2D}_{\rm out}. \tag{S11}$$

The factor F_n is introduced due to the geometrical shape of the perturber and we fix its value to $F_n = 1/4$ by comparing with the outflow strength measured in the 3D simulation (Fig. S13). The case with $\mathcal{R} = 0.1\,r_{\rm g}$ is also shown and it yields the outflow rate smaller by more than three orders of magnitude. Hence, this demonstrates the strong dependence on the size of the perturber, which allows us to discuss its likely nature.

In case of larger eccentricity (Run 15 with $e = 0.67$), the perturber comes closer to the SMBH and has a larger impact on the flow with visible peaks in the accretion rate. However, we note that the influence on the accretion rate appears artificially enhanced in the 2D simulations in the manner as expressed by relation S11. We are aiming to study these quantitative effects

systematically with 3D simulations (follow-up work in progress).

For our last simulation, Run 16, we shifted the perturber closer to the SMBH, so that we can follow the evolution for a similar number of orbits (~ 15) as was observed in the case of ASSASN-20qc in 3D within a reasonable amount of computation time. The perturber with the influence radius $\mathcal{R} = 2M$ moves on a mildly elliptic ($e = 0.18$), inclined ($\iota = 67.7°$) orbit with pericenter $r_{\min} = 10M$ and apocenter $r_{\max} = 14.7M$. The characteristic periods, i.e. the inverse functions of the fundamental frequencies of geodesic motion in Kerr spacetime (*152*), in r, θ, and ϕ directions are $P_r = 370M$, $P_\theta = 273.3M$, $P_\phi = 269.7M$, respectively, while the total duration of the simulation was $4300M$.

In Fig. S14 we show the time dependence of the accretion rate and outflowing rate in two velocity bins $0.3c < v < 0.4c$ and $0.4c < v < 0.5c$. Even though the perturber is much closer to the SMBH with similar $\mathcal{R}$ as in our previous 2D simulations, there is no clear sign of periodicity in the periodogram of the inflow corresponding to either P_r, P_θ or P_ϕ, while the two peaks are visible in the periodogram of the outflow. It is possible, that with longer duration of the simulation, some signs of periodicity can emerge in the inflow rate, however, we are limited by computational resources at this point. However, the number of the covered cycles in this simulation is comparable to the observed one, hence constructing Lomb-Scargle periodograms from the longer runs would not correspond to the observational baseline.

It is reasonable to expect, that the effect of the perturber with the parameters derived for ASSASN-20qc on the accretion rate will be smaller than in this 3D case. This is because the flow is perturbed further away from the SMBH, there is more time for the density inhomogeneities to dissipate in the disc during their infall to the center. Therefore, we do not expect that the accretion rate in case of ASSASN-20qc would be significantly affected by the perturber.

4 On the nature of the orbiting perturber

The order of magnitude estimate of the influence radius of the perturber $\mathcal{R} \sim 1\,r_{\rm g}$ is supported by our GRMHD simulations. In Fig. S13, we show the GRMHD-based ratio between the mass outflow rate and the mass accretion rate in comparison with the measured one during the *NICER* Min phases (see Table S6). We plot the same quantity for four runs, which differ only by the influence radius $\mathcal{R}$, ranging from $\mathcal{R} = 2\,r_{\rm g}$ to $\mathcal{R} = 8\,r_{\rm g}$. While the largest perturber yields the outflow rate ten times greater than the inflow rate, the smallest perturber barely reaches the minimal outflow/inflow ratio during outflow peaks seen in observations $(\dot{M}_{out}/\dot{M}_{acc})_{\min} = 0.03$. The best agreement is achieved for $\mathcal{R} = 3\,r_{\rm g}$. Due to the uncertainties in other parameters of the system, such as the spin of SMBH or the properties of the magnetic field, this still serves only as an order of magnitude estimate.

Since we can constrain the perturber influence radius to $\mathcal{R} \approx 3\,r_{\rm g}$, we can infer the likely nature of the perturber from mechanisms that can create such a large influence radius. For a compact object without any outflow, the largest scale of gravitational influence of the perturber within the two-body problem is given by the tidal or the Hill radius. Because the whole gas

attracted by the perturber within the Hills sphere does not comove with the object, the Hills' relation gives us a lower limit on the mass of the perturber,

$$\begin{aligned} m_{\rm per,Hill} &= \frac{12\pi^2 G^2 M_\bullet^3}{P_{\rm orb}^2 c^6}\left(\frac{\mathcal{R}}{r_{\rm g}}\right)^3 \\ &= 2532\left(\frac{P_{\rm orb}}{8.05\,{\rm d}}\right)^{-2}\left(\frac{\mathcal{R}}{3\,r_{\rm g}}\right)^3\left(\frac{M_\bullet}{10^{7.4}\,M_\odot}\right)^3 M_\odot\,, \end{aligned} \tag{S12}$$

which is consistent with an intermediate-mass black hole of $\sim 10^2 - 10^5\,M_\odot$ given the uncertainty of $\mathcal{R}$ as well as that of the primary black hole mass in the range of $10^7 - 10^8\,M_\odot$.

The synchronization radius $R_{\rm sync}$ introduced by (*8*), and based on the gas drag force resulting from the Bondi-Hoyle-Lyttleton accretion (*153–155*), expresses the gas sphere that receives the full momentum from the star and starts comoving, which may not be entirely necessary for the ejection of larger blobs into the funnel region. In this regard, $R_{\rm sync}$ can be used to derive an upper limit for the perturber mass. Using the relation for the drag force acting on the IMBH for the interaction time Δt derived by (*155*), we can derive the synchronization radius from the conservation of momentum, $|F_{\rm df}|\Delta t \approx M_{\rm gas}v_{\rm rel} \sim (4/3)\pi R_{\rm syn}^3 \rho_{\rm gas} v_{\rm rel}$, where $v_{\rm rel} \approx (2GM_\bullet/r_{\rm per})^{1/2}$ is the relative velocity of the IMBH with respect to the accretion disk for the perturber distance $r_{\rm per}$ from the SMBH and considering high inclinations with respect to the disk equatorial plane. By setting $\Delta t \approx P_{\rm orb}/4$, which corresponds to the duration of the IMBH passages through a thick flow with the scale-height to radius ratio of the order of unity, we obtain,

$$\begin{aligned} \frac{R_{\rm syn}}{r_{\rm g}} &\approx \left(\frac{3I}{8\pi}\right)^{1/3}\frac{c^2}{\sqrt{2}}\frac{(Gm_{\rm per})^{2/3}}{(GM_\bullet)^{4/3}}P_{\rm orb}^{2/3}\,, \\ m_{\rm per,syn} &\approx 35\,671\left(\frac{P_{\rm orb}}{8.05\,{\rm d}}\right)^{-1}\left(\frac{\mathcal{R}}{3\,r_{\rm g}}\right)^{3/2}\left(\frac{M_\bullet}{10^{7.4}\,M_\odot}\right)^2 M_\odot\,, \end{aligned} \tag{S13}$$

where the factor $I = \ln(r_{\rm max}/r_{\rm min})$ and $r_{\rm max}$ and $r_{\rm min}$ correspond to the sizes of the surrounding gaseous medium and the perturbing object, respectively. For the supersonic motion of the perturber through the accretion disk on a highly inclined orbit, the factor I can be approximated as $I \approx \ln(M_\bullet/m_{\rm per}) \sim 7-9$ (*156*), which we approximate by setting $I \sim 10$ for further estimates.

The lower and upper mass limits given by the Hill and the synchronization radii, Eqs. (S12) and (S13) respectively, give a broad range of masses $\sim 10^3 - 10^5\,M_\odot$ consistent with the intermediate-mass as well as a low-mass supermassive black hole, hence a massive, non-stellar perturber. The uncertainty is mainly given by the primary black hole mass as well as the size of the influence radius. We plot the influence radii $\mathcal{R}$ as a function of the primary SMBH in Fig. S15 considering both the Hill radii as well as synchronization radii for limiting values of massive black-hole perturbers that still yield the influence radius of $\mathcal{R} = 3r_{\rm g}$ for the lower and the upper limit of the SMBH mass (10^7-$10^8\,M_\odot$). Considering the Hill-radius mass estimate,

we obtain the range of $m_{\rm per,Hill} = 160 - 1.6 \times 10^5\,M_{\odot}$, while the synchronization-radius relation gives a range of $m_{\rm per,syn} = 5.65 \times 10^3 - 5.65 \times 10^5\,M_{\odot}$. As it can be inferred from Fig. S15, there is an overlap in the mass range between $\sim 10^3$ and $\sim 10^5\,M_{\odot}$.

However, the presence of the second supermassive black hole is less likely due to the short merger timescale for such a system. If we consider the initial perturber distance of r_0 that corresponds to its orbital period of $P_{\rm orb} \sim 8.05$ days, the orbit is well in the weak field of the primary and the gravitational radiation-reaction is weak. The merger timescale can then be estimated from the leading post-Newtonian formula as follows (*157*)

$$\begin{aligned}\tau_{\rm merge} &= \frac{5c^5}{256G^3}\frac{r_0^4}{M_{\bullet}m_{\rm per}(M_{\bullet}+m_{\rm per})}\,,\\ &= \frac{5c^5}{(2^{32}G^5\pi^8)^{1/3}}\frac{(M_{\bullet}+m_{\rm per})^{1/3}}{M_{\bullet}m_{\rm per}}P_{\rm orb}^{8/3}\,. \end{aligned} \tag{S14}$$

For the lower SMBH-IMBH pair mass limit, we obtain $\tau_{\rm merge}(10^{7.4}\,M_{\odot}, 10^3\,M_{\odot}) \sim 143\,426$ years, while for the upper SMBH-IMBH pair mass limit, we get $\tau_{\rm merge}(10^{7.4}\,M_{\odot}, 10^5\,M_{\odot}) \sim 1436$ years. Hence, the perturbation by a more massive IMBH is less likely, as it would necessarily result in the short merger timescale of $\lesssim 1000$ years. In bottom panel of Fig. S13, we plot the merger timescale in years as a function of the perturber mass. Since it is more plausible to have a concurrent tidal disruption event, which occurs with the rate of $\sim 10^{-4}\,{\rm yr}^{-1}$ per galaxy, to take place in the binary system with the merger timescale at least $\sim 10^4$ years, this statistical argument favours the perturbers with the mass of $\lesssim 10^4\,M_{\odot}$. Specifically, for the primary SMBH mass of $M_{\bullet} = 10^7\,M_{\odot}$, we obtain $m_{\rm per} \lesssim 26500\,M_{\odot}$, for the intermediate value of $M_{\bullet} = 10^{7.4}\,M_{\odot}$ we get $m_{\rm per} \lesssim 14\,300\,M_{\odot}$, while for $M_{\bullet} = 10^8\,M_{\odot}$, we obtain $m_{\rm per} \lesssim 5700\,M_{\odot}$.

We also checked the signal-to-noise ratios for a possible detection by LISA in the 2030s (*158*). Since the gravitational radiation is weak for the SMBH-IMBH pair, the period of the binary will not evolve significantly in the next decade. Consequently, the source will be outside of the frequency range of LISA and the signal-to-noise ratios will be below 10^{-2} in all admissible scenarios for the primary and the secondary masses.

A star with an outflow – either a wind-blowing star or a pulsar whose stagnation radius can be analytically estimated (*8*) – appears to be a much less suitable candidate for the perturber due to a small size of the associated stagnation radius across a wide range of parameters. Considering the SMBH mass of $M_{\bullet} = 10^{7.4}\,M_{\odot}$ and assuming the hot-flow density profile for $\dot{m} \sim 0.05$ (*14*), we obtain the $R_{\rm wb}^{\rm ADAF} \sim 0.76 r_{\rm g}$ as the stagnation radius of the wind-driven shock for the mass-loss rate of $\dot{m}_{\rm w} = 10^{-3}\,{\rm M}_{\odot}\,{\rm yr}^{-1}$ and the terminal wind velocity of $v_{\rm w} = 10^3\,{\rm km\,s^{-1}}$, which are rather large, unlikely values. The same stellar-wind parameters yield four orders of magnitude smaller stagnation radius $R_{\rm wb}^{\rm thin} = 5.36 \times 10^{-5}\,r_{\rm g}$ for the case of a standard thin disk with the same accretion rate. For the larger black-hole mass of $M_{\bullet} = 10^8\,M_{\odot}$, we obtain $R_{\rm wb}^{\rm ADAF} \sim 0.12 r_{\rm g}$ and $R_{\rm wb}^{\rm thin} = 5.93 \times 10^{-6}\,r_{\rm g}$ for the same stellar-wind parameters. The stagnation radii of the order of $0.1\,r_{\rm g}$ are plausible for either young massive stars (Wolf-

Rayet stars) with fast winds or massive late-type stars (red supergiants) with large mass-loss rates. Hence, for a typical main-sequence Sun-like star, the stagnation radius is orders of magnitude below the gravitational radius. For the case of an orbiting young pulsar with the large spin-down energy of $\dot{E} = 10^{38}\,\mathrm{erg\,s^{-1}}$ comparable to the energetic Crab nebula pulsar, the stagnation radius of the pulsar wind bubble is $R_{\rm psr}^{\rm ADAF} = 1.7 \times 10^{-2}\,r_{\rm g}$ and $R_{\rm psr}^{\rm thin} = 1.2 \times 10^{-6}\,r_{\rm g}$ for $M_\bullet = 10^{7.4}\,M_\odot$ for the hot ADAF and the thin-disk solutions with $\dot{m} = 0.05$, respectively. For $M_\bullet = 10^{8}\,M_\odot$, we obtain $R_{\rm psr}^{\rm ADAF} = 2.8 \times 10^{-3}\,r_{\rm g}$ and $R_{\rm psr}^{\rm thin} = 1.4 \times 10^{-7}\,r_{\rm g}$ for the same spin-down energy.

An interesting possibility for the perturber would be a binary system with the semi-major axis of $a_{\rm bin} \sim 2\mathcal{R}$, i.e. the binary separation would correspond to the twice of the influence radius of the perturber. The semi-major axis of such a system is limited by the tidal field close to the SMBH. The binary needs to be located at the distance of $d_{\rm bin} \gtrsim r_{\rm T} \sim a_{\rm bin}(M_\bullet/m_{\rm bin})^{1/3}$, which puts an upper limit on the component separation at the distance $d_{\rm bin} = r_{\rm per}$ that corresponds to the orbital period of $P_{\rm orb}$,

$$
\begin{aligned}
a_{\rm bin} &\lesssim r_{\rm per}\left(\frac{m_{\rm bin}}{M_\bullet}\right)^{1/3} \\
\mathcal{R}_{\rm bin} &= \frac{1}{2}\frac{a_{\rm bin}}{r_{\rm g}} \lesssim \frac{c^2}{2G^{2/3}(4\pi^2)^{1/3}} P_{\rm orb}^{2/3} \frac{m_{\rm bin}^{1/3}}{M_\bullet} \\
&\simeq 0.34\left(\frac{P_{\rm orb}}{8.05\,{\rm d}}\right)^{2/3}\left(\frac{m_{\rm bin}}{10\,M_\odot}\right)^{1/3}\left(\frac{M_\bullet}{10^{7.4}\,M_\odot}\right)^{-1}\,. \qquad \text{(S15)}
\end{aligned}
$$

Hence, a binary consisting of two approximately equally massive main-sequence stars of $5\,M_\odot$ each would have to be compact with $\mathcal{R}_{\rm bin}$ an order of magnitude below the limit of $\mathcal{R} \sim 3r_{\rm g}$. For a larger $\mathcal{R}_{\rm bin}$, the binary would disrupt (Hills mechanism, (*159*)). The only possibility to have $\mathcal{R}_{\rm bin} \sim 3\,r_{\rm g}$ is for $m_{\rm bin} \gtrsim 6\,800\,M_\odot$, i.e. for two IMBHs (e.g. of nearly equal mass of $\sim 3400\,M_\odot$) that we already proposed. However, such a system of two IMBHs orbiting each other with the period of ~ 8.05 days (in 1:1 resonance with the binary orbital period around the SMBH) is less likely and it is not necessary to explain QPOuts. Moreover, the required high inclination of the SMBH-IMBH system is consistent with the IMBH receiving the recoiling velocity kick following the merger with another IMBH, see e.g. (*160*). The IMBH binary as such would be relatively long-lived with the merger timescale of ~ 20.4 Myr.

Another possible set-up is that an IMBH of $\simeq 10^4\,M_\odot$ would be orbited by a star of mass $m_\star$ within $a_{\rm bin} \lesssim 6.84 r_{\rm g}$ (here we consider the basic condition for the tidal stability), which corresponds to $\sim 1.69\,{\rm AU}$ or $\sim 364\,R_\odot$. Such a star would not be tidally disrupted by the IMBH up to the stellar radius of $\sim 16.9\,R_\odot$ (for the stellar mass of $1\,M_\odot$). In case the IMBH would be orbited by a larger star of $\gtrsim 10\,R_\odot$ at the distance of $\sim 1.69\,{\rm AU}$, such a binary system would disrupt as the IMBH descends further towards the SMBH due to gravitational-wave emission. Subsequently, the star is tidally disrupted by the SMBH. At the time of the IMBH-star separation due to the Hills mechanism, the maximum radius of the tidally stable star

around the IMBH is equal to the tidal radius of a star around the SMBH,

$$R_\star \sim P_{\rm orb}^{2/3}(Gm_\star/4\pi^2)^{1/3}\,,$$
$$\sim 16.9\left(\frac{P_{\rm orb}}{8.05\,{\rm d}}\right)^{2/3}\left(\frac{m_\star}{1\,M_\odot}\right)^{1/3}R_\odot\,, \tag{S16}$$

assuming that the IMBH-star binary and the separated components share approximately the same orbit with the orbital period of $P_{\rm orb}$. It is clear that conditions given by Eqs. (S16) and (S2) are the same. In this regard, the TDE and the IMBH-induced recurrent outflow could be causally connected. Although this seems to be just a hypothetical, fine-tuned scenario, considering the scenario of an infalling stellar cluster that hosts the IMBH at the center, it appears to be quite plausible as the necessary final outcome of the cluster dissolution when only a single IMBH-star binary remains. (*130*).

Given the uncertainty in ASASSN-20qc's SMBH mass, one can constrain the SMBH mass ranges where perturbers are more likely stars or black holes, using the condition that the influence radius needs to be relatively large to produce escaping blobs causing periodic absorbing events like those detected from ASASSN-20qc, i.e. $\mathcal{R} \simeq 3GM_\bullet/c^2$. In the left panel of Fig. S16, we plot the influence radius $\mathcal{R}$ expressed in Solar radii as a function of the SMBH mass. Furthermore, we include the upper limits on the stellar radius given by the tidal stability condition as well as the estimates of bow-shock radii for the ADAF and the standard disks (for $\dot{m} = 0.05$ and the stellar-wind parameters specified in the legend). Given the perturber distance around the SMBH corresponding to $P_{\rm orb} = 8.05$ days, there is a limiting SMBH mass of $M_\bullet \simeq 10^{6.27}\,M_\odot$, below which perturbers with $\mathcal{R} \sim 3\,r_{\rm g}$ can likely be stars, either due to their physical cross-section or wind bow shock. For heavier SMBHs, stellar-mass and intermediate-mass black holes will have a large enough influence radius due to their gravitational influence, see the estimates given by Hill and synchronization radii in Fig. S16 (based on Eqs. (S12) and (S13), respectively). In the same SMBH mass range, stars would need to have large physical radii of $\gtrsim 11.7\,R_\odot$, which decreases the likelihood considering the fact that the star is found at a special evolutionary stage (a late-type red giant), and more importantly, these stars would not be tidally stable at the required distance given by the QPOut (orbital) period. Hence, in case the SMBH mass for ASASSN-20qc is $\gtrsim 10^7\,M_\odot$, an IMBH is the only possibility for a stable perturber with a large-enough cross-section and, at the same time, a long enough merger timescale so that a TDE is likely to occur concurrently.

4.1 Orbital stability of the SMBH-IMBH system

The SMBH-IMBH system is relatively stable in terms of the secular orbital changes due to gravitational-wave emission. According to Eq. (S14), the gravitational-wave inspiral timescale is $\tau_{\rm merge} \sim 10\,246$ years for the circularized orbit of the IMBH of $14\,000\,M_\odot$ around the SMBH of $10^{7.4}\,M_\odot$ with the initial distance of $r_0 \simeq 93\,r_{\rm g}$. The influence radius $\mathcal{R}$ of the IMBH for this configuration is $5.3\,r_{\rm g}$ and $1.6\,r_{\rm g}$ as given by the Hill and the synchronization radii, respectively.

In other words, the timescale for a semi-major axis decrease by one gravitational radius is ~ 434 years. During the inspiral time of the IMBH, e.g. from 500 to 93 $r_{\rm g}$, which takes of the order of 8.56×10^{6} years, the orbit gets effectively circularized, e.g. starting with $e_0 = 0.9$ at $r_0 = 100\,r_{\rm g}$, it takes $\sim 156\,000$ years to reach $e = 0.01$ (*157*). However, a mildly eccentric orbit cannot be excluded at this point. A non-zero eccentricity can actually address a quasiperiodic nature of the outflow as indicated by the temporal evolution of the ODR, see Fig. 2, since the eccentric orbit undergoes a prograde relativistic (Schwarzschild) precession. This is supported by the GRMHD simulation runs with eccentric perturber orbits (see Fig. 3).

The IMBH orbiting the SMBH on a mildly eccentric orbit with the semi-major axis $a_{\rm per}$ and the eccentricity $e_{\rm per}$ is subject to the prograde, relativistic Schwarzschild precession of the argument of the pericenter. The orbital plane of the IMBH and hence the position angle of the outflow is not changed, but the properties of the outflow could be affected, i.e. the outflow launch radius and hence the outflow velocity could differ depending on whether the perturber-disk interaction takes place close to the pericenter or the apocenter of the eccentric orbit. The Schwarzschild precession timescale for the change of the argument of the pericenter by $180°$, i.e. when the absorbing blob launch radius would effectively be changed from the pericenter to the apocenter of the orbit, is,

$$\begin{aligned}\tau_{\rm S}(180°) &= \frac{c^2 a_{\rm per}(1-e_{\rm per}^2)P_{\rm orb}}{6GM_\bullet}\\ &= \frac{c^2(1-e_{\rm per}^2)P_{\rm orb}^{5/3}}{6(4\pi^2)^{1/3}(GM_\bullet)^{2/3}}\\ &\simeq 109.5\left(\frac{P_{\rm orb}}{8.05\,{\rm d}}\right)^{5/3}\left(\frac{1-e_{\rm per}^2}{0.88}\right)\left(\frac{M_\bullet}{10^{7.4}\,M_\odot}\right)^{-2/3}\ {\rm days},\end{aligned} \tag{S17}$$

where we scaled the eccentricity to $e_{\rm per} = 0.35$ corresponding to Run 3 in Table S7, hence $1-e_{\rm per}^2 \simeq 0.88$. The Schwarzschild precession timescale shows that the argument of pericenter can in principle rotate by $180°$ during the observational coverage of 12 QPOuts (~ 100 days in the rest frame).

The orbiting massive perturber with the orbital distance of 100 $r_{\rm g}$ also provides a sufficient directional stability for the duration of QPOuts (12 cycles of total duration ~ 100 days in the rest frame). This is a basic requirement since the ejected outflows need to cross the line of sight once per orbital period. In fact, for the IMBH perturber orbiting $10^{7.4}\,M_\odot$ SMBH at $93\,r_{\rm g}$, the Lense-Thirring precession timescale, which corresponds to the rotation of the longitude of line of nodes by $90°$, can be expressed as

$$\begin{aligned}t_{\rm LT}(90°) &= \frac{c^3 P_{\rm orb}^2}{16\pi G M_\bullet a}\\ &= 2255\left(\frac{P_{\rm orb}}{8.05\,{\rm d}}\right)^2\left(\frac{M_\bullet}{10^{7.4}\,M_\odot}\right)^{-1}\left(\frac{a}{0.4}\right)^{-1}\ {\rm days}\,,\end{aligned} \tag{S18}$$

where the spin parameter a is scaled to 0.4 and the orbital eccentricity is set to zero. For the favored spin of $a = 0.9$ given the inferred disk temperature of 0.085 keV (from analysis in Methods section 6), the $90°$ Lense-Thirring timescale gives an upper limit on the SMBH mass of $10^{8.4}\,M_{\odot}$, for which $t_{\rm LT} \sim 100$ days (i.e. 12 detected QPOuts). We show the distance dependencies of the Schwarzschild (for $e_{\rm per} = 0.35$) and Lense-Thirring precession (for $e_{\rm per} = 0$ and $a = 0.4$) timescales in Fig. S15 alongside other relevant dynamical timescales. The directional stability tends to disfavor the accretion-disk instability mechanisms, which are directionally stochastic, unless the instability would periodically launch a relativistic absorbing gas clump across a broad azimuthal range, i.e. an expanding ring-like blob, which is, however, unlikely.

With more X-ray data in the future, in particular of high-cadence where persistent low-outflow epochs can clearly be distinguished from enhanced-outflow epochs, the perturber model can further be tested for different orbital elements, in particular eccentricity, which results in different rates of Schwarzschild precession in the orbital plane. Therefore, it would modulate the exact timing of enhanced absorption events as well as the outflow velocity. In case the enhanced absorption events disappear for a certain period of time, it would be an indication of the precession of the line of nodes due to the Lense-Thirring effect, i.e. the perturber-induced outflow footpoint would essentially precess as well depending mostly on the SMBH spin.

4.2 Formation channels for IMBHs and IMBH-SMBH pair statistics

IMBHs can be formed via different channels, namely two basic formation mechanisms are (i) cosmological/primordial related to the direct collapse of gaseous clouds or remnants of population III stars (*161, 162*) and (ii) repeated stellar and black-hole collisions and the subsequent growth by accretion and/or mergers inside massive stellar clusters (runaway scenario); see (*81*) for a review. Channel (ii) can lead to an increased occurrence of IMBHs in NSCs with respect to the rest of host galaxies due to

- gradual build-up of NSCs via the infall of massive stellar clusters hosting IMBHs (*130, 140, 141, 163*),
- a series of mergers of stellar black holes (*143*) or stellar black holes with other stars in the NSC (*142*).

Thanks to the deeper gravitational potential of NSCs in comparison with other stellar environments, IMBHs as merger products can be retained and accumulated within galactic nuclei since the received post-merger recoil kicks are typically less than the required escape velocity from the NSC. Subsequently, they can form tight pairs with the SMBH as they descend within the SMBH sphere of influence on the dynamical friction timescale. The dynamical friction timescale is especially short for perturbers significantly more massive than field stars. In case the IMBH is moving at a comparable speed with respect to field stars at large distances from

the SMBH, i.e. $v_{\rm per} \sim \sigma_\star$, then the dynamical friction time can be estimated as

$$\begin{aligned} T_{\rm df} &= \frac{3}{8}\sqrt{\frac{2}{\pi}}\frac{\sigma_\star^3}{G^2\rho_\star m_{\rm per}\ln\Lambda} \\ &\approx 4\left(\frac{\sigma_\star}{135\,{\rm km\,s^{-1}}}\right)^3\left(\frac{\rho_\star}{5.8\times10^4\,M_\odot{\rm pc^{-3}}}\right)^{-1}\left(\frac{m_{\rm per}}{10^4\,M_\odot}\right)^{-1}\left(\frac{\ln\Lambda}{17}\right)^{-1}{\rm Myr}\,, \end{aligned} \quad \text{(S19)}$$

where $\sigma_\star \sim 135\,{\rm km\,s^{-1}}$ is the stellar velocity dispersion estimated from $M_\bullet$-$\sigma_\star$ relation (i.e. (*69*) for $M_\bullet = 10^{7.4}\,M_\odot$), $\rho_\star$ is the stellar mass density inside the sphere of the SMBH gravitational influence (considering $M_\star \sim 2\times10^{7.4}\,M_\odot$ inside the sphere of influence given by the velocity dispersion), and $\ln\Lambda$ is the Coulomb logarithm ($\ln\Lambda \sim \ln(M_\bullet/M_\odot) \sim 17$).

There are several ways to estimate potential number of SMBH-IMBH sources within the redshift of ASASSN-20qc, $z \lesssim 0.06$. Using the N-body model of the globular cluster disruptions within the NSC and subsequent interactions of the IMBHs with the SMBH, the SMBH-IMBH comoving merger rate estimate is $\Gamma_{\rm AC} = 0.03\,{\rm Gpc^{-3}\,yr^{-1}}$ (*164*) within the local Universe. This translates into $N_{\rm SMBH-IMBH} = \Gamma_{\rm AC}V_{\rm com}(<z)\tau_{\rm merge} \sim 20.4$ pairs for the comoving volume within $z = 0.06$ and their merger timescale of $\tau_{\rm merge} \sim 10^4$ years, during which a TDE is likely to occur. Within the semi-analytical framework of the disruptions of globular clusters hosting an IMBH, the SMBH-IMBH comoving merger rate is smaller than the previous estimate, $\Gamma_{\rm F} \sim 10^{-5} - 3\times10^{-4}\,{\rm Gpc^{-3}\,yr^{-1}}$ (*130*), where the range corresponds to the IMBH occupation fraction between 0.1 and 1.0 inside globular clusters, which gives $N_{\rm SMBH-IMBH} = \Gamma_{\rm F}V_{\rm com}(<z)\tau_{\rm merge} \sim 0.006 - 0.2$ pairs that are about to merge within 10^4 years (this could be still an upper limit for this model since there is no evidence for globular clusters hosting IMBHs; (*81*)). There is a wide range of the number of SMBH-IMBH pairs in the local Universe, however, they are all consistent within the uncertainties with at least one tight SMBH-IMBH pair with $\tau_{\rm merge} \sim 10^4$ years within the redshift of ASASSN0-20qc.

In addition, one can consider an estimate of the number of suitable SMBH-IMBH tight pairs based on the assumption that each NSC contains at least one IMBH within the sphere of influence of the SMBH. In that case the number of potentially detectable SMBH-IMBH tight pairs can be estimated as,

$$N_{\rm pair} \sim n_{\rm NSC}\nu_{\rm infall}\tau_{\rm merge}V_{\rm com}(<z)\,, \quad \text{(S20)}$$

where $n_{\rm NSC}$ is an approximate number density of galaxies hosting an NSC, $\nu_{\rm infall} \equiv 1/T_{\rm df}$ is the infall rate of IMBHs within an NSC as given by the dynamical friction timescale (see Eq. (S19)), $\tau_{\rm merge}$ is the SMBH-IMBH merger timescale (see Eq. (S14)), and $V_{\rm com}(<z)$ is the comoving volume within the redshift z. The number of galaxies per year that host a SMBH-IMBH pair and in which a TDE occurs can be expressed as follows

$$N_{\rm pair,TDE}[{\rm yr^{-1}}] = N_{\rm pair}\dot{N}_{\rm TDE}\,, \quad \text{(S21)}$$

where $\dot{N}_{\rm TDE}$ is a mean TDE rate per galaxy. These quantities can be estimated in the following way:

- $n_{\rm NSC} \sim 0.037\,{\rm Mpc}^{-3}$ by integrating the Schechter function

$$\Phi(M_{\star,{\rm gal}}) = \frac{\Phi_\star}{M_{\rm c}} \left(\frac{M_{\star,{\rm gal}}}{M_{\rm c}}\right)^{\alpha_{\rm c}} \exp\left(-\frac{M_{\star,{\rm gal}}}{M_{\rm c}}\right), \tag{S22}$$

  over the range of galactic stellar masses $M_{\star,{\rm gal}} = 10^8 - 10^{10}\,M_\odot$ (with $\Phi_\star = 0.84 \times 10^{-3}\,{\rm Mpc}^{-3}$, $M_{\rm c} = 10^{11.14}\,M_\odot$, and $\alpha_{\rm c} = -1.43$ fixed; see e.g. (*130*)), i.e. galaxies that have an NSC occupation fraction in the range $60\% - 80\%$ (*135*),

- $\nu_{\rm infall} = 1/T_{\rm df}$, where $T_{\rm df} \propto \sigma_\star^3/(\rho_\star m_{\rm per}) \sim M_\bullet^2/(\sigma_\star^3 m_{\rm per})$. Considering the range of SMBH masses, and related differences in the stellar velocity dispersion as well as in the SMBH influence radii, $T_{\rm df}(10^7\,M_\odot) \sim 5 \times 10^5$ years, $T_{\rm df}(10^{7.4}\,M_\odot) \sim 3 \times 10^6$ years, and $T_{\rm df}(10^8\,M_\odot) \sim 40 \times 10^6$ years for SMBHs of 10^7, $10^{7.4}$, and $10^8\,M_\odot$, respectively,

- $\tau_{\rm merge} \sim 10^4$ years, i.e. selection of SMBH-IMBH pairs that have a long enough merger timescale for a TDE to take place, i.e for $M_\bullet = 10^7 - 10^8\,M_\odot$, the perturber mass is in the range $m_{\rm per} = 26\,500 - 5700\,M_\odot$,

- $V_{\rm com}(< 0.06) \sim 0.068\,{\rm Gpc}^3$, which is a comoving volume within $z = 0.06$ for flat ΛCDM with $\Omega_{\rm m} = 0.3$.

Inserting these estimates into Eq. (S20) results in $N_{\rm pair} \sim 53200 - 700$ SMBH-IMBH pairs considering $M_\bullet = 10^7 - 10^8\,M_\odot$, with $N_{\rm pair} \sim 9200$ for $M_\bullet \sim 10^{7.4}\,M_\odot$ (for all the galaxies, considering both AGN and quiescent nuclei). Selecting those where a TDE takes place (considering the rate of $\dot{N}_{\rm TDE} \sim 10^{-4}\,{\rm yr}^{-1}$), we obtain $N_{\rm pair,TDE} = 0.07 - 5.3$ sources per year ($M_\bullet = 10^7 - 10^8\,M_\odot$) and $N_{\rm pair,TDE} = 0.9$ sources per year for $M_\bullet \sim 10^{7.4}\,M_\odot$. Hence, out of the total number of galaxies in a given cosmological volume (~ 2.5 million galaxies), we expect the TDE occurrence in a galaxy hosting the SMBH-IMBH pair in 1 out of $\sim 5 \times 10^5 - 36 \times 10^6$ galaxies.

Using the TDE rate of $\dot{N}_{\rm TDE} \sim 10^{-4}\,{\rm yr}^{-1}$ per galaxy, we can estimate the timescale on which it is expected we detect a TDE flare in galaxies hosting a tight SMBH-IMBH pair every $\tau_{\rm TDE-IMBH} \sim (\dot{N}_{\rm pair,TDE})^{-1} \sim 0.2 - 14.3$ years for the whole range $M_\bullet = 10^7 - 10^8\,M_\odot$, with $\tau_{\rm TDE-IMBH} \sim 1.1$ years for $M_\bullet = 10^{7.4}\,M_\odot$.

From an observational point of view, the number of sources with IMBH-induced QPOuts is $N_{\rm QPOut} = f_{\rm I} f_{\rm inc} N_{\rm pair}$, i.e. from the total number of galaxies with tight SMBH-IMBH pairs we are selecting those that we observe sufficiently close to the rotation axis (the fraction f_I, i.e. sources with the viewing angle less than 45° from the rotation axis so that the accretion flow is viewed close to face-on) as well as at the same time, the IMBH is highly inclined so that the ejected blob obscures the underlying flow (the fraction $f_{\rm inc}$, i.e. sources with the IMBH inclined at more than 45° from the equatorial plane). Assuming the uniform distribution of viewing-angle/inclination cosines, we get $f_{\rm I} \sim 0.71$ and $f_{\rm inc} \sim 0.29$, which yields $N_{\rm QPOut} \sim 0.21 N_{\rm pair}$

or 11 000–150 sources hosting SMBH-IMBH pairs can be revealed via QPOuts, i.e. one in 250 up to 5000 galaxies can exhibit QPOuts triggered by an IMBH (massive perturber).

Although there is a large uncertainty of nearly two orders of magnitudes in terms of the TDE occurrence in NSCs hosting a SMBH-IMBH pair, crude estimates provided here show that such an event is not entirely unlikely given the long-term monitoring of nearby AGN. We note that according to Eq. (S20) the number of expected SMBH-IMBH pairs does not depend e.g. on the considered merger timescale or the perturber mass since both $\tau_{\rm merge}$ and $T_{\rm df}$ are inversely proportional to $m_{\rm per}$, though in reality there likely is a dependency considering the fact that e.g. black holes of different masses are produced via different formation channels. On the other hand, the estimate of $N_{\rm pair}$ can be considered as a lower limit since we considered only AGN, while the TDE phenomenon occurring in NSCs hosting an IMBH is also relevant for quiescent nuclei, such as the Galactic center. Considering sources at an even larger redshift would also significantly extend the sample of the sources similar to ASASSN-20qc.

4.3 Inclination and Distance of the IMBH

A higher inclination of the perturber with respect to the SMBH's accretion disk is required to perturb the region close to the funnel/disk boundary where the material can be pushed into the outflow region and further accelerated by the ordered magnetic field in the funnel. When the IMBH migrates to the innermost regions of a few$\times$100 to $\sim$1000 gravitational radii from the NSC, the inclination distribution of the IMBH perturbers can be broad with a probability of ~ 0.7 for the perturber to be inclined between 45 and 135 degrees, assuming the isotropic distribution of orbits within the NSC, i.e. the uniform distribution of inclination cosines. Hence, inclined orbits with respect to the accretion disk are generally more likely than aligned orbits for an IMBH from within the NSC or beyond due to the isotropic massive cluster infall. In case there is a population of aligned compact remnants within the disk plane, e.g. due to the migration trap (*165*), then the inclination can be increased due to (a) recoil velocity kick due to black hole-black hole merger or (b) Kozai-Lidov eccentricity-inclination oscillations due to the presence of a massive body/disk at larger distances.

Case (a) is based on the fact that gravitational waves carry away linear momentum flux, hence during the binary black hole merger and the ring-down, the merger product receives a recoil velocity kick that can reach several hundred to thousand km/s (*166*). Assuming initially a circular orbit for a black-hole binary as well as a circular orbit for the inclined orbit of the formed IMBH, the required velocity kick to change the orbital inclination by $\Delta\iota$ is $\Delta v_{\rm kick} \sim 2 v_{\rm orb} \sin{(\Delta\iota/2)}$, where the orbits are also assumed to have a comparable semi-major axis and $\Delta v_{\rm kick}$ is perpendicular to the orbital velocity vector. For the inclination change of $\Delta\iota = 60^{\circ}$ from the disk plane to the inclined orbit crossing the disk, the required velocity kick is approximately equal to the orbital velocity, $\Delta v_{\rm kick} \approx v_{\rm orb}$. For $\Delta v_{\rm kick} \approx 1000\,{\rm km\,s^{-1}}$ and general $\Delta\iota$, this implies the effective distance from the SMBH where gravitational-wave recoil

kicks can lead to highly inclined orbits,

$$r_{\rm inc} = \left[2\frac{c}{\Delta v_{\rm kick}}\sin\left(\frac{\Delta\iota}{2}\right)\right]^2$$
$$= 5.3\times 10^4 \left(\frac{\Delta v_{\rm kick}}{1000\,{\rm km\,s^{-1}}}\right)^{-2}\sin^2\left(\frac{1}{2}\frac{\Delta\iota}{45^{\circ}}\right) r_{\rm g}\,, \quad \text{(S23)}$$

which is at least two orders of magnitude further than the inferred distance of the IMBH perturber. The merger timescale given by Eq. (S14) for the distance $r_{\rm inc}$ in Eq. (S23) is much longer than other relevent dynamical timescales, $\tau_{\rm merge} \sim 2.35\times 10^{16}\,{\rm yr}$. It is therefore more likely that the inclination increased due to repetitive mergers. For illustration, mergers taking place at $440\,r_{\rm g}$ with the average velocity kicks of $\sim 500\,{\rm km\,s^{-1}}$ can change the inclination step-wise by 2 degrees. Hence, between 10 to 100 consecutive mergers are needed for the initial stellar black hole to increase its mass to the intermediate mass range, while at the same time the inclination changes can add up to reach high values above the disk plane, depending on the mass ratios and spin distribution of merging black holes. A single high kick velocity of $\Delta v_{\rm kick} \sim 5000\,{\rm km\,s^{-1}}$ indicated by the recent gravitational-wave event analysis (*167*), which is perpendicular to the orbital plane, can increase the inclination by 17° with respect to the disk plane at the orbital distance of $\sim 300\,r_{\rm g}$ where the migration trap with several accumulated stellar black holes can be located (*165*).

Case (b) process – Kozai-Lidov mechanism – is based on the preservation of the z-component of the specific angular momentum in the inner three-body problem. Specifically, $(1-e_{\rm per}^2)^{1/2}\cos\iota_{\rm per}$ is constant, which implies the periodic oscillations from highly-inclined circular orbits to disk-embedded eccentric orbits. However, for the case of ASASSN-20qc and the likely perturbation by the distant dusty torus with the mass of $m_{\rm t} = 0.1M_{\bullet} \sim 10^{6.4}\,M_{\odot}$ and the torus distance of $r_{\rm t} \sim 10\,{\rm pc}$ (*168*), the oscillation timescale is longer than the Hubble time at the current distance of the IMBH (*169*),

$$T_{\rm KL} \sim 4\pi^2\frac{(GM_{\bullet})^{1/2}}{Gm_{\rm t}}\frac{r_{\rm t}^3}{r_{\rm per}^{3/2}}\,,$$
$$= 8\pi^3\frac{r_{\rm t}^3}{Gm_{\rm t}P_{\rm orb}}$$
$$\simeq 10^{15}\left(\frac{r_{\rm t}}{10\,{\rm pc}}\right)^3\left(\frac{m_{\rm t}}{10^{6.4}\,M_{\odot}}\right)^{-1}\left(\frac{P_{\rm orb}}{8.05\,{\rm d}}\right)^{-1}\,{\rm yr}\,, \quad \text{(S24)}$$

which implies that an inclined IMBH will likely stayed inclined during the inspiral unless the IMBH experiences an orbital decay due to the disk drag. The Kozai-Lidov timescale can be shortened in case the IMBH would be perturbed by another massive body closer than the torus, which is, however, speculative.

The grinding mechanism was explored extensively in the context of orbiting bodies around the SMBH. (*170*) were the first to recognize potential importance of hydrodynamical interaction

between stars and accretion disk in the nuclei of active galaxies and quasars fed via accretion. Starting from order-of-magnitude arguments and semi-analytical estimates these authors suggested that a long-term effect of the interaction should lead to secular changes of the stellar trajectories around a supermassive black hole: circularization (decrease of the osculating eccentricity) of the orbits and their monotonic sinking towards the center (decay of the semimajor axis) accompanied by the gradual "grinding" (decrease of inclination). The process eventually brings stars into the accretion disk plane. Relevant time-scales depend on several factors, most importantly, the ratio between the surface density of the accretion disk to the projected surface density of the star. For example, in the case of standard-type, geometrically thin, planar accretion disk (*171*), the orbital decay takes place over a vast range of 10^4–10^7 Keplerian periods of the stellar orbiter at the corresponding distance from the SMBH (*172*). After that time the star should become fully embedded into the accretion disk. Let us note that the effective cross-sectional area for the hydrodynamical interaction of a $10^5 M_\odot$ IMBH is comparable with that of a solar-type star usually considered in the quoted papers.

Evaluation of the grinding time requires to specify the accretion disk density profile as a function of radius; see eq. (24) in (*173*). For example, for a solar-type orbiter at radius $10^4 R_{\rm g}$ and the standard accretion disk with viscosity parameter $\alpha \approx 10^{-3}$, accretion rate $\dot{M}_\bullet = 1 M_\odot$ per year one obtains the grinding time about 10^6 revolutions, corresponding to $\simeq 10^8$ years. Precise temporal dependencies can be determined by direct numerical evaluation.

The above-mentioned simplistic scenario is based on a number of assumptions, which were further studied by various authors. First of all, the collisions should not expel excessive amount of gaseous material out of the accretion disk (*174, 175*), thereby threatening the accretion system. On the other hand, if the accretion flow becomes self-gravitating and its total mass is not negligible with respect to the central black hole, the orbiters are affected by Kozai-Lidov mechanism that causes continued oscillations of the osculating elements (eccentricity and inclination) (*169, 176, 177*), see Eq. (S24) for the Kozai–Lidov timescale. Hence, the perturber does not align with the disk plane. Similarly, a secondary massive black hole could induce a non-spherical perturbation of the central gravitational field, so that the orbital decay of the stellar satellites proceeds via continued exchange between eccentricity and inclination.

Several authors explored the process of orbital decay due to emission of gravitational waves by a gradually sinking stellar-mass orbiter; see, e.g., (*156, 178, 179*), and further references cited therein. They find that the direct hydrodynamical interaction with an accretion disk plays a dominant role in the orbital evolution of such satellites. However, geometrically thick ADAF structure is less efficient in grinding the inclination as the projected surface density is *orders of magnitude lower* than that of a standard accretion disk; the corresponding time-scales of orbital evolution are thus proportionally longer; at larger distances of $\gtrsim 10^2 R_{\rm g}$ it exceeds the Hubble time (even if it was argued (*180*) that turbulence within the medium may enhance the drag effects). IMBH has about the same effective cross-sectional area for the hydrodynamical interaction with gas as a solar-mass star but the mass of IMBH is five orders of magnitude higher than a normal star. Hence, the effect of collisions is weaker by that factor.

Here we remind the reader that in the case of ASASSN-20qc during the outburst, the outflow

rate at maxima reaches the value of $\dot{m}_{\rm out} \simeq 0.002 M_{\odot}/yr$ (see Sect. 11), while the accretion rate during the outburst was about thousand times higher than in the previous quiescent time. Assuming that the expelled amount of gas scales with the density of the accretion flow, the pre-outburst outflow rate can be estimated $\sim 10^{-6} M_{\odot}/yr$. Hence, the ratio of the mass expelled by the IMBH during one year to its own mass is $\Delta m_{\rm out}/m_{\rm per} \sim 10^{-10}$ and thus the dynamical effect on the IMBH orbit is negligible.

A quantitative comparison between the outer standard accretion disk and the inner ADAF can be done based on the analytical estimate of the grinding timescale as derived by (*170*),

$$t_{\rm grind} \sim \chi \frac{m_{\rm per}}{\Delta m} \frac{1}{(\Omega_{\rm K} P_{\rm orb})^{2/3}} P_{\rm orb}\,, \tag{S25}$$

where $\Delta m \sim \rho_{\rm flow} h_{\rm flow} \mathcal{R}^2$ is the mass pushed from the accretion flow with the mass density of $\rho_{\rm flow}$ and the scale-height of $h_{\rm flow}$ by the perturber with the influence radius $\mathcal{R}$. In Eq. (S25), the Keplerian angular velocity at the perturber distance $r_{\rm per}$ is denoted as $\Omega_{\rm K}$ and the corresponding orbital period by $P_{\rm orb}$. The numerical factor χ is set to 5 according to (*170*). Considering the thick ADAF with the scale-height to radius ratio close to unity, we can set $h_{\rm flow} \sim r_{\rm per}$. Furthermore, we set the viscosity parameter to $\alpha = 0.1$, the accretion rate normalized with respect to the Eddington rate to $\dot{m} = 0.05$, and the perturber influence radius to $\mathcal{R} = 3\,r_{\rm g}$ (its mass is set to $m_{\rm per} = 10^4\,M_{\odot}$, while the SMBH mass is kept at $M_{\bullet} = 10^{7.4}\,M_{\odot}$). At the distance of $r_{\rm per} \sim 93\,r_{\rm g}$, the grinding timescale for the ADAF flow is $t_{\rm grind} \sim 4.6 \times 10^9$ years, while for the standard thin disk, the grinding timescale is $t_{\rm grind} \sim 10^4$ years. Hence, for the ADAF, the timescale for the perturber alignment is 4.5×10^5 times longer than for the standard thin disk, independent of the perturber distance from the SMBH. The radial profiles of the grinding timescales for both the ADAF and the standard disk are shown in Fig. S15. It is clear that the grinding timescale for the IMBH-ADAF interaction is always larger than the merger timescale for the distance range of interest. This suggests that an initially inclined orbit of the IMBH with respect to the ADAF will not become aligned with the equatorial plane of the flow during the inspiral. The situation is more complex for the mixed flow with the thermal component since then the grinding timescale is shorter than the merger timescale outside $100\,r_{\rm g}$ (however, it is longer inside $\sim 100\,r_{\rm g}$, see Fig. S15). However, the detailed inclination evolution depends on the initial conditions and the ADAF extent. It would also be necessary to perform numerical calculations following orbital evolution during multiple passages through the standard disk and then the inner ADAF, which is beyond the scope of the current study.

There could be physical reasons why the SMBH-IMBH pair is detected at the separation of $100\,r_{\rm g}$ that we briefly discuss here. One is related to the variable density of the accretion flow along the radial direction, specifically the accretion disk transitions from the standard disk to ADAF at the radius given by Eq. (S9) or (S10). In addition, this radius is variable due to changing accretion rate with time. The density of the flow directly affects the hydrodynamic

drag timescale, i.e. the e-folding timescale of the IMBH specific angular momentum (*156*),

$$t_{\rm hd} = \frac{v_{\rm per} v_{\rm rel}^2}{4\pi I G^2 m_{\rm per} \rho_{\rm flow}}\,, \tag{S26}$$

where $v_{\rm per}$ is the Keplerian velocity of the perturber, $v_{\rm rel} \approx (2GM_\bullet/r)^{1/2}$ is the relative velocity with respect to the gas, $\rho_{\rm flow}$ is the mass density of the accretion flow, which can either correspond to a standard thin disk at larger distances or an ADAF closer in, and the factor I was already defined below Eq. (S13). To obtain order-of-magnitude quantitative comparison, we adopt $\dot{m} = 0.05$ for the accretion rate, the radiative efficiency is set to 0.1 and the viscosity parameter to 0.1 for both types of accretion flows. For the IMBH mass of $m_{\rm per} = 10^4\,M_\odot$, the ratio of timescales is $\sim 2 \times 10^8$ at $93\,r_{\rm g}$, and it does not evolve much with distance. Hence, when the disk transitions to ADAF, the hydrodynamic drag is eight orders of magnitude weaker and the e-folding timescale is prolonged from ~ 300 years to $\sim 5 \times 10^{10}$ years, which results in the IMBH stalling within the ADAF flow. The further orbital decay is then dominated by the gravitational emission. We depict the radial dependency of hydrodynamic timescales in Fig. S15, where we also show the grinding timescales corresponding to the inclined IMBH, its merger timescale, the Kozai-Lidov timescale due to the AGN torus perturbation, and general relativistic Schwarzschild and Lense-Thirring timescales.

Another mechanism that can lead to the IMBH orbiting at $\sim 100\,r_{\rm g}$ is the potential formation of migration traps in AGN disks, which are regions of equilibrium orbits with zero torque from the differentially rotating disk, or in other words, these are the distance ranges where the inward migration of embedded objects meets the outward migration. Bellovary et al. (*165*) used two steady-state, analytical disk solutions and derived the location of the migration trap between 40–600 $r_{\rm g}$, i.e. the region where the putative IMBH perturber in the ASASSN-20qc source is located. Within the migration trap, stellar black holes tend to accummulate, merge and scatter, which naturally leads to the efficient IMBH formation within 10 Myr. Mergers and dynamical scattering could eventually naturally create inclined orbits with respect to the underlying disk.

4.4 Caveats of the perturber–accretion disk interaction model

The basic caveat of 2D simulations in this study is the progressive weakening of the magneto-rotationally instability due to the lack of the toroidal flow. This is noticeable for the times later than $\sim 30\,000 GM_\bullet/c^3$. 3D simulations do not exhibit such a problem, however, they are computationally much more demanding for studying perturbations at $\sim\ 100\,r_{\rm g}$ due to much longer orbital period; this was the reason for performing the 3D runs with the perturber orbiting at $10\,r_{\rm g}$, for which a few cycles could have been studied. We tested the effect of the disappearing MRI in 2D runs by placing the perturber earlier to the state resulting from the unperturbed 2D flow – at $20\,000 r_{\rm g}/c$ (Run 1) and later at $50\,000\,r_{\rm g}/c$. In both cases, periodic outbursts in the outflow rate, i.e. QPOuts, are always present regardless of the MRI that is progressively weakening. This indicates that the QPOuts are induced by the perturber and the background

accretion flow is less relevant, i.e. even at late stages when MRI is suppressed, the perturber still causes QPOuts that appear comparable as to when the MRI is still active. However, here we note that after $50\,000\,r_{\rm g}/c$ or 71.5 days the mass-accretion rate drops consistently by two orders of magnitude with respect to the initial value, which is indicative of the magnetorotational instability weakening and hence the comparison of the simulation data with realistic accretion flows is limited at this stage. In contrast to the weak dependence of the fast outflow formation on the background accretion flow, the ordered and stable poloidal magnetic field in the funnel region appears necessary for accelerating the ejected blobs to relativistic velocities, therefore ordered poloidal magnetic field is a crucial element of the model.

2D and 3D GRMHD simulations presented here and performed using the HARM code neglect radiative feedback and radiative cooling. In case the inner part of the accretion flow transitions into the hot radiatively inefficient accretion flow (of ADAF type) at the radius comparable to or larger than the IMBH distance, this does not pose a problem. However, in case the inner disk does cool radiatively and still contains a thermal component, the accretion-disk evolution, perturbed by the IMBH, would deviate from the one presented here. Indeed, in case of a geometrically thin accretion disk, the dynamics of outflows is (almost) symmetrical with respect to the upper and lower hemispheres. This situation was originally modelled within the hydrodynamic framework (*181*). Considering both intersections of the trajectory with the disk plane would increase the effective rate of star-disk collisions and thus it would influence the model parameters, although the overall qualitative picture of the scenario remains unchanged. Moreover, it appears that strong jets powered by the MAD state and the associated semiregular magnetic eruptions cannot be sustained by a geometrically thin disk (*182, 183*). On the other hand, the backflow is expected to be relatively weaker for geometrically thick, magnetized tori, which we expect to persist from the prior low-luminous phase. Even if some material is expelled from the disk in the backward direction with respect to the orbiter's velocity vector, the forward push dominates. Furthermore, the flares due to shocks as the star impacts the thin standard disk should be revealed in the continuum emission (X-ray, UV, optical), which is not seen in this source (see also estimates below). Instead, we see quasiperiodic enhancements in the absorbing material that is ultrarelativistic. The perturber-thin disk interaction assumes a standard, optically thick disk, where the shock is more prominent due to a denser, cooler gas. In our model, we argue that the interaction takes place in the warmer and much more diluted ADAF component that is present due to a previous low-luminosity state of ASASSN-20qc, or more precisely, the standard outer disk transitions into ADAF outside the orbit of the perturber because of the lower Eddington ratio of ~ 0.05.

In addition, if we adopt the thin-disk setup, a star on a circular orbit would cross the disk twice, hence $P_{\rm orb} \sim$ 16.6 days. This implies the semi-major axis of $a_\star/r_{\rm g} \sim 150.62\,r_{\rm g}$ and the Keplerian velocity of $v_{\rm K} \sim c/\sqrt{a_\star/r_{\rm g}} \sim 0.08c$. The shocked gas should expand above and below the disk plane at the velocity of $v_{\rm sh} \sim \sqrt{2}v_{\rm K} \sim 0.1\,{\rm c}$, which is below the detected QPOut velocity of $0.3c$. Hence, the model would need some fine-tuning in terms of a significantly higher eccentricity, implying that QPOuts take place only at the pericenter. In that case, the orbital period is $P_{\rm orb} \sim 8.3$ days as originally assumed and the semi-major axis is $(a_\star/r_{\rm g}) \sim$

$95\,r_{\rm g}$. The eccentricity of the orbit would have to be $e \sim 0.7$ to reach shock speeds of $v_{\rm sh} \sim \sqrt{2}v_{\rm K} \sim 0.35c$. However, in that case, the pericenter of the orbit would be $28.5\,r_{\rm g}$, which would result in more profound quasiperiodic variability of the inflowing matter, which should be revealed in the X-ray domain. However, no significant quasiperiodicity of the X-ray continuum emission was found. In general, we do not expect significant periodic X-ray flux enhancements due to either IMBH or star interactions with the accretion flow, given the orbital radius of $\sim 100\,r_{\rm g}$ of the perturber corresponding to the QPOut periodicity of 8.05 days. When we use the model set-up involving the standard disk as in (*145*), the X-ray flares due to the launched shocked ejecta are below the quiescent X-ray level of ASASSN-20qc (see also the estimates above in Section 2). X-ray flares due to shocks are expected to be even fainter for the case of a more diluted ADAF. Moreover, the constraint for the perturber influence radius is expected to differ for the standard-disk scenario, though the relations for the influence radius–perturber mass correspondence, see Eqs. (S12) and (S13), would still hold since they do not depend explicitly on the accretion disk density. Also, stars and pulsars interacting with the denser disk would produce even smaller stagnation radii, hence their potential to reproduce the observed ultrafast outflow would be even smaller.

Observed X-ray spectra indicate the ASASSN-20qc accretion disk likely consists of both a colder, thermal component further away and a warmer, diffused ADAF-like comptonizing medium in the inner region. This is implied by the best-fit black-body temperature of the thermal continuum, $kT \sim 0.086\,{\rm keV}$, which corresponds to $T \sim 998\,000\,{\rm K}$. This temperature is higher than the characteristic temperature corresponding to the standard thin disk around the SMBH of $10^7 - 10^8\,M_{\odot}$, $T(10^7\,M_{\odot}) \sim 274\,000\,{\rm K}$ ($0.024\,{\rm keV}$) and $T(10^8\,M_{\odot}) \sim 87\,000\,{\rm K}$ ($0.007\,{\rm keV}$), which is accreting with the Eddington ratio of $0.1 - 0.01$, respectively, with 10% radiative efficiency and the inner radius at $6\,r_{\rm g}$. For the range of the SMBH masses and Eddington ratios of $\lambda_{\rm Edd} \lesssim 0.1$, the standard thin disk is expected to make a transition to the hot ADAF, e.g. due to thermal conduction, at the radius of $R_{\rm evap} \sim 132 - 448\,r_{\rm g}$, see Eq. (S10). The ADAF is characterized by the nearly virialized temperature profile for ions, $T_{\rm i} \sim 3.6 \times 10^{12}(r/r_{\rm g})^{-1}\,{\rm K}$ ($313\,{\rm MeV}(r/r_{\rm g})^{-1}$), while the electrons cool down via bremsstrahlung, synchrotron, and Compton processes to $T_{\rm e} \sim 10^9\,{\rm K}$ ($86\,{\rm keV}$). This hot diluted medium can then serve as the comptonizing environment for the underlying thermal disk emitting softer photons. Given the Eddington rate of $\sim 0.05 - 0.5$ during the X-ray outburst, and the low Eddington rate of 2×10^{-5} before that, ASASSN-20qc likely transitioned from the hard to the soft state, and back again, with the ADAF region shrinking and extending again with the change in the accretion rate according to Eq. (S9). Its outburst Eddington ratio also appears to indicate that the source could be found in the intermediate state, i.e. when the ADAF recondenses back to the thin disk at the recondensation radius of $r_{\rm con} = 25(\alpha/0.2)^{-28/3}(\dot{m}/0.01)^{8/3}r_{\rm g}$ as the accretion rate decreases (*184*). The inflection point of the 2-10 keV photon index-Eddington ratio correlation lies close to $\lambda_{\rm Edd} \sim 0.05$ (*185*), which indicates the mixed, non-typical accretion state of ASASSN-20qc sharing the properties of both low-luminosity and high-luminosity AGN. The peak of the Eddington-ratio distribution of changing-look AGN also lies close to $\lambda_{\rm Edd} \sim 0.01$ (*186*). The thin disk solution including general relativistic effects, in particular

the Kerr metric, can indeed address the higher temperature of 0.086 keV for a highly accreting SMBH (see Methods section 6). Such a thin disk emitting thermal X-rays is rather compact, extends within the tidal radius of the tidally disrupted star, and is embedded in the pre-existing optically thin ADAF.

In summary, to fully capture the dynamics of the perturbed flow, we would need to address the increased accretion rate following the TDE, which presumably leads to the inner thin disk formation on the length-scale of the tidal radius that cools radiatively. The radiatively inefficient ADAF component, which is a remnant of the previous low-luminosity state, continues on the length-scales beyond the tidal radius. It is likely that the accretion flow transitions to the standard thin disk further out as implied by the detected broad lines indicative of the standard disk (*147*) because of the established radius-luminosity relations.

The assumption of fully synchronized motion of the gas with the perturber within the influence radius does not capture the detailed structure of the flow in the vicinity of the secondary body. In particular, we do not follow the radial outflow of the stellar wind in case of a star, nor the motion of the gas directly governed gravitationally by the secondary inside the bow shock and wake in case of a black hole. The velocity distribution may be quite complex and may change on a small length scale compared with the influence radius, which is,however, beyond the resolution of our simulations. However, since the bow shock is comoving with the perturber, its main dynamical effect on the surrounding medium is captured reasonably well by our simulations.

In spite of several simplifications in our GRMHD simulations, the lead-order general relativistic magnetohydrodynamical effects of the pertuber-disk interaction are captured with a sufficient precision and imply the recurrent ultrafast outflow generation at the perturber-disk interaction site as long as the magnetic field is sufficiently ordered in the inner region. The ejected blobs are outflowing along the funnel-accretion disk boundary, where they cross the line of sight and cause periodic absorption of the underlying thermal disk.

In addition, since in the inclined perturber model, the IMBH is expected to be misaligned with respect to the equatorial plane, its orbit would precess due to the frame-dragging, as we discussed in Subsection 4.1. Though the $90°$ Lense-Thirring timescale, during which the outflow is expected to change the direction away from the observer, is at least an order of magnitude longer than the observed QPOut cycle (2255 days for the SMBH spin of 0.4, see Eq. (S18)), it could in principle lead to the cessation of QPOuts within a few year timescale, especially for a fast rotating black hole (in 1002 days or 2.7 years, the outflow direction would change by $90°$ for the spin of $a = 0.9$). In case QPOuts would cease to be detected within several years, it would strengthen the case for the perturber-induced scenario, and it would provide a unique way to constrain the SMBH spin (given that the SMBH mass is well constrained). On the other hand, the ejected absorbing gas clump will also expand as it is accelerated downstream, making the perturber-induced outflow less sensitive to the orbital orientation. In addition, the blob trajectory is not necessarily linear as it is accelerated by the magnetic field, it can follow e.g. the helical magnetic-field configuration, which would lead to the absorbing events for any orientation of the perturber orbit. However, high-cadence monitoring of ASASSN-20qc is challenging

because of the decrease in the X-ray flux density following the TDE.

Date dd/mm/yyyy	MJD	Δt Days	F_ν mJy	ΔF_ν mJy
04/05/2019	58607	−596	0.96	0.34
27/08/2019	58723	−480	0.77	0.25
29/10/2019	58786	−417	0.94	0.24
30/10/2019	58786	−416	1.40	0.25
10/01/2020	58859	−344	0.80	0.47
24/01/2020	58873	−331	1.25	0.22
25/01/2020	58874	−330	1.21	0.21
20/06/2020	59020	−183	1.45	0.25
28/08/2020	59090	−113	1.24	0.24
24/07/2021	59419	216	1.35	0.24
22/08/2021	59449	246	1.13	0.22

Table S1: ASASSN-20qc's radio (VAST) observations. Δt is the time since December 20, 2020. F_ν is the peak flux density in mJy, and ΔF_ν is its uncertainty in mJy.

Quantities	2021/01/11	2021/07/30	2021/08/19	2021/11/09	2022/01/25	2022/03/10	2022/08/26
L5100	43.97 ± 0.01	43.84 ± 0.01	43.79 ± 0.01	43.66 ± 0.01	43.60 ± 0.01	43.60 ± 0.01	43.56±0.01
FWHM(Hβ_{bc})	2108 ± 183	2722 ± 204	2087 ± 396	2664 ± 75	2718 ± 56	2444 ± 53	3199 ± 96
L(Hβ_{bc})	41.48 ± 0.03	42.14 ± 0.03	42.31 ± 0.02	42.14 ± 0.01	42.19 ± 0.01	42.21 ± 0.01	41.62 ± 0.06
L(Hβ_{nc})	40.60 ± 0.19	39.05 ± 1.63	40.54 ± 1.04	40.83 ± 0.04	41.04 ± 0.03	40.93 ± 0.04	40.83 ± 0.03
L(O[III]5007)	40.97 ± 0.07	41.18 ± 0.08	41.40 ± 0.04	41.17 ± 0.01	41.26 ± 0.01	41.32 ± 0.01	41.36 ± 0.08
L(HeI5876$_{bc}$)	–	41.76 ± 0.03	41.75 ± 0.01	41.43 ± 0.01	41.48 ± 0.01	41.48 ± 0.01	41.52 ± 0.03
FWHM(HeI5876$_{bc}$)	–	17724 ± 1914	9386 ± 113	5020 ± 56	6242 ± 134	6323 ± 278	22489 ± 2304
FWHM(Hα_{bc})	2654 ± 441	3090 ± 151	2680 ± 211	3009 ± 77	2751 ± 553	2748 ± 103	2870 ± 8
L(Hα_{bc})	41.81 ± 0.04	42.59 ± 0.04	42.95 ± 0.04	42.69 ± 0.01	42.84 ± 0.05	42.87 ± 0.04	42.65 ± 0.01
L(Hα_{NA})	41.41 ± 0.02	41.55 ± 0.05	41.93 ± 0.06	41.61 ± 0.01	41.61 ± 0.27	41.67 ± 0.07	41.3 ± 0.01
L([NII]6585)	41.28 ± 0.03	41.45 ± 0.27	41.13 ± 1.47	41.21 ± 0.32	–	–	41.09 ± 0.01
$\log M_\bullet$	7.50 ± 0.08	7.66 ± 0.07	7.40 ± 0.16	7.54 ± 0.02	7.53 ± 0.02	7.43 ± 0.02	7.64 ± 0.03

Table S2: Spectral properties at different epochs after flux re-scaling based on photometry. The luminosity are given in logarithm scale and in erg/s unit. The FWHM and $M_\bullet$ are in the units of km s^{-1} and M$_\odot$, respectively.

Band	Observed (AB mag)	Model (AB mag)
GALEX FUV	19.84 ± 0.13	19.96 ± 0.14
GALEX NUV	19.27 ± 0.06	19.30 ± 0.06
DES g	16.36 ± 0.01	16.36 ± 0.01
DES r	15.81 ± 0.01	15.83 ± 0.01
DES i	15.60 ± 0.01	15.59 ± 0.01
DES z	15.41 ± 0.01	15.43 ± 0.01
DES Y	15.30 ± 0.05	15.36 ± 0.01
2MASS J	15.17 ± 0.09	15.27 ± 0.01
2MASS H	15.08 ± 0.12	15.17 ± 0.01
2MASS Ks	15.23 ± 0.13	15.40 ± 0.01
WISE W1	15.85 ± 0.25	15.82 ± 0.01
WISE W2	16.05 ± 0.25	16.08 ± 0.03
UVOT W2	—	19.45 ± 0.07
UVOT M2	—	19.32 ± 0.06
UVOT W1	—	18.94 ± 0.04
UVOT U	—	17.89 ± 0.01
UVOT B	—	16.69 ± 0.01
UVOT V	—	16.09 ± 0.01

Table S3: Results from host galaxy SED model fitting.

Table S4: **Summary of *XMM-Newton* X-ray energy spectral modeling of ASASSN-20qc**. Spectra are fit with the same model used for time-resolved *NICER* spectral analysis, i.e., *tbabs*ztbabs*xstar*zashift(diskbb)*. **ID** is the identifier used to refer to this observation. **Observation ID** is the ID assigned by *XMM-Newton*. **Start** and **End** represent the start and end times (in units of MJD) of the exposures used. **Exposure** is the total exposure time after removing intervals of background flaring. **nH**$_{\rm neutral}$ is the neutral column density (in units of 10^{21} cm^{-2}). Cases where **nH**$_{\rm neutral}$ is pegged to zero by XSPEC are indicated by "0.0". **nH**$_{\rm abs}$ is the column density of the ionized gas (in units of 10^{21} cm^{-2}). **Log**ξ is the Logarithm of the ionization parameter. $v_{\rm out}$ is the outflow speed with respect to us in units of c, the speed of light. **kT** is the temperature of the ionizing blackbody disk (in units of keV). **Norm** is the normalization of the *diskbb* model in units of 10^4. **Log(Integ. Lum.)** is the logarithm of the integrated blackbody luminosity in 1 eV-10 keV in units of erg s^{-1}. **Log(Obs. Lum.)** and **Log(unabs. Lum.)** are the logarithms of the observed and unabsorbed 0.3-1.1 keV luminosities in units of erg s^{-1}, respectively. **best-fit statistic/dof** is the best-fit value of statistic (χ^2 or C-statistic) over the degrees of freedom. All errorbars represent 1-σ uncertainties. **statistic** used for fitting (χ^2 for high count rate spectra and C-stat when the count rate was low). *Fixed to the best-fit value.

Best-fit parameters from *XMM-Newton* energy spectral modeling.

**ID**	**Observation ID**	**Start** (MJD)	**End** (MJD)	**Exposure** (ks)	**nH**$_{\rm neutral}$ (10^{21} cm^{-2})	**nH**$_{\rm abs}$ (10^{21} cm^{-2})	**Log**ξ	$v_{\rm out}$	**kT** (keV)	**Norm**	**Log(Integ. Lum.)** (1eV-10 keV)	**Log(Obs. Lum.)** (0.3-1.1 keV)	**Log(unabs. Lum.)** (0.3-1.1 keV)	**best-fit statistic/dof** used	**Fit statistic**
XMM1	0852600301	59287.359	59288.039	16.6	0.0	$4.0^{+3.7}_{-1.0}$	$1.8^{+0.2}_{-0.2}$	$-0.332^{+0.013}_{-0.012}$	$0.096^{+0.002}_{-0.002}$	$1.86^{+0.18}_{-0.16}$	$44.34^{+0.011}_{-0.011}$	$43.53^{+0.01}_{-0.02}$	$43.81^{+0.026}_{-0.013}$	19.6/16	χ^2
XMM2	0891800101	59416.775	59417.057	6.6	0.0	-	-	-	$0.091^{+0.012}_{-0.012}$	$0.02^{+0.03}_{-0.01}$	$42.30^{+0.20}_{-0.20}$	$41.52^{+0.08}_{-0.05}$	$41.73^{+0.10}_{-0.10}$	33/31	C-statistic
XMM3	0891803701 and 0891803801	59552.545	59556.749	36	0.0	$8.3^{+1.0}_{-2.0}$	$1.0^{+0.1}_{-0.1}$	$-0.22^{+0.05}_{-0.05}$	$0.117^{+0.025}_{-0.011}$	<0.2	$43.18^{+0.29}_{-0.55}$	$41.59^{+0.34}_{-0.55}$	$42.79^{+0.27}_{-0.48}$	99.1/88	C-statistic
XMM4	0893810701	59615.36	59615.54	10	0.0	-	-	-	$0.1^{+0.03}_{-0.03}$	$0.02^{+0.02}_{-0.01}$	$42.0^{+0.20}_{-0.16}$	$41.48^{+0.06}_{-0.10}$	$41.64^{+0.08}_{-0.08}$	52/38	C-statistic

Table S5: **Summary of time-resolved X-ray energy spectral modeling of ASASSN-20qc** . Here *NICER* spectra corresponding to the mimina and the maxima in outflow deficit ratio (ODR) are fitted with *tbabs*ztbabs*xstar*zashift(diskbb)* model (See SI). **Start** and **End** represent the start and end times (in units of MJD) of the interval used to extract a combined *NICER* spectrum. **Exposure** is the accumulated exposure time during this phase/time interval. **FPMs:** The total number of active detectors minus the "hot" detectors. **Phase** is the name used to identify the epoch. $\mathbf{nH}_{\rm neutral}$ is the neutral column density (in units of 10^{21} cm^{-2}). Cases where $\mathbf{nH}_{\rm neutral}$ is pegged to zero by XSPEC are indicated by "0.0". $\mathbf{nH}_{\rm abs}$ is the column density of the ionized outflow (in units of 10^{21} cm^{-2}). **Log**ξ is the Logarithm of the outflow's ionization parameter. $v_{\rm out}$ is the outflow speed with respect to us in units of c, the speed of light. **kT** is the temperature of the ionizing blackbody disk (in units of keV). **Norm** is the normalization of the *diskbb* model in units of 10^4. **Log(Integ. Lum.)** is the logarithm of the integrated blackbody luminosity in 1 eV-10 keV in units of erg s^{-1}. **Log(Obs. Lum.)** and **Log(unabs. Lum.)** are the logarithms of the observed and unabsorbed 0.3-1.1 keV luminosities in units of erg s^{-1}, respectively. **Count Rate** is the background-subtracted *NICER* count rate in 0.3-1.1 keV in units of counts/sec/FPM. All errorbars represent 1-σ uncertainties. χ^2**/dof** represents the best-fit χ^2 and the degrees of freedom.

**Start** (MJD)	**End** (MJD)	**Exposure** (ks)	**FPMs**	**Phase**	$\mathbf{nH}_{\rm neutral}$ (10^{21} cm^{-2})	$\mathbf{nH}_{\rm abs}$ (10^{21} cm^{-2})	**Log**ξ	$v_{\rm out}$	**kT** (keV)	**Norm**	**Log(Integ. Lum.)** (1eV-10 keV)	**Log(Obs. Lum.)** (0.3-1.1 keV)	**Log(unabs. Lum.)** (0.3-1.1 keV)	**Count rate** (0.3-1.1 keV)	χ^2**/dof**
Best-fit parameters from fitting spectra from **minima** in outflow deficit ratio															
59266.93	59273.8	3.73	50	Min1	0.0	$16.0^{+4.1}_{-8.0}$	$2.0^{+0.2}_{-0.2}$	$-0.35^{+0.023}_{-0.019}$	$0.082^{+0.004}_{-0.004}$	$11.46^{+5.34}_{-6.5}$	$44.25^{+0.11}_{-0.14}$	$43.16^{+0.13}_{-0.16}$	$43.72^{+0.08}_{-0.19}$	0.038±0.001	3.5/9
59275.6	59285.32	6.95	49	Min2	0.0	$12.6^{+2.2}_{-2.2}$	$2.3^{+0.4}_{-0.2}$	$-0.366^{+0.011}_{-0.01}$	$0.093^{+0.002}_{-0.002}$	$1.76^{+0.22}_{-0.18}$	$44.19^{+0.05}_{-0.05}$	$43.38^{+0.03}_{-0.07}$	$43.73^{+0.1}_{-0.06}$	0.064±0.0007	9/10
59287.47	59290.79	3.02	48	Min3	0.0	$10.1^{+2.7}_{-2.9}$	$2.0^{+0.2}_{-0.2}$	$-0.37^{+0.012}_{-0.012}$	$0.106^{+0.003}_{-0.003}$	$1.27^{+0.15}_{-0.12}$	$44.29^{+0.09}_{-0.08}$	$43.51^{+0.08}_{-0.06}$	$43.91^{+0.11}_{-0.09}$	0.0892±0.0014	10.5/9
59292.3	59302.5	15.21	46	Min4	0.0	$9.9^{+1.6}_{-1.6}$	$2.2^{+0.2}_{-0.1}$	$-0.359^{+0.009}_{-0.009}$	$0.096^{+0.002}_{-0.002}$	$1.56^{+0.11}_{-0.1}$	$44.2^{+0.05}_{-0.04}$	$43.42^{+0.04}_{-0.04}$	$43.76^{+0.06}_{-0.05}$	0.0702±0.0004	6.5/11
59303.7	59317.38	5.85	50	Min5	0.0	$7.1^{+2.4}_{-3.1}$	$2.2^{+0.7}_{-0.2}$	$-0.351^{+0.02}_{-0.017}$	$0.09^{+0.002}_{-0.002}$	$1.88^{+0.16}_{-0.13}$	$44.15^{+0.06}_{-0.05}$	$43.37^{+0.08}_{-0.1}$	$43.66^{+0.07}_{-0.06}$	0.0629±0.0007	12.1/10
59318.94	59335.15	5.72	47	Min6	0.0	$12.6^{+2.6}_{-2.8}$	$2.1^{+0.2}_{-0.1}$	$-0.372^{+0.012}_{-0.011}$	$0.095^{+0.002}_{-0.002}$	$2.09^{+0.25}_{-0.12}$	$44.29^{+0.08}_{-0.08}$	$43.42^{+0.05}_{-0.06}$	$43.84^{+0.09}_{-0.09}$	0.0695±0.0009	14.6/10
59337.12	59343.15	7.01	48	Min7	0.0	$2.8^{+0.8}_{-0.8}$	$1.8^{+0.2}_{-0.3}$	$-0.344^{+0.016}_{-0.013}$	$0.103^{+0.002}_{-0.001}$	$1.16^{+0.08}_{-0.07}$	$44.2^{+0.03}_{-0.01}$	$43.55^{+0.02}_{-0.05}$	$43.79^{+0.01}_{-0.01}$	0.0965±0.0007	6.5/10
59345.74	59351.99	5.36	48	Min8	0.0	$21.4^{+5.5}_{-4.9}$	$2.9^{+0.1}_{-0.1}$	$-0.306^{+0.021}_{-0.017}$	$0.109^{+0.002}_{-0.002}$	$0.94^{+0.08}_{-0.06}$	$44.21^{+0.06}_{-0.02}$	$43.56^{+0.03}_{-0.08}$	$43.83^{+0.06}_{-0.05}$	0.0986±0.0009	7.5/10
59352.96	59359.88	2.48	48	Min9	0.0	$21.5^{+7.5}_{-2.9}$	$3.2^{+0.2}_{-0.1}$	$-0.228^{+0.019}_{-0.022}$	$0.101^{+0.002}_{-0.002}$	$1.08^{+0.18}_{-0.1}$	$44.12^{+0.07}_{-0.01}$	$43.5^{+0.01}_{-0.15}$	$44.03^{+0.47}_{-0.15}$	0.087±0.0014	9.8/9
59361.57	59366.53	15.0	48	Min10	$0.3^{+0.05}_{-0.05}$	$15.0^{+-15.0}_{-15.0}$	$2.4^{+0.0}_{-0.2}$	$-0.324^{+0.009}_{-0.009}$	$0.075^{+0.002}_{-0.002}$	$6.66^{+1.0}_{-1.5}$	$44.33^{+0.05}_{-0.05}$	$43.13^{+-0.0}_{-0.04}$	$43.71^{+0.06}_{-0.04}$	0.0359±0.0003	18.8/10
Best-fit parameters from fitting spectra from **maxima** in outflow deficit ratio															
59273.8	59275.6	1.37	50	Max1	-	-	-	-	$0.084^{+0.002}_{-0.002}$	$2.2^{+0.3}_{-0.3}$	$44.07^{+0.02}_{-0.02}$	$43.35^{+0.01}_{-0.01}$	$43.54^{+0.01}_{-0.01}$	0.0611±0.001	15.7/11.0
59285.32	59287.47	0.6	48	Max2	-	$2.5^{+0.0}_{-2.5}$	$1.8^{+0.5}_{-0.3}$	$-0.341^{+0.029}_{-0.026}$	$0.116^{+0.005}_{-0.005}$	$0.6^{+0.2}_{-0.1}$	$44.17^{+0.04}_{-0.02}$	$43.59^{+0.0}_{-0.09}$	$43.82^{+0.05}_{-0.04}$	0.1096±0.002	6.9/8.0
59290.79	59292.3	1.15	48	Max3	-	$1.0^{+1.1}_{-0.4}$	$0.8^{+0.2}_{-0.3}$	$-0.331^{+0.02}_{-0.021}$	$0.116^{+0.002}_{-0.003}$	$0.6^{+0.1}_{-0.0}$	$44.11^{+0.03}_{-0.01}$	$43.53^{+0.03}_{-0.15}$	$43.76^{+0.03}_{-0.02}$	0.0976±0.0014	11.7/8.0
59302.5	59303.7	2.78	48	Max4	-	$1.2^{+1.5}_{-0.3}$	$0.8^{+0.5}_{-0.1}$	$-0.332^{+0.015}_{-0.014}$	$0.115^{+0.002}_{-0.003}$	$0.6^{+0.1}_{-0.1}$	$44.09^{+0.06}_{-0.05}$	$43.47^{+0.04}_{-0.12}$	$43.79^{+0.06}_{-0.06}$	0.0848±0.0008	10.9/9.0
59317.38	59318.94	1.04	52	Max5	-	$2.3^{+3.3}_{-0.9}$	$2.0^{+0.5}_{-0.5}$	$-0.304^{+0.036}_{-0.032}$	$0.11^{+0.004}_{-0.004}$	$0.8^{+0.1}_{-0.1}$	$44.17^{+0.02}_{-0.02}$	$43.58^{+0.02}_{-0.03}$	$43.8^{+0.02}_{-0.03}$	0.1079±0.0014	6.6/9.0
59335.15	59337.12	1.85	48	Max6	-	$2.6^{+3.2}_{-0.7}$	$1.6^{+0.2}_{-0.5}$	$-0.344^{+0.014}_{-0.014}$	$0.11^{+0.003}_{-0.007}$	$0.8^{+0.7}_{-0.1}$	$44.19^{+0.14}_{-0.02}$	$43.56^{+-0.0}_{-0.11}$	$43.82^{+0.11}_{-0.02}$	0.1007±0.0011	7.8/9.0
59343.15	59345.74	2.83	47	Max7	-	$1.0^{+1.2}_{-0.5}$	$0.9^{+0.4}_{-0.2}$	$-0.347^{+0.03}_{-0.02}$	$0.109^{+0.001}_{-0.003}$	$1.0^{+0.2}_{-0.1}$	$44.23^{+0.05}_{-0.02}$	$43.63^{+0.04}_{-0.11}$	$43.86^{+0.05}_{-0.03}$	0.1206±0.001	6.4/7.0
59351.99	59352.96	0.6	49	Max8	-	-	-	-	$0.094^{+0.002}_{-0.002}$	$1.5^{+0.2}_{-0.2}$	$44.14^{+0.02}_{-0.02}$	$43.5^{+0.01}_{-0.01}$	$43.68^{+0.01}_{-0.01}$	0.0846±0.0018	9.4/9.0
59359.88	59361.57	0.24	48	Max9	-	-	-	-	$0.095^{+0.004}_{-0.004}$	$1.4^{+0.4}_{-0.3}$	$44.13^{+0.04}_{-0.04}$	$43.51^{+0.01}_{-0.02}$	$43.69^{+0.02}_{-0.02}$	0.0896±0.003	6.6/7.0

Phase	r	$\dot{M}_{out}$	$\dot{E}_{out}$	$\frac{\dot{E}_{out}}{L}$	$\frac{\dot{M}_{out}}{\dot{M}_{acc}}$
	(r_g)	$(10^{-3}M_{\odot}/yr)$	(10^{42} erg/s)		
NICER Min phases					
Min1	16.4	2.3	8.2	0.16	0.26
Min2	15.0	1.9	7.2	0.13	0.20
Min3	14.6	1.4	5.6	0.07	0.10
Min4	15.4	1.4	5.3	0.09	0.14
Min5	16.2	1.1	3.7	0.08	0.13
Min6	14.4	1.7	6.9	0.10	0.14
Min7	16.8	0.4	1.4	0.02	0.03
Min8	21.4	3.6	9.7	0.14	0.31
Min9	38.4	4.9	7.2	0.07	0.27
Min10	19.0	2.4	7.1	0.14	0.26
NICER Max phases					
Max1	-	-	-	-	-
Max2	17.2	0.4	1.3	0.02	0.03
Max3	18.2	0.1	0.5	0.01	0.01
Max4	18.2	0.2	0.6	0.01	0.02
Max5	21.6	0.4	1.0	0.02	0.04
Max6	16.8	0.4	1.3	0.02	0.03
Max7	16.6	0.1	0.5	0.01	0.01
Max8	-	-	-	-	-
Max9	-	-	-	-	-
XMM phases					
XMM1	18.1	0.6	2.0	0.03	0.05
XMM2	-	-	-	-	-
XMM3	26.6	3.3	7.1	0.82	2.17
XMM4	-	-	-	-	-

Table S6: Conservative estimates of the location and energetics of the outflow detected in the time-resolved NICER analysis and in the XMM-Newton spectra. Phase is the name used to identify the epoch. r is the launching radius in units of the gravitational radius ($r_g = GM_{\bullet}/c^2$). $\dot{M}_{out}$ is the mass outflow rate in units of $10^{-3}M_{\odot}/yr$. $\dot{E}_{out}$ is the outflow kinetic power in units of 10^{42} erg/s. $\dot{E}_{out}/L$ is the ratio between the outflow kinetic power and the unabsorbed luminosity in the 0.3-1.1 keV band. $\dot{M}_{out}/\dot{M}_{acc}$ is the ratio between the mass outflow rate and the mass accretion rate estimated as $\dot{M}_{acc} = L/\eta c^2$, with $\eta = 0.1$.

GRMHD simulations									
Run	a	B	$t_{\rm in}$ (r_g/c)	$t_{\rm f}$ (r_g/c)	$r_{\rm per}$ (r_g)	$\log{(M_\bullet)}$ [$M_\odot$]	$\mathcal{R}$ (r_g)	ι (°)	Resolution
Run 1	0.4	a)	20 000	100 000	93	7.4	2	72	384 x 256
Run 2	0.4	b)	50 000	200 000	93	7.4	2	72	384 x 256
Run 3	0.4	a)	50 000	200 000	93	7.4	2	72	384 x 256
Run 4	0.4	a)	50 000	200 000	60 – 125	7.4	2	66	384 x 256
Run 5	0.4	a)	50 000	200 000	40	7.95	1	72	384 x 256
Run 6	0.4	a)	50 000	200 000	93	7.4	3	72	384 x 256
Run 7	0.4	a)	50 000	200 000	93	7.4	4	72	384 x 256
Run 8	0.4	a)	50 000	200 000	93	7.4	8	72	384 x 256
Run 9	0	b)	50 000	53 000	10	–	1	81	384 x 256 x 128
Run 10	0	b)	50 000	200 000	10	–	1	81	384 x 256
Run 11	0	b)	50 000	200 000	10	–	0.1	81	384 x 256
Run 12	0.4	a)	20 000	100 000	45 – 136	7.4	2	67	512 x 320
Run 13	0.4	a)	20 000	100 000	45 – 136	7.4	1	67	640 x 512
Run 14	0.4	a)	20 000	100 000	45 – 136	7.4	3	67	512 x 320
Run 15	0.4	a)	20 000	100 000	30 – 151	7.4	2	66	512 x 320
**Run 16**	0.5	a)	10 000	14 300	10 – 14.7	–	2	68	384 x 256 x 96

Table S7: **Parameters of GRMHD runs.** We list the dimensionless spin of SMBH a, initial magnetic field configuration: **a) more loops, b) 1 loop** , transient time after which the perturber is added into the flow $t_{\rm in}$, **final time** $t_{\rm f}$ the distance of the perturber from SMBH $r_{\rm per}$, the logarithm of the SMBH mass considered to derive the distance, influence radius of the perturber $\mathcal{R}$, inclination ι of the perturber orbit with respect to the equatorial plane, and the resolution of the run in terms of the number of radial logarithmic bins times the number of bins in θ direction $(r \times \theta)$. For the 3D Runs 9 and 16, the resolution pertains to the number of bins in r, θ, and ϕ directions.

FIG+++FIG+++FIG+++FIG+++FIG+++FIG+++FIG+++FIG+++FIG+++FIG+++FIG+++FIG+++

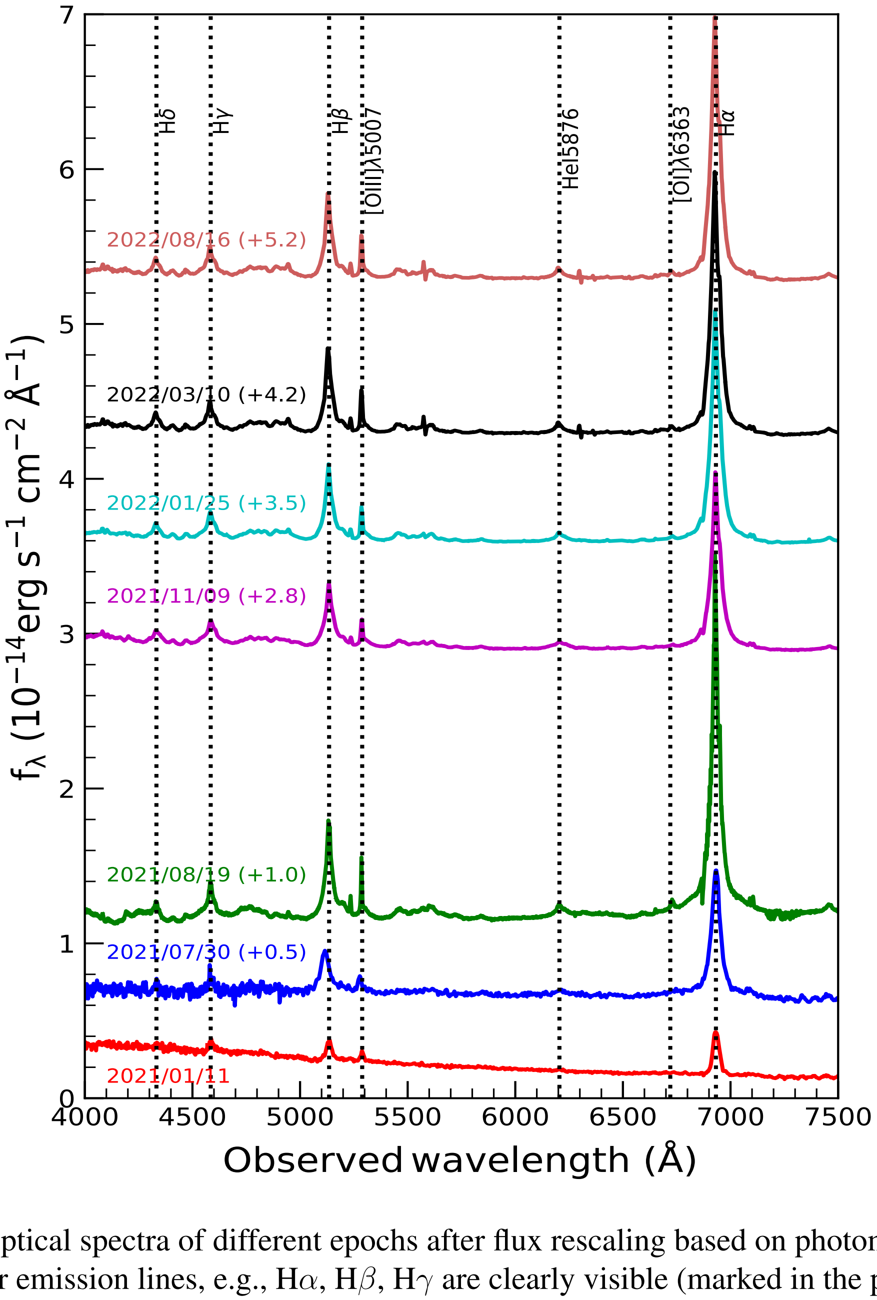

Figure S1: Optical spectra of different epochs after flux rescaling based on photometry. Strong broad Balmer emission lines, e.g., Hα, Hβ, Hγ are clearly visible (marked in the plot).

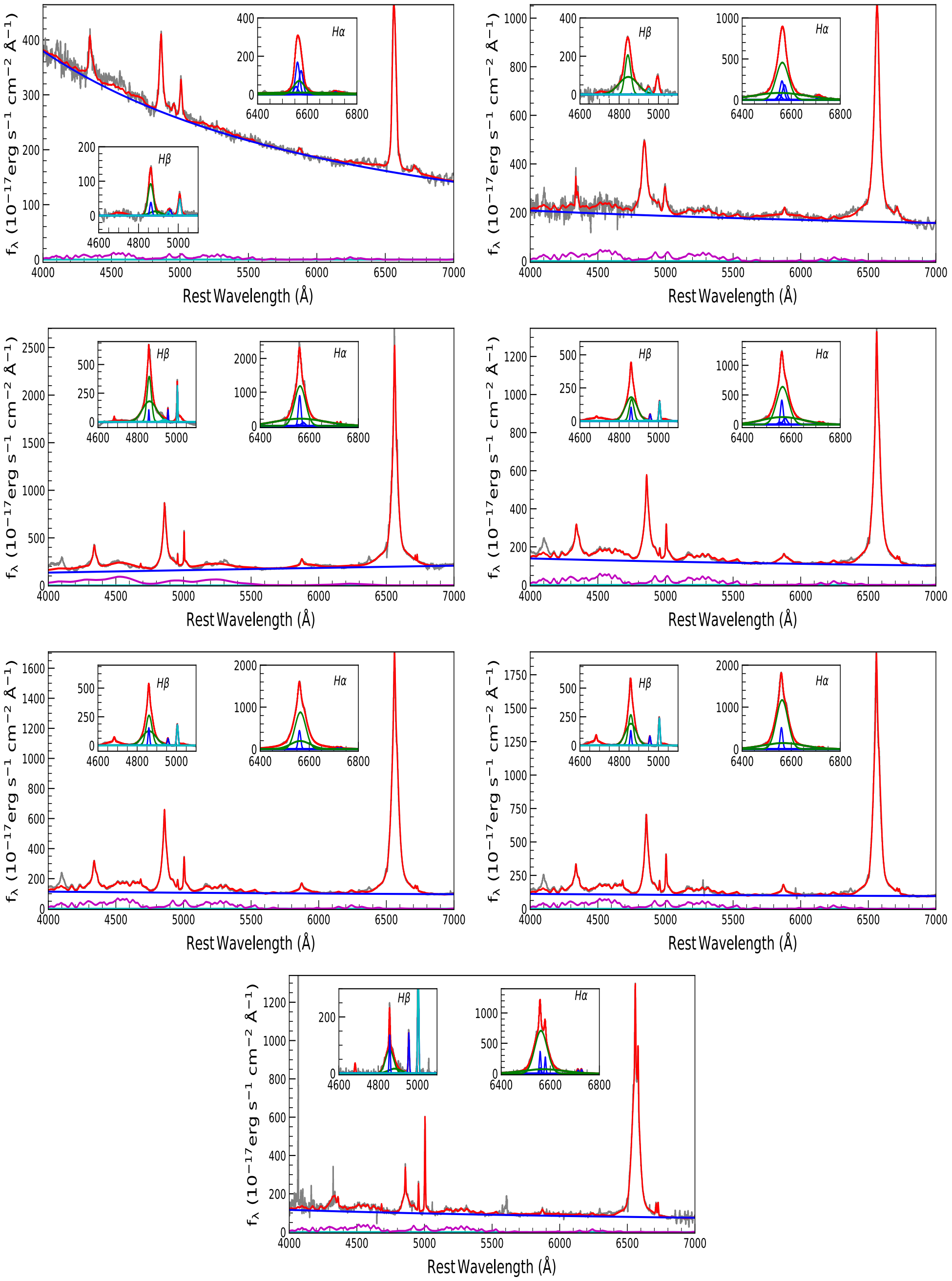

Figure S2: The optical spectral decomposition are shown for different epochs (from top to bottom 2021/01/11, 2021/07/30, 2021/08/19, 2021/11/09, 2022/01/25, 2022/03/10 and 2022/08/16). The data is shown in black while the best-fit entire model is shown in red. The decomposed continuum power-law and Fe II emission are shown by blue and magenta colors, respectively. The inset plots shows the zoomed-in version of Hβ and Hα complex where broad components are shown in green and narrow components are in blue.

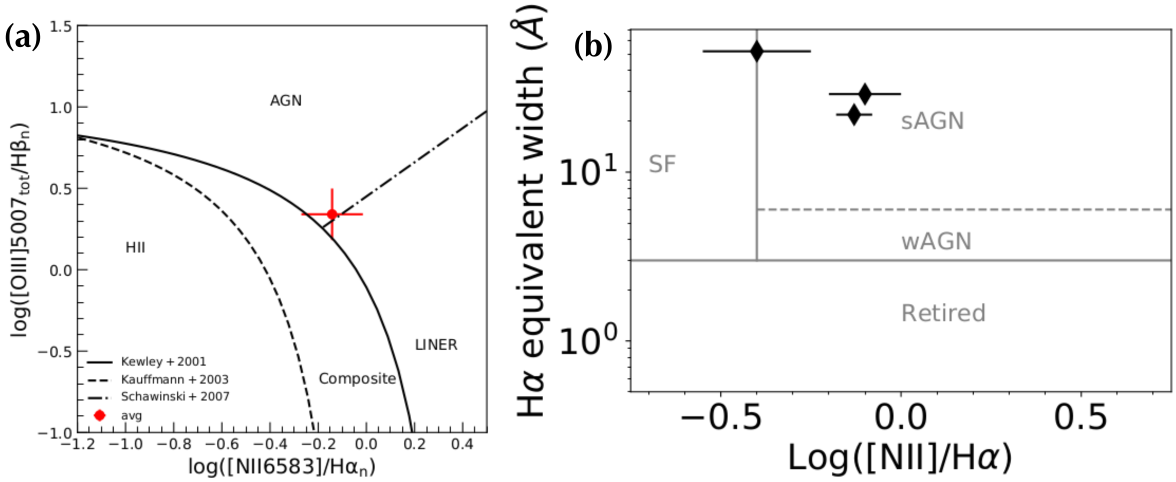

Figure S3: **ASASSN-20qc's BPT and WHAN diagnostic diagrams.** **(a)** ASASSN-20qc's position in the BPT diagram is based on the weighted average of all the reliable measurements. The Kewley et al. (2001) (*73*), Kauffmann et al. (2003) (*74*), and Schawinski et al. (2007) (*75*) separation line of LINER and AGNs are also shown. **(b)** WHAN diagram showing the strong AGN (sAGN), weak AGN (wAGN), star formation (SF) and retired galaxies (see (*72*) for definitions). The 3 data points correspond to the 3 optical spectra with well-constrained line measurements. Both the BPT and the WHAN diagrams indicate that ASASSN-20qc hosts an AGN.

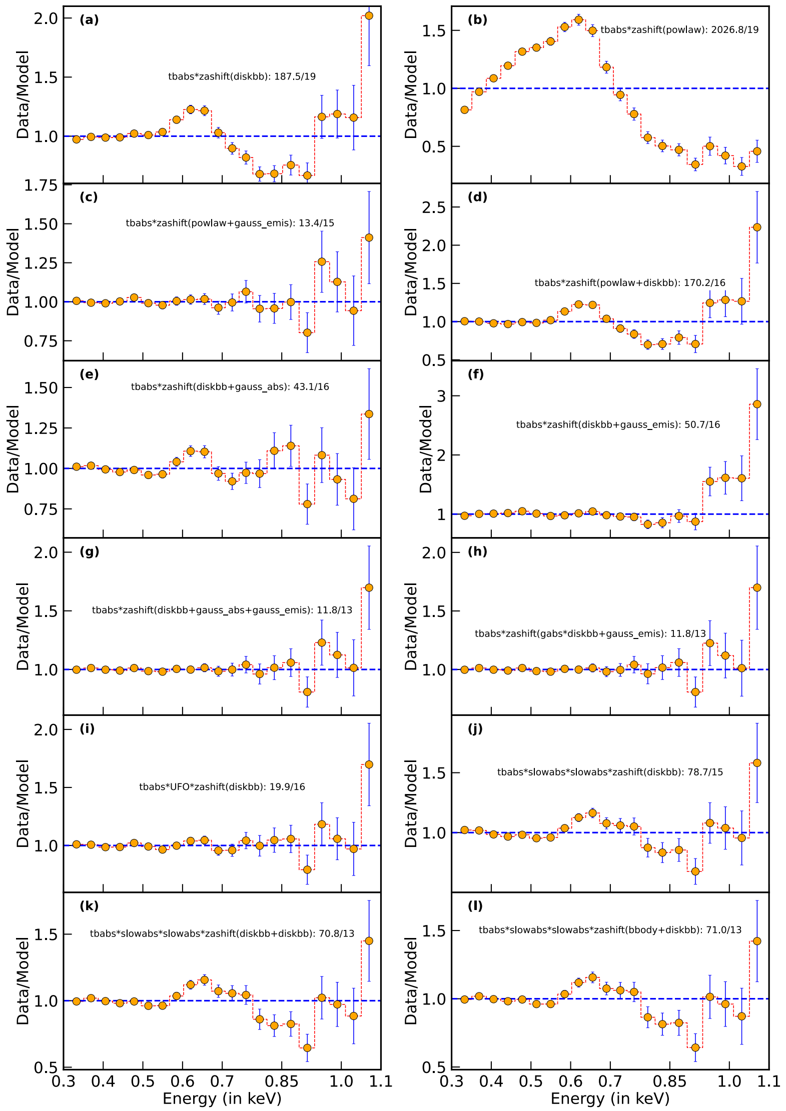

Figure S4: **Ratio of the observed X-ray spectrum over the best-fit model.** EPIC-pn's 0.3-1.1 keV energy spectrum was modeled with various phenomenological models. Each panel shows the ratio: Data/best-fitting model. The models are: (a) thermal accretion disk; (b) powerlaw; (c) powerlaw plus a Gaussian line; (d) powerlaw plus a thermal disk; (e) thermal disk plus inverted Gaussian line; (f) a disk plus a Gaussian emission line; (g) thermal disk plus an inverted Gaussian plus a Gaussian line; (h) Gaussian absorption line modifying the thermal disk plus a Gaussian line; (i) ionized absorber; (j) two warm absorbers from RGS and a disk; (k) two warm absorbers and two disks; (l) two warm absorbers from RGS, a disk and a blackbody component. The exact model used in *XSPEC* is shown on each panel. See Methods section 5 for a detailed description of the spectral fits. The errorbars represent 1-σ uncertainties.

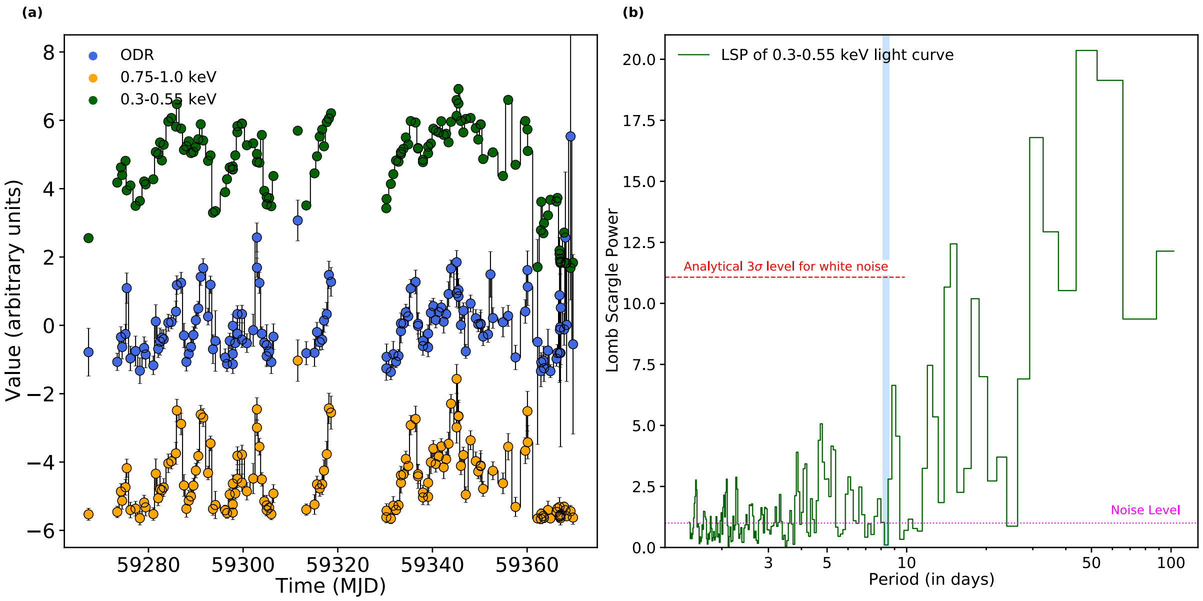

Figure S5: **Background-subtracted soft (0.3–0.55 keV), hard (0.75–1 keV) and ODR curves and LSP of the 0.3-0.55 keV light curve**. **(a)** The 0.3-0.55 keV represents the continuum flux while the 0.75-1.0 keV tracks the outflow's strength. **(b)** The LSP of the continuum is dominated by red noise and does not show a statistically significant peak near 8.5 days (blue shaded column).

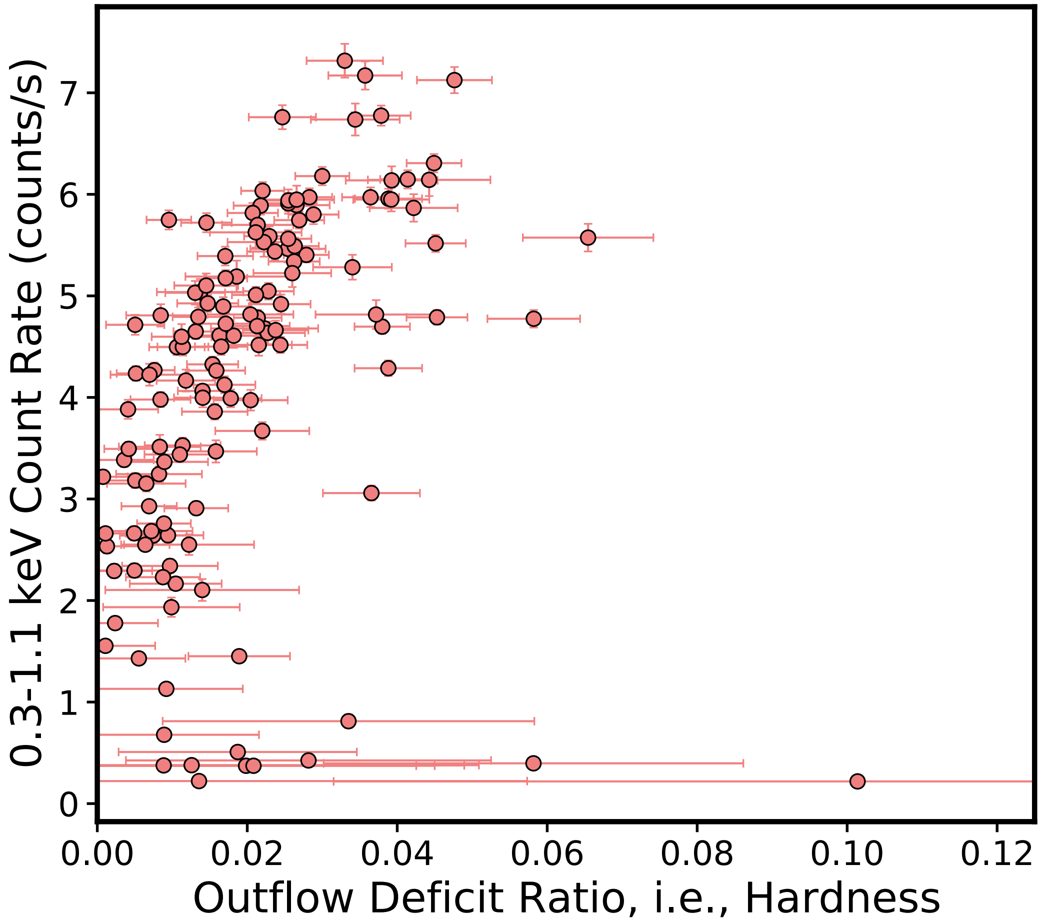

Figure S6: **ODR vs 0.3-1.1 keV count rate**. ODR represents the strength of the broad absorption line with respect to the continuum and the 0.3-1.1 keV count rate represents the overall X-ray intensity. A lower ODR value indicates stronger absorption dip and stronger outflow.

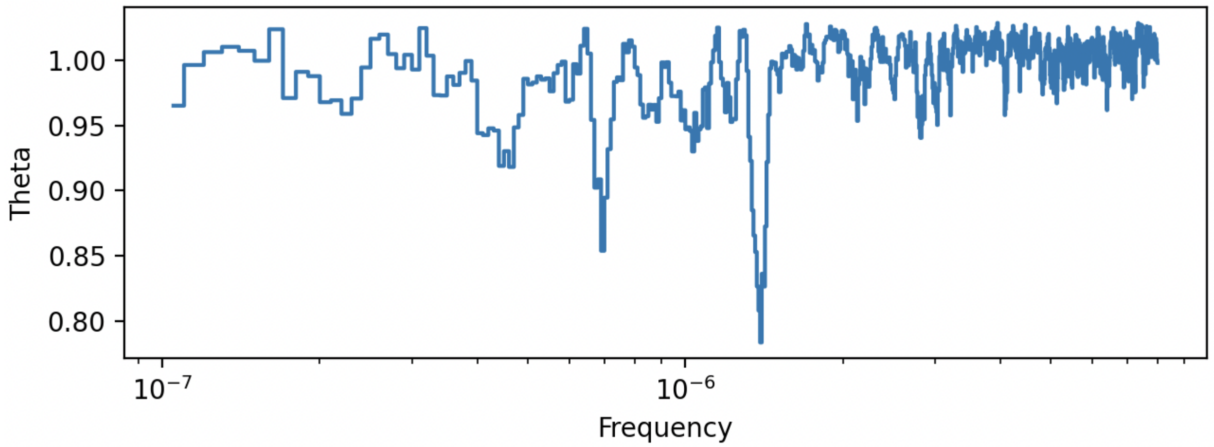

Figure S7: **Results from application of the phase dispersion minimization algorithm on the ODR time series**. The strongest dip is at 1.4×10^{-6} Hz (8.5 days), which is consistent with the LSP analysis.

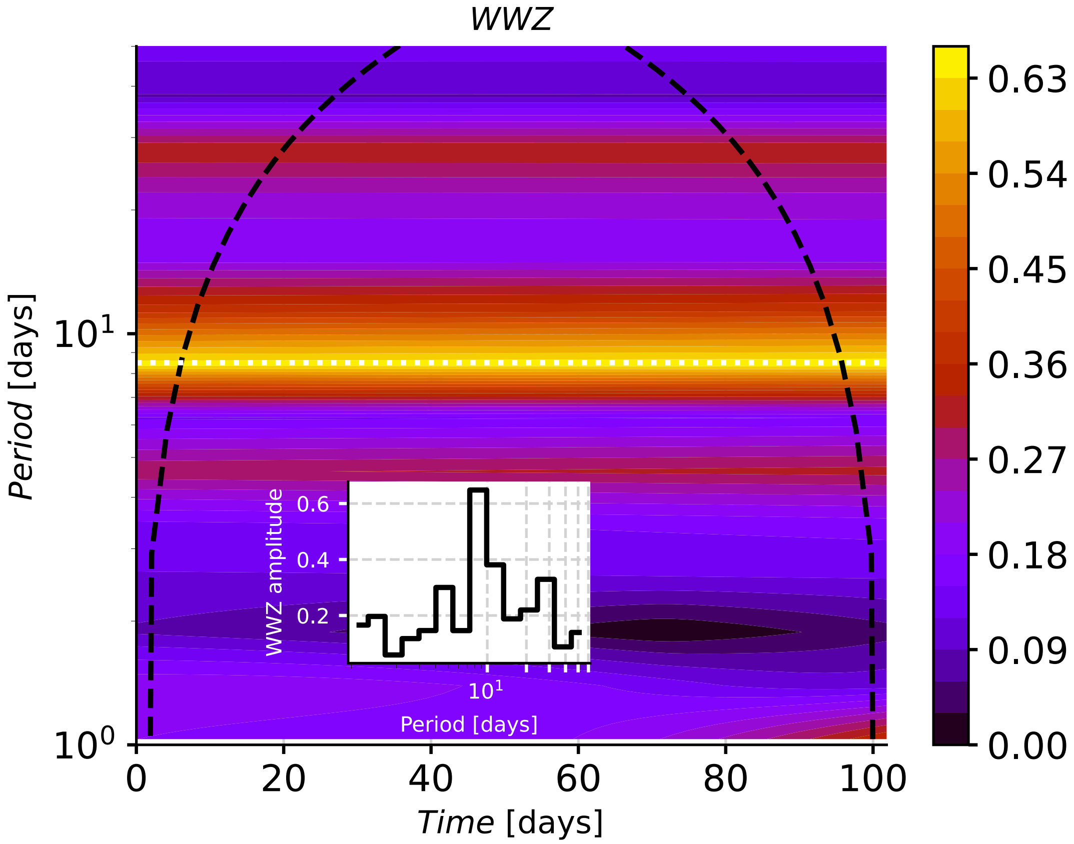

Figure S8: **ODR periodicity analysis using Weighted Wavelet Z-transform (WWZ)**. The plot depicts the colour-coded WWZ amplitude in the time–period plane (both in days). The dotted white line is at 8.5 days, which is consistent with the LSP peak within the uncertainties for the whole time range. The figure inset depicts the WWZ amplitude vs. period (in days).

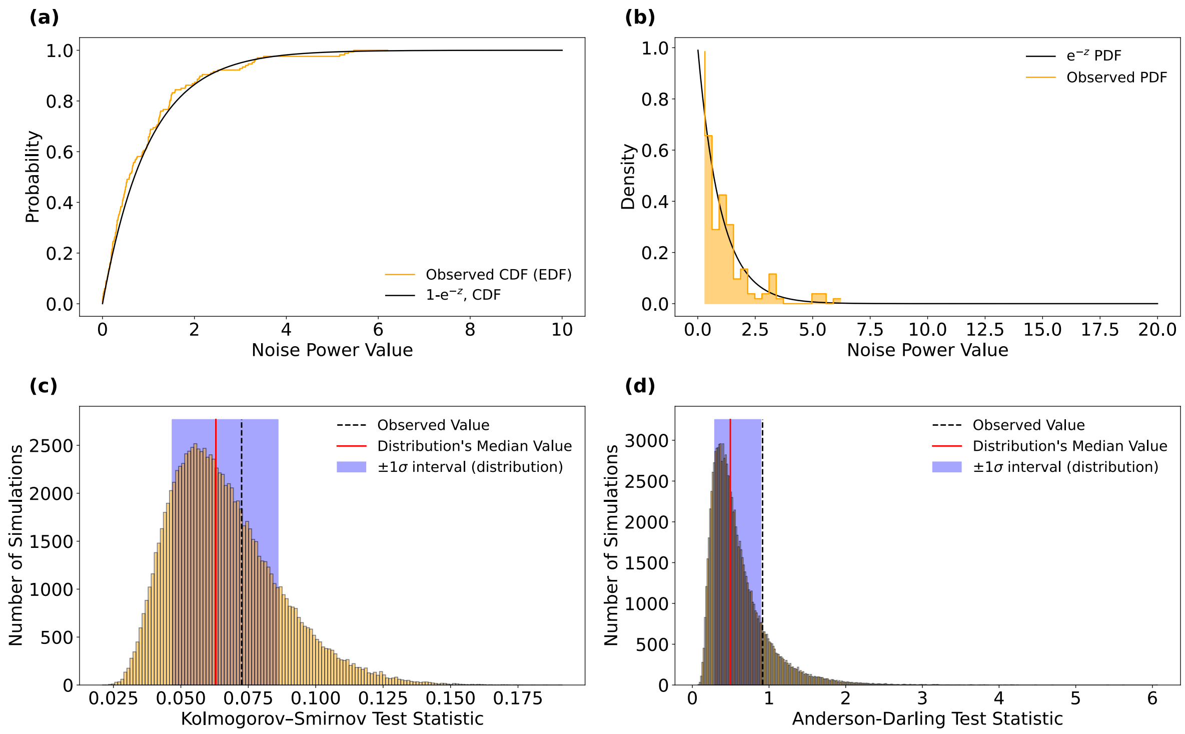

Figure S9: **White noise tests for the distribution of noise powers in the observed Lomb Scargle periodogram. (a) Comparison of the cumulative distribution functions (CDFs) of the observed noise powers and the expected exponential distribution.** The orange histogram is the CDF of the observed Lomb Scargle Periodogram of the ODR curve while the solid black line shows the CDF of white noise powers, i.e., the exponential distribution. **(b) Comparison of the probability density functions of the observed noise powers and the expected exponential distribution.** The shaded orange histogram represents the PDF of the noise powers in the observed LSP of ODR curve while the solid black line is the PDF of white noise distribution, i.e., the exponential distribution. **(c) Distribution of the Kolmogorov-Smirnov test statistic derived from simulations**. The solid red and the dashed black lines represent the median of the distribution and the observed value, respectively. **(d) Distribution of the Anderson-Darling test statistic using simulations**. The solid red and the dashed black lines represent the median of the distribution and the observed value, respectively. In both **(c)** and **(d)** the shaded blue regions indicate the $\pm 1\sigma$ values of their respective distributions.

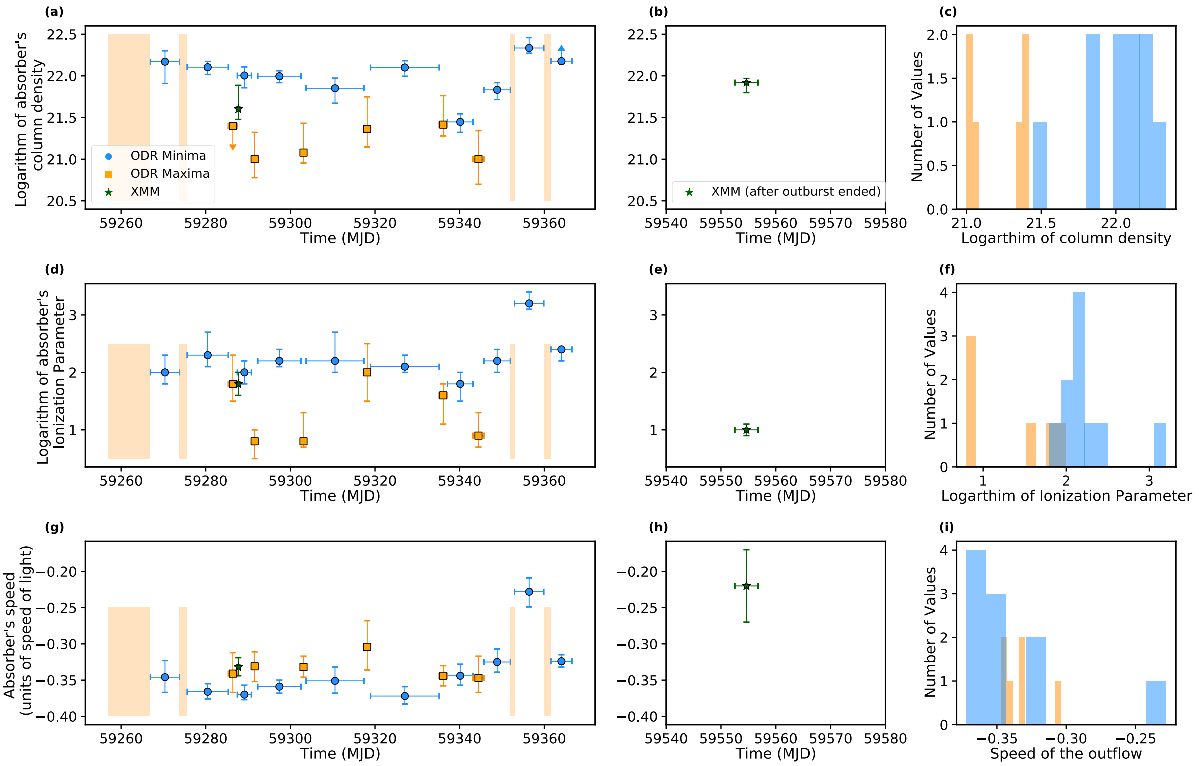

Figure S10: **Properties of the Quasi-Periodic Outflow (QPOuts) and their time evolution. (a), (b) Logarithm of the absorbing column density of the outflow vs time.** The blue filled circles and the orange squares represent data from the ODR minima and maxima, respectively. The shaded orange regions represent the epochs of ODR maxima where an outflow was not detected in the energy spectra. Data from *XMM-Newton* are shown as green stars. **(c) Histogram of the logarithm of outflow's column density during ODR maxima and minima.** Blue and orange histograms represent the data from ODR minima and maxima, respectively. The outflow has an order of magnitude higher column during the ODR minima compared to the maxima. **(d), (e). Same as (a) and (b) but here the evolution of the logarithm of the ionization parameter of the outflow is shown. (f) Histogram of the logarithm of the ionization parameter during the ODR maxima and minima.** Same color scheme as (c). The ionization parameter is roughly an order of magnitude larger during the ODR minima compared to ODR maxima. **(g), (h) Evolution of the outflow's line of sight speed with time.** Same color scheme as in (a) and (b). **(i) Histogram of the outflow speed during ODR maxima and minima.** The outflow speed is consistent between the ODR maxima and minima.

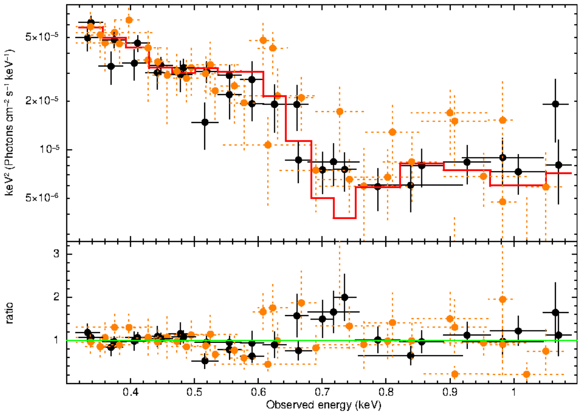

Figure S11: *XMM-Newton* **EPIC spectra at late times (after the initial outburst ended).** The black and the orange data points represent the pn and the combined MOS spectra, respectively. The spectra are combined and rebinned for visual purpose only. The red histogram is the best-fit model containing the outflow.

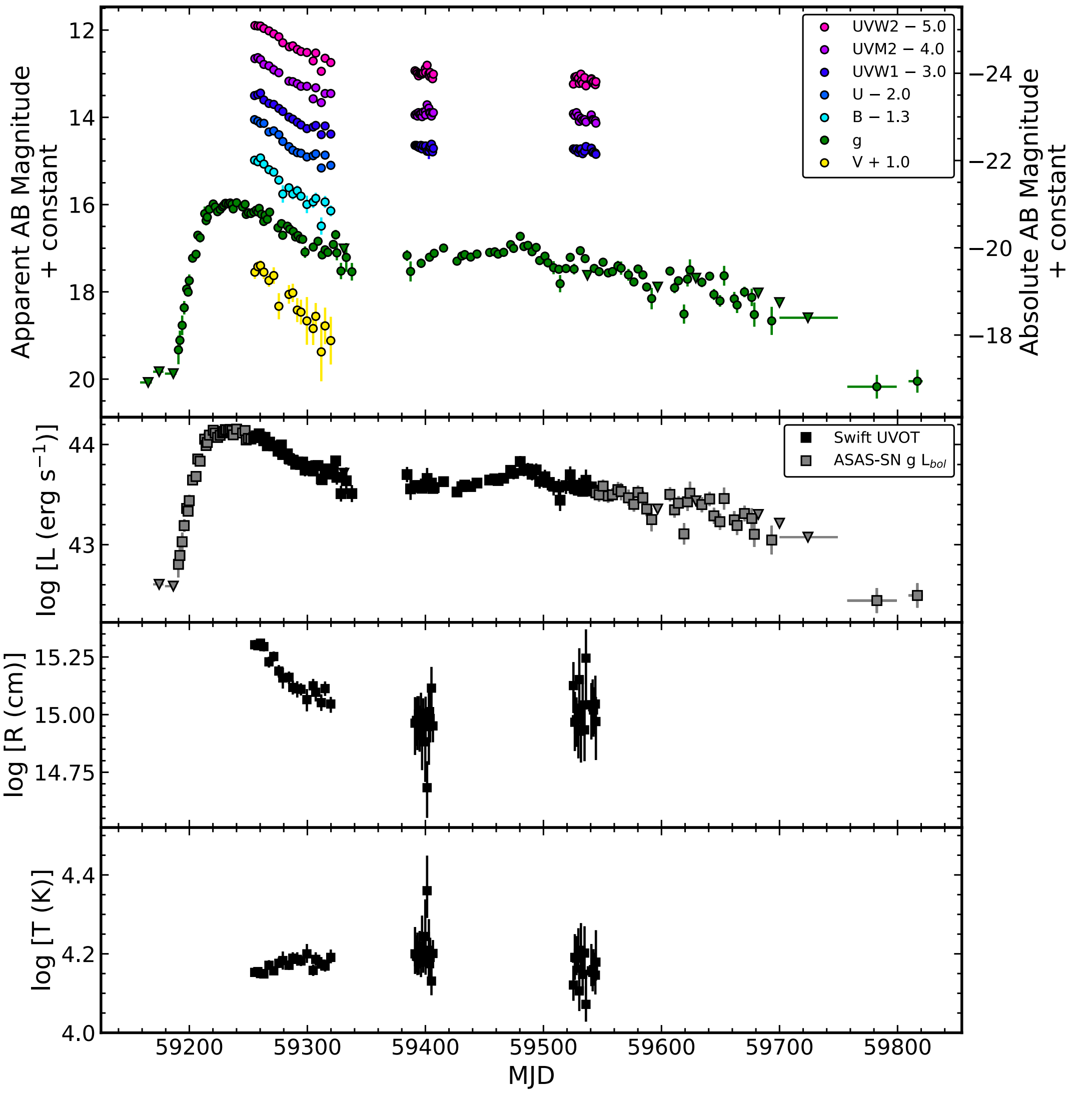

Figure S12: **Host-subtracted and Galactic extinction-corrected UV/optical light curves of ASASSN-20qc (top panel) from the Swift UVOT (UV+UBV) and ASAS-SN (g).** Evolution of the UV/optical blackbody luminosity (second panel), radius (third panel), and temperature (bottom panel) for ASASSN-20qc. The gray squares indicate where ASAS-SN g-band data outside the temporal range covered by Swift has been bolometrically corrected using nearby Swift data. The ASAS-SN data has been stacked in 10-day bins prior to the flare for deep limits, 1-day bins during the rise and peak of the flare, 3-day bins after the seasonal break, and 50-day bins for the final deeper points.

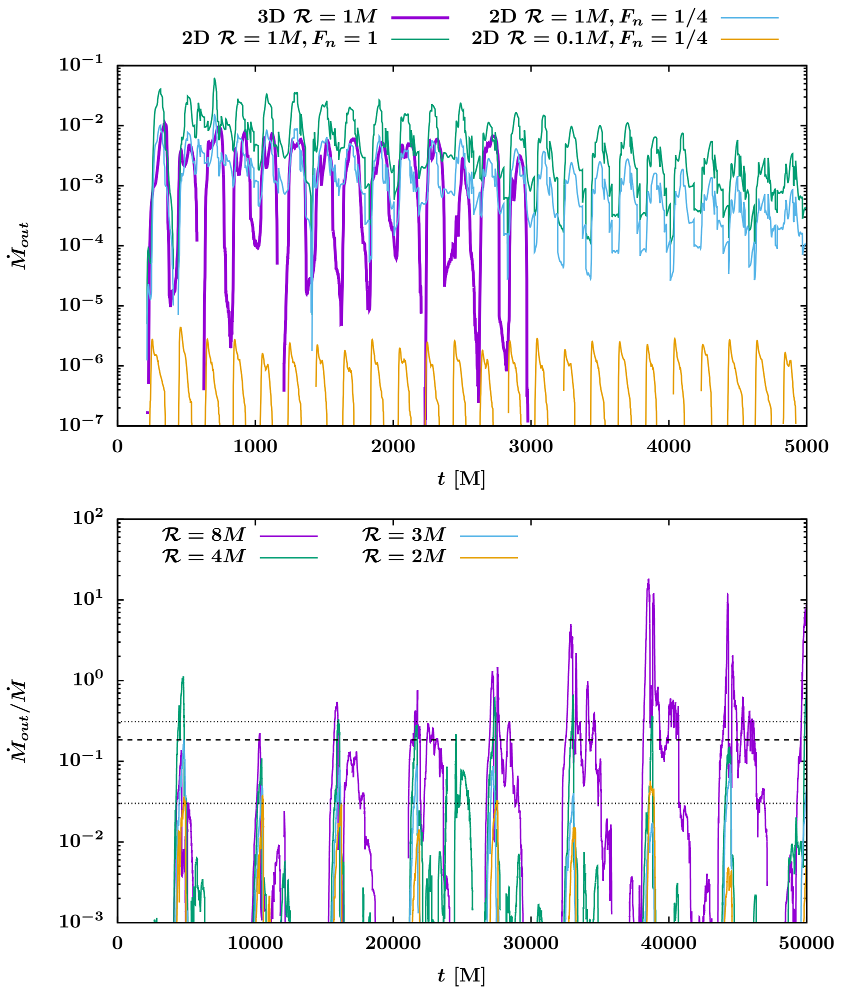

Figure S13: **Constraining the influence radius of the perturber using the ratio of the outflow rate to the inflow rate. Top panel:** Outflow rate $\dot{M}_{\rm out}$ with $v > 0.5c$ for the simulation corresponding to a perturber on a circular inclined orbit with $r = 10M$. The purple line shows the result from the 3D GRMHD simulation with a perturber influence radius of $\mathcal{R} = 1M$ (Run 9) while the green line shows the result from the 2D GRMHD simulation scaled by $\frac{2\mathcal{R}}{(2\pi r)}F_n$ with $\mathcal{R} = 1M$, $F_n = 1$ (Run 10). The blue line shows results from a 2D run with $\mathcal{R} = 1M$, $F_n = 1/4$ (Run 10), and align better with the 3D results. The orange line depicts a 2D run with low perturber radius of $\mathcal{R} = 0.1M$ and $F_n = 1/4$ (Run 11), and highlights the >3 orders of magnitude weaker outflow compared to $\mathcal{R} = 1M$. **Bottom panel:** The ratio of the outflow to the inflow rate in simulations of ASASSN-20qc with different $\mathcal{R}$ (Runs 2, 6, 7 and 8). The long-dashed line represents the average of observationally inferred ratio $\dot{M}_{out}/\dot{M}_{acc}$ during *NICER* ODR Min phases (0.184), while the short-dashed lines represent the minimal (0.03) and maximal values (0.31) of the ratio, see Table S6.

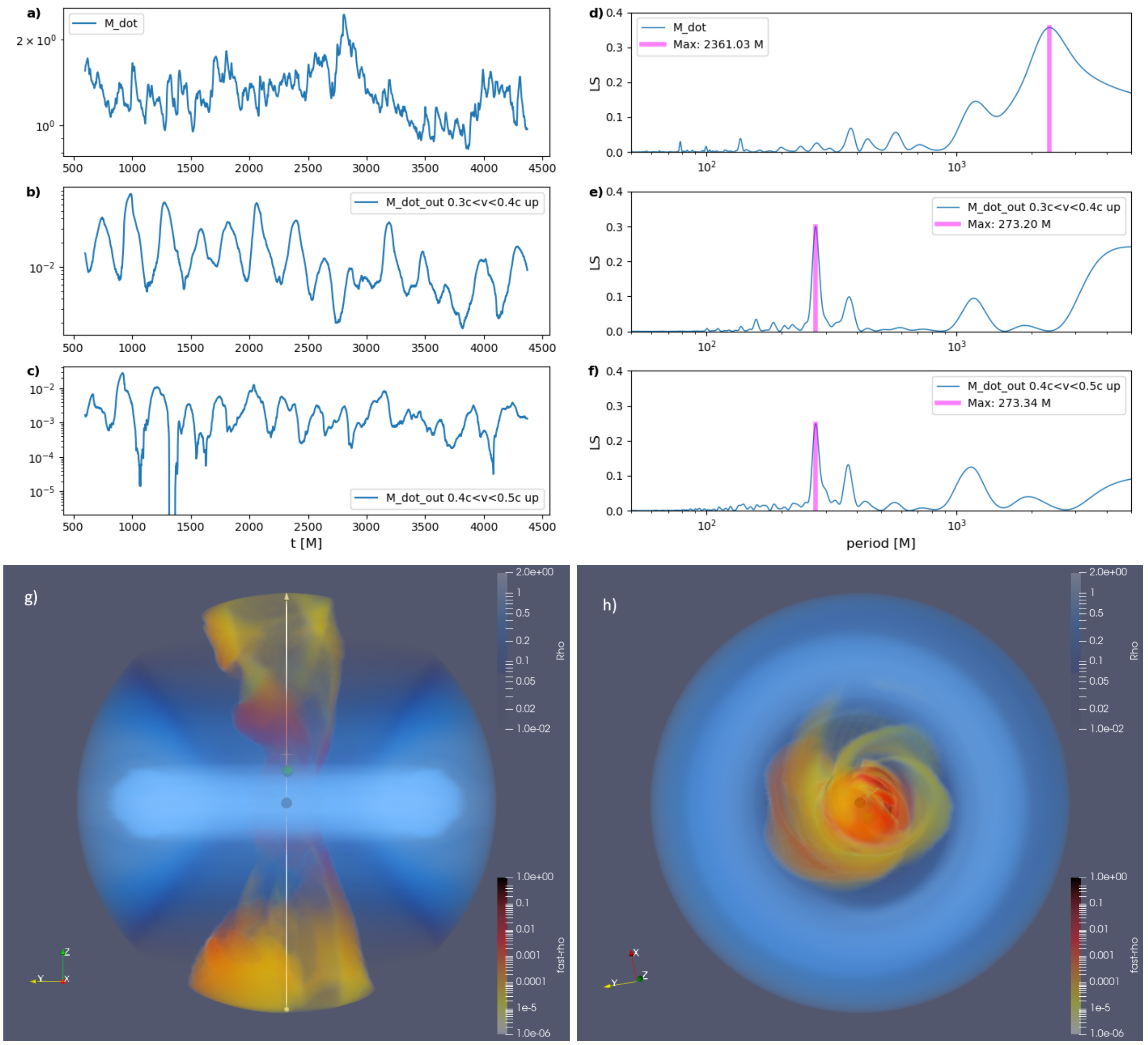

Figure S14: **Run 16: a) accretion rate $\dot{\mathcal{M}}(t)$, b) outflow rate $\dot{\mathcal{M}}_{\rm out-up}(t)$ with $0.3c < v < 0.4c$, c) outflow rate $\dot{\mathcal{M}}_{\rm out-up}(t)$ with $0.4c < v < 0.5c$, d) - f) Lomb-Scargle periodogram of a) - c). The initial transient time $t_t = 600M$ was omitted from the analysis. g) - h) snapshots from Run 16 showing the density of the flow. The blue color scale shows the slowly moving matter, while the yellow-red color scale shows the fast-outflowing gas. The pertuber is shown by green color. This 3D movie is available at https://youtu.be/fwgUEzGpApU.**

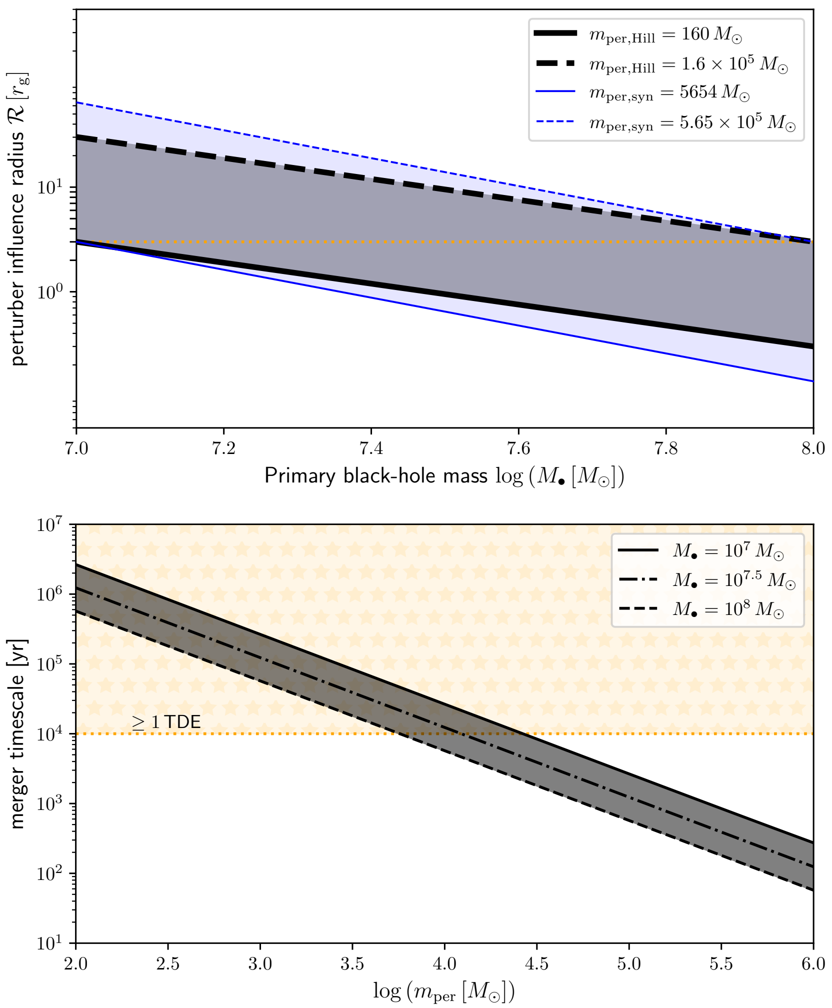

Figure S15: **Top: The influence radius of the perturber expressed as a function of the primary SMBH mass.** The calculations are performed for the limiting lower and upper masses using the tidal (Hill) radius relation, Eq. (S12), and the synchronization-radius relation, Eq. (S13), respectively, and the adopted rest-frame orbital period is 8.05 days. The horizontal dotted orange line stands for the influence radius of $\mathcal{R} = 3r_{\rm g}$, which is preferred based on the comparison of GRMHD simulations with the inferred outflow/inflow rate ratio during ODR minima. **Bottom: Merger timescale for the two-body system SMBH-IMBH with the rest-frame orbital period of** 8.05 **days as a function of the IMBH mass.** We depict the merger timescales for the three cases of the primary SMBH mass, $10^7\,M_\odot$, $10^{7.5}\,M_\odot$, and $10^8\,M_\odot$, using the solid, dash-dotted, and the dashed lines, respectively. The horizontal orange dotted line marks the timescale of 10^4 years, which is considered to be the typical minimum timescale for the tidal disruption event to take place per galaxy. The IMBH masses, for which the merger timescale is greater than this TDE timescale ($m_{\rm per} \lesssim 10^4\,M_\odot$), are favoured due to an increased chance of the simultaneous SMBH-IMBH inspiral and the TDE.

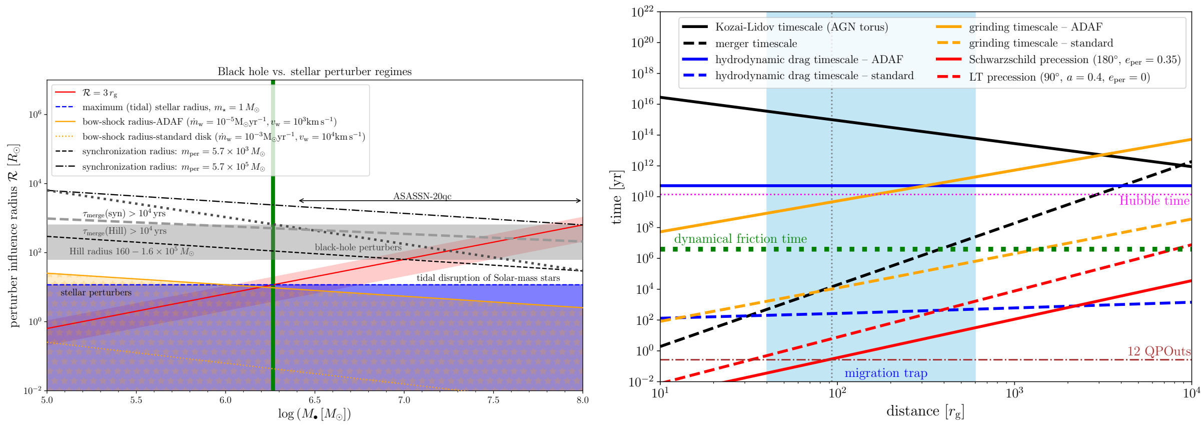

Figure S16: **Left: Influence radius of a disk perturber expressed in Solar radii as a function of the SMBH mass.** For lighter SMBHs of $\lesssim 10^{6.27}\,M_\odot$ (vertical green line), perturbers can be stars due to large enough relative cross-sections (physical or due to a strong stellar wind), while for heavier SMBHs, to which ASASSN-20qc SMBH belongs, stellar-mass and intermediate-mass black holes will yield large enough spheres of gravitational influence while tidally stable stars will remain significantly below the limiting value of three gravitational radii (solid red line). The red shaded area expresses influence radii in the range of $\mathcal{R} = 1 - 5\,r_g$. The dotted black line marks the limit where $\tau_{merge} = 10^4$ years for the influence radius given by the synchronization radius, while the dashed gray line represents the same limit when the influence radius is calculated using the Hill expression. **Right: Dependency of dynamical timescales on the distance of the IMBH from the SMBH.** We show the radial dependency of the Kozai-Lidov timescale for the AGN torus perturbation, the merger timescale for the SMBH-IMBH pair, hydrodynamical drag and grinding timescales for both the ADAF and the standard disk cases; see the legend. The solid and dashed red lines represent general relativistic Schwarzschild and Lense-Thirring precession timescales, respectively. The shaded blue rectangle represents an approximate location of migration traps within the accretion disk ($\sim 40 - 600\,r_g$ according to (*165*)). The horizontal dotted green line stands for the dynamical friction time as given by Eq. (S19). Dashed vertical gray line depicts the orbital distance of the considered IMBH perturber orbiting around $10^{7.4}\,M_\odot$ SMBH once in every 8.5 days. The dash-dotted brown horizontal line represents the duration of 12 QPOuts (96.6 days in the source frame). The dotted horizontal magenta line shows the Hubble time. For all the timescale radial profiles, the IMBH mass is set to $10^4\,M_\odot$ and the SMBH mass to $10^{7.4}\,M_\odot$ when relevant.

Supplement Movie Caption S1: General-relativistic magnetohydrodynamical (GRMHD) simulation of repetitive stellar transits through the accretion flow onto a supermassive black hole with $10^{7.4}$ $M_\odot$ and $a = 0.4$. The perturbing companion is consistent with an intermediate-mass black hole of $\gtrsim 100\,M_\odot$, which is moving along an eccentric geodesics in Kerr spacetime with r = 93 M. The influence radius of the perturber is $\mathcal{R} = 3M$ and is shown to scale. The simulation was performed using the GRMHD code HARM, with the observed orbital period of the perturber set to 8.5 days. The simulation is run in 2D, where the phi-coordinate of the star position is not taken into account. **Top panels, from the left to the right: (a) Spatial distribution of the logarithm of mass density.** The horizontal and the vertical axes are spatial coordinates expressed in gravitational radii. The white contours indicate the magnetic field configuration. The position and size of the perturber is shown by the black circle, while the grey line displays its trajectory in the 2D slice. **(b) Spatial distribution of the Lorentz factor of the gas bulk motion; (c) Spatial distribution of the mass-outflow rate with $v > 0.2c$.** The outflow rate is colour-coded using arbitrary units according to the colour-bar to the right. **Bottom panel: Temporal profiles of the inflow rate (blue), the outflow rate through the upper funnel (purple), and the outflow rate through the lower funnel (green).** The inflow and the outflow rates are expressed in arbitrary units. The time is expressed in days in the observed frame measured from the moment, when the perturber was launched into the flow. The coloured points indicate the actual inflow and the outflow rates. YouTube link to the movie: https://youtu.be/WQmS8h3Zzeo.

REFERENCES AND NOTES

1. K. Z. Stanek, ASAS-SN transient discovery report for 2020-12-20. *Transient Name Serv. Disc. Rep.* **2020-3850**, 1 (2020).

2. C. S. Kochanek, B. J. Shappee, K. Z. Stanek, T. W.S. Holoien, T. A. Thompson, J. L. Prieto, S. Dong, J. V. Shields, D. Will, C. Britt, D. Perzanowski, G. Pojmański, The all-sky automated survey for supernovae (ASAS-SN) light curve server v1.0. *Publ. Astron. Soc. Pac.* **129**, 104502 (2017).

3. B. J. Shappee, J. L. Prieto, D. Grupe, C. S. Kochanek, K. Z. Stanek, G. de Rosa, S. Mathur, Y. Zu, B. M. Peterson, R. W. Pogge, S. Komossa, M. Im, J. Jencson, T.W.S. Holoien, U. Basu, J. F. Beacom, D. M. Szczygieł, J. Brimacombe, S. Adams, A. Campillay, C. Choi, C. Contreras, M. Dietrich, M. Dubberley, M. Elphick, S. Foale, M. Giustini, C. Gonzalez, E. Hawkins, D. A. Howell, E. Y. Hsiao, M. Koss, K. M. Leighly, N. Morrell, D. Mudd, D. Mullins, J. M. Nugent, J. Parrent, M. M. Phillips, G. Pojmanski, W. Rosing, R. Ross, D. Sand, D. M. Terndrup, S. Valenti, Z. Walker, Y. Yoon, The man behind the curtain: X-rays drive the UV through NIR variability in the 2013 active galactic nucleus outburst in NGC 2617. *Astrophys. J.* **788**, 48 (2014).

4. I. Arcavi, C. Pellegrino, J. Burke, D. Hiramatsu, A. Howell, C. McCully, E. P. Gonzalez, StarDestroyers transient classification report for 2021-01-12. *Transient Name Ser. Classif. Rep.* **2021-121**, 1 (2021).

5. F. Tombesi, M. Meléndez, S. Veilleux, J. N. Reeves, E. González-Alfonso, C. S. Reynolds, Wind from the black-hole accretion disk driving a molecular outflow in an active galaxy. *Nature* **519**, 436–438 (2015).

6. J. D. Scargle, Studies in astronomical time series analysis. II. Statistical aspects of spectral analysis of unevenly spaced data. *Astrophys. J.* **263**, 835 (1982).

7. J. H. Horne, S. L. Baliunas, A prescription for period analysis of unevenly sampled time series. *Astrophys. J.* **302**, 757 (1986).

8. P. Sukova, M. Zajacek, V. Witzany, V. Karas, Stellar transits across a magnetized accretion torus as a mechanism for plasmoid ejection. *Astrophys. J.* **917**, 43 (2021).

9. O. Semerak, V. Karas, F. de Felice, Parameters of black holes in sources with periodic variability. *Publ. Astron. Soc. Japan* **51**, 571– 577 (1999).

10. A. Tchekhovskoy, J. C. McKinney, R. Narayan, WHAM: A WENO-based general relativistic numerical scheme—I. Hydrodynamics. *Mon. Not. R. Astron. Soc.* **379**, 469–497 (2007).

11. S. M. Ressler, A. Tchekhovskoy, E. Quataert, M. Chandra, C. F. Gammie, Electron thermodynamics in GRMHD simulations of low-luminosity black hole accretion. *Mon. Not. R. Astron. Soc.* **454**, 1848–1870 (2015).

12. C. F. Gammie, J. C. McKinney, G. Toth, HARM: A numerical scheme for general relativistic magnetohydrodynamics. *Astrophys. J.* **589**, 444– 457 (2003).

13. S. C. Noble, C. F. Gammie, J. C. McKinney, L. Del Zanna, Primitive variable solvers for conservative general relativistic magnetohydrodynamics. *Astrophys. J.* **641**, 626– 637 (2006).

14. F. Yuan, R. Narayan, Hot accretion flows around black holes. *Annu. Rev. Astron. Astrophys.* **52**, 529– 588 (2014).

15. M. J. Rees, Tidal disruption of stars by black holes of 106-108 solar masses in nearby galaxies. *Nature* **333**, 523– 528 (1988).

16. S. van Velzen, S. Gezari, E. Hammerstein, N. Roth, S. Frederick, C. Ward, T. Hung, S. B. Cenko, R. Stein, D. A. Perley, K. Taggart, R. J. Foley, J. Sollerman, N. Blagorodnova, I. Andreoni, E. C. Bellm, V. Brinnel, K. de, R. Dekany, M. Feeney, C. Fremling, M. Giomi, V. Z.

Golkhou, M. J. Graham, A. Y. Q. Ho, M. M. Kasliwal, C. D. Kilpatrick, S. R. Kulkarni, T. Kupfer, R. R. Laher, A. Mahabal, F. J. Masci, A. A. Miller, J. Nordin, R. Riddle, B. Rusholme, J. . Santen, Y. Sharma, D. L. Shupe, M. T. Soumagnac, Seventeen tidal disruption events from the first half of ZTF survey observations: Entering a new era of population studies. *Astrophys. J.* **908**, 4 (2021).

17. J. T. Hinkle, T. W. S. Holoien, B. J. Shappee, K. Auchettl, A Swift fix for nuclear outbursts. *Astrophys. J.* **910**, 83 (2021).

18. D. R. Pasham, S. B. Cenko, A. Sadowski, J. Guillochon, N. C. Stone, S. . Velzen, J. K. Cannizzo, Optical/UV-to-X-ray echoes from the tidal disruption flare ASASSN-14li. *Astrophys. J.* **837**, L30 (2017).

19. S. Gezari, S. B. Cenko, I. Arcavi, X-ray brightening and UV fading of tidal disruption event ASASSN-15oi. *Astrophys. J.* **851**, L47 (2017).

20. N. C. Stone, E. Vasiliev, M. Kesden, E. M. Rossi, H. B. Perets, P. Amaro-Seoane, Rates of stellar tidal disruption. *Space Sci. Rev.* **216**, 35 (2020).

21. A. Ingram, C. Done, P. C. Fragile, Low-frequency quasi-periodic oscillations spectra and Lense-Thirring precession. *Mon. Not. R. Astron. Soc.* **397**, L101–L105 (2009).

22. K. Fukumura, F. Tombesi, D Kazanas, C. Shrader, E. Behar, I. Contopoulos, Stratified magnetically driven accretion-disk winds and their relations to jets. *Astrophys. J.* **780**, 120 (2014).

23. K. Fukumura, D. Kazanas, I. Contopoulos, E. Behar, Magnetohydrodynamic accretion disk winds as x-ray absorbers in active galactic nuclei. *Astrophys. J.* **715**, 636 (2010), 650.

24. M. Nomura, K. Ohsuga, H. R. Takahashi, K. Wada, T. Yoshida, Radiation hydrodynamic simulations of line-driven disk winds for ultra-fast outflows. *Publ. Astronom. Soc. Japan* **68** (2016). 16.

25. M. Elvis, A structure for quasars. *Astrophys. J.* **545**, 63 (2000).

26. A. King, K. Pounds, Powerful outflows and feedback from active galactic nuclei. *Annu. Rev. Astron. Astrophys.* **53**, 115- 154 (2015).

27. C. S. Reynolds, Measuring black hole spin using x-ray reflection spectroscopy. *Space Sci. Rev.* **183**, 277–294 (2014).

28. M. Masterson, E. Kara, C. Ricci, J. A. García, A. C. Fabian, C. Pinto, P. Kosec, R. A. Remillard, M. Loewenstein, B. Trakhtenbrot, I. Arcavi, Evolution of a relativistic outflow and x-ray corona in the extreme changing-look AGN 1ES 1927+654. *Astrophys. J.* **934**, 35 (2022).

29. B. Ripperda, M. Liska, K. Chatterjee, G. Musoke, A. A. Philippov, S. B. Markoff, A. Tchekhovskoy, Z. Younsi, Black hole flares: Ejection of accreted magnetic flux through 3D plasmoid-mediated reconnection. *Astrophys. J.* **924**, L32 (2022).

30. A. V. Payne, B. J. Shappee, J. T. Hinkle, P. J. Vallely, C. S. Kochanek, T. W.-S. Holoien, K. Auchettl, K. Z. Stanek, T. A. Thompson, J. M. M. Neustadt, M. A. Tucker, J. D. Armstrong, J. Brimacombe, P. Cacella, R. Cornect, L. Denneau, M. M. Fausnaugh, H. Flewelling, D. Grupe, A. N. Heinze, L. A. Lopez, B. Monard, J. L. Prieto, A. C. Schneider, S. S. Sheppard, J. L. Tonry, H. Weiland, ASASSN-14ko is a periodic nuclear transient in ESO 253-G003. *Astrophys. J.* **910**, 125 (2021).

31. T. Wevers, E. R. Coughlin, D. R. Pasham, M. Guolo, Y. Sun, S. Wen, P. G. Jonker, A. Zabludoff, A. Malyali, R. Arcodia, Z. Liu, A. Merloni, A. Rau, I. Grotova, P. Short, Z. Cao, Live

to die another day: The rebrightening of AT2018fyk as a repeating partial tidal disruption event. arXiv:2209.07538 [astro-ph.HE] (2022).

32. Z. Liu, A. Malyali, M. Krumpe, D. Homan, A. J. Goodwin, I. Grotova, A. Kawka, A. Rau, A. Merloni, G. E. Anderson, J. C. A. Miller-Jones, A. G. Markowitz, S. Ciroi, F. Di Mille, M. Schramm, S. Tang, D. A. H. Buckley, M. Gromadzki, C. Jin, and J. Buchner, Deciphering the extreme X-ray variability of the nuclear transient eRASSt J045650.3–203750: A likely repeating partial tidal disruption event. *Astron. Astrophy.* **669**, A75 (2023).

33. M. Cufari, E. R. Coughlin, C. J. Nixon, Using the hills mechanism to generate repeating partial tidal disruption events and ASASSN-14ko. *Astrophys. J. Lett.* **929**, L20 (2022).

34. A. Janiuk, B. Czerny, A. Siemiginowska, Radiation pressure instability as a variability mechanism in the microquasar GRS 1915+105. *Astrophys. J.* **542**, L33 (2000), L36.

35. M. Sniegowska, M. Grze Dzielski, B. Czerny, A. Janiuk, Modified models of radiation pressure instability applied to 10, 105, and 107M⊙accreting black holes. *Astron. Astrophys.* **672**, A19 (2023).

36. J. E. McClintock, R. A. Remillard, *Black hole binaries*. arXiv:astro-ph/0306213 (2003).

37. G. Fragione, Mergers of supermassive and intermediate-mass black holes in galactic nuclei from disruptions of star clusters. *Astrophys. J.* **939**, 97 (2022).

38. Planck Collaboration, N. Aghanim, Y. Akrami, M. Ashdown, J. Aumont, C. Baccigalupi, M. Ballardini, A. J. Banday, R. B. Barreiro, N. Bartolo, S. Basak, R. Battye, K. Benabed, J.-P. Bernard, M. Bersanelli, P. Bielewicz, J. J. Bock, J. R. Bond, J. Borrill, F. R. Bouchet, F. Boulanger, M. Bucher, C. Burigana, R. C. Butler, E. Calabrese, J.-F. Cardoso, J. Carron, A. Challinor, H. C. Chiang, J. Chluba, L. P. L. Colombo, C. Combet, D. Contreras, B. P. Crill, F. Cuttaia, P. de Bernardis, G. de Zotti, J. Delabrouille, J.-M. Delouis, E. Di Valentino, J. M.

Diego, O. Doré, M. Douspis, A. Ducout, X. Dupac, S. Dusini, G. Efstathiou, F. Elsner, T. A. Enßlin, H. K. Eriksen, Y. Fantaye, M. Farhang, J. Fergusson, R. Fernandez-Cobos, F. Finelli, F. Forastieri, M. Frailis, A. A. Fraisse, E. Franceschi, A. Frolov, S. Galeotta, S. Galli, K. Ganga, R. T. Génova-Santos, M. Gerbino, T. Ghosh, J. González-Nuevo, K. M. Górski, S. Gratton, A. Gruppuso, J. E. Gudmundsson, J. Hamann, W. Handley, F. K. Hansen, D. Herranz, S. R. Hildebrandt, E. Hivon, Z. Huang, A. H. Jaffe, W. C. Jones, A. Karakci, E. Keihänen, R. Keskitalo, K. Kiiveri, J. Kim, T. S. Kisner, L. Knox, N. Krachmalnicoff, M. Kunz, H. Kurki-Suonio, G. Lagache, J.-M. Lamarre, A. Lasenby, M. Lattanzi, C. R. Lawrence, M. Le Jeune, P. Lemos, J. Lesgourgues, F. Levrier, A. Lewis, M. Liguori, P. B. Lilje, M. Lilley, V. Lindholm, M. López-Caniego, P. M. Lubin, Y.-Z. Ma, J. F. Macías-Pérez, G. Maggio, D. Maino, N. Mandolesi, A. Mangilli, A. Marcos-Caballero, M. Maris, P. G. Martin, M. Martinelli, E. Martínez-González, S. Matarrese, N. Mauri, J. D. McEwen, P. R. Meinhold, A. Melchiorri, A. Mennella, M. Migliaccio, M. Millea, S. Mitra, M.-A. Miville-Deschênes, D. Molinari, L. Montier, G. Morgante, A. Moss, P. Natoli, H. U. Nørgaard-Nielsen, L. Pagano, D. Paoletti, B. Partridge, G. Patanchon, H. V. Peiris, F. Perrotta, V. Pettorino, F. Piacentini, L. Polastri, G. Polenta, J.-L. Puget, J. P. Rachen, M. Reinecke, M. Remazeilles, A. Renzi, G. Rocha, C. Rosset, G. Roudier, J. A. Rubiño-Martín, B. Ruiz-Granados, L. Salvati, M. Sandri, M. Savelainen, D. Scott, E. P. S. Shellard, C. Sirignano, G. Sirri, L. D. Spencer, R. Sunyaev, A.-S. Suur-Uski, J. A. Tauber, D. Tavagnacco, M. Tenti, L. Toffolatti, M. Tomasi, T. Trombetti, L. Valenziano, J. Valiviita, B. Van Tent, L. Vibert, P. Vielva, F. Villa, N. Vittorio, B. D. Wandelt, I. K. Wehus, M. White, S. D. M. White, A. Zacchei, A. Zonca, Planck 2018 results VI. Cosmological parameters. *Astron. Astrophys.* **641**, A6 (2020).

39. E. L. Wright, A cosmology calculator for the world wide web. *Publl. Astron. Soc. Pac.* **118**, 1711– 1715 (2006).

40. K. C. Gendreau, *SPIE* (2016), vol. 9905 of *Society of Photo-Optical Instrumentation Engineers (SPIE) Conference Series*, p. 99051H.

41. L. Struder, The European photon imaging camera on XMM-Newton: The pn-CCD camera. *Astron. Astrophys.* **365**, L18 (2001).

42. M. J. L. Turner, A. Abbey, M. Arnaud, M. Balasini, M. Barbera, E. Belsole, P. J. Bennie, J. P. Bernard, G. F. Bignami, M. Boer, U. Briel, I. Butler, C. Cara, C. Chabaud, R. Cole, A. Collura, M. Conte, A. Cros, M. Denby, P. Dhez, G. di Coco, J. Dowson, P. Ferrando, S. Ghizzardi, F. Gianotti, C. V. Goodall, L. Gretton, R. G. Griffiths, O. Hainaut, J. F. Hochedez, A. D. Holland, E. Jourdain, E. Kendziorra, A. Lagostina, R. Laine, N. la Palombara, M. Lortholary, D. Lumb, P. Marty, S. Molendi, C. Pigot, E. Poindron, K. A. Pounds, J. N. Reeves, C. Reppin, R. Rothenflug, P. Salvetat, J. L. Sauvageot, D. Schmitt, S. Sembay, A. D. T. Short, J. Spragg, J. Stephen, L. Strüder, A. Tiengo, M. Trifoglio, J. Trümper, S. Vercellone, L. Vigroux, G. Villa, M. J. Ward, S. Whitehead, E. Zonca, The European photon imaging camera on XMM-Newton: The MOS cameras. *Astron. Astrophys.* **365**, L27 (2001), L35.

43. J. W. den Herder, A. C. Brinkman, S. M. Kahn, G. Branduardi-Raymont, K. Thomsen, H. Aarts, M. Audard, J. V. Bixler, A. J. den Boggende, J. Cottam, T. Decker, L. Dubbeldam, C. Erd, H. Goulooze, M. Güdel, P. Guttridge, C. J. Hailey, K. al Janabi, J. S. Kaastra, P. A. J. de Korte, B. J. van Leeuwen, C. Mauche, A. J. McCalden, R. Mewe, A. Naber, F. B. Paerels, J. R. Peterson, A. P. Rasmussen, K. Rees, I. Sakelliou, M. Sako, J. Spodek, M. Stern, T. Tamura, J. Tandy, C. P. de Vries, S. Welch, A. Zehnder, The reflection grating spectrometer on board XMM-Newton. *Astron. Astrophys.* **365**, L7– L17 (2001).

44. N. Gehrels, G. Chincarini, P. Giommi, K.O. Mason, J.A. Nousek, A.A. Wells, N.E. White, S.D. Barthelmy, D.N. Burrows, L.R. Cominsky, K.C. Hurley, F.E. Marshall, P. Meszaros, P.W.A. Roming, L. Angelini, L.M. Barbier, T. Belloni, S. Campana, P.A. Caraveo, M.M. Chester, O. Citterio, T.L. Cline, M.S. Cropper, J.R. Cummings, A.J. Dean, E.D. Feigelson, E.E. Fenimore, D.A. Frail, A.S. Fruchter, G.P. Garmire, K. Gendreau, G. Ghisellini, J. Greiner, J.E. Hill, S.D. Hunsberger, H.A. Krimm, S.R. Kulkarni, P. Kumar, F. Lebrun, N.M. Lloyd-Ronning, C.B. Markwardt, B.J. Mattson, R.F. Mushotzky, J.P. Norris, J. Osborne, B. Paczynski, D.M. Palmer, H.S. Park, A.M. Parsons, J. Paul, M.J. Rees, C.S. Reynolds, J.E. Rhoads, T.P. Sasseen, B.E. Schaefer, A.T. Short, A.P. Smale, I.A. Smith, L. Stella, G. Tagliaferri, T. Takahashi, M. Tashiro, L.K. Townsley, J. Tueller, M.J.L. Turner, M. Vietri, W. Voges, M.J. Ward, R. Willingale, F.M. Zerbi, W.W. Zhang, TheSwiftGamma-Ray Burst Mission. *Astrophys. J.* **611**, 1005 (2004), 1020.

45. D. N. Burrows, J. E. Hill, J. A. Nousek, J. A. Kennea, A. Wells, J. P. Osborne, A. F. Abbey, A. Beardmore, K. Mukerjee, A. D. T. Short, G. Chincarini, S. Campana, O. Citterio, A. Moretti, C. Pagani, G. Tagliaferri, P. Giommi, M. Capalbi, F. Tamburelli, L. Angelini, G. Cusumano, H. W. Bräuninger, W. Burkert, G. D. Hartner, The Swift x-ray telescope. *Space Sci. Rev.* **120**, 165–195 (2005).

46. P. Predehl, R. Andritschke, V. Arefiev, V. Babyshkin, O. Batanov, W. Becker, H. Böhringer, A. Bogomolov, T. Boller, K. Borm, W. Bornemann, H. Bräuninger, M. Brüggen, H. Brunner, M. Brusa, E. Bulbul, M. Buntov, V. Burwitz, W. Burkert, N. Clerc, E. Churazov, D. Coutinho, T. Dauser, K. Dennerl, V. Doroshenko, J. Eder, V. Emberger, T. Eraerds, A. Finoguenov, M. Freyberg, P. Friedrich, S. Friedrich, M. Fürmetz, A. Georgakakis, M. Gilfanov, S. Granato, C. Grossberger, A. Gueguen, P. Gureev, F. Haberl, O. Hälker, G. Hartner, G. Hasinger, H. Huber,

L. Ji, A. V. Kienlin, W. Kink, F. Korotkov, I. Kreykenbohm, G. Lamer, I. Lomakin, I. Lapshov, T. Liu, C. Maitra, N. Meidinger, B. Menz, A. Merloni, T. Mernik, B. Mican, J. Mohr, S. Müller, K. Nandra, V. Nazarov, F. Pacaud, M. Pavlinsky, E. Perinati, E. Pfeffermann, D. Pietschner, M. E. Ramos-Ceja, A. Rau, J. Reiffers, T.H. Reiprich, J. Robrade, M. Salvato, J. Sanders, A. Santangelo, M. Sasaki, H. Scheuerle, C. Schmid, J. Schmitt, A. Schwope, A. Shirshakov, M. Steinmetz, I. Stewart, L. Strüder, R. Sunyaev, C. Tenzer, L. Tiedemann, J. Trümper, V. Voron, P. Weber, J. Wilms, V. Yaroshenko, The eROSITA X-ray telescope on SRG. *Astron. Astrophys.* **647**, A1 (2021).

47. G. Prigozhin, SPIE (2012), vol. 8453 of *Society of Photo-Optical Instrumentation Engineers (SPIE) Conference Series*, p. 845318.

48. R. A. Remillard, M. Loewenstein, J. F. Steiner, G. Y. Prigozhin, B. LaMarr, T. Enoto, K. C. Gendreau, Z. Arzoumanian, C. Markwardt, A. Basak, A. L. Stevens, P. S. Ray, D. Altamirano, D. J. K. Buisson, An empirical background model for the NICER x-ray timing instrument. *Astronom. J.* 163 130 (2022).

49. J. S. Kaastra, J. A. M. Bleeker, Optimal binning of X-ray spectra and response matrix design. *Astron. Astrophys.* **587**, A151 (2016).

50. R. Sunyaev, V. Arefiev, V. Babyshkin, A. Bogomolov, K. Borisov, M. Buntov, H. Brunner, R. Burenin, E. Churazov, D. Coutinho, J. Eder, N. Eismont, M. Freyberg, M. Gilfanov, P. Gureyev, G. Hasinger, I. Khabibullin, V. Kolmykov, S. Komovkin, R. Krivonos, I. Lapshov, V. Levin, I. Lomakin, A. Lutovinov, P. Medvedev, A. Merloni, T. Mernik, E. Mikhailov, V. Molodtsov, P. Mzhelsky, S. Müller, K. Nandra, V. Nazarov, M. Pavlinsky, A. Poghodin, P. Predehl, J. Robrade, S. Sazonov, H. Scheuerle, A. Shirshakov, A. Tkachenko and V. Voron, The

SRG X-ray orbital observatory, its telescopes and first scientific results. *Astron. Astrophy.* **656**, A132 (2021).

51. H. Brunner, T. Liu, G. Lamer, A. Georgakakis, A. Merloni, M. Brusa, E. Bulbul, K. Dennerl, S. Friedrich, A. Liu, C. Maitra, K. Nandra, M. E. Ramos-Ceja, J. S. Sanders, I. M. Stewart, T. Boller, J. Buchner, N. Clerc, J. Comparat, T. Dwelly, D. Eckert, A. Finoguenov, M. Freyberg, V. Ghirardini, A. Gueguen, F. Haberl, I. Kreykenbohm, M. Krumpe, S. Osterhage, F. Pacaud, P. Predehl, T. H. Reiprich, J. Robrade, M. Salvato, A. Santangelo, T. Schrabback, A. Schwope, J. Wilms, The eROSITA final equatorial depth survey (eFEDS): The x-ray catalog. arXiv:2106.14517 [astro-ph.HE] (2021).

52. P. W. A. Roming, T. E. Kennedy, K. O. Mason, J. A. Nousek, L. Ahr, R. E. Bingham, P. S. Broos, M. J. Carter, B. K. Hancock, H. E. Huckle, S. D. Hunsberger, H. Kawakami, R. Killough, T. S. Koch, M. K. Mclelland, K. Smith, P. J. Smith, J. C. Soto, P. T. Boyd, A. A. Breeveld, S. T. Holland, M. Ivanushkina, M. S. Pryzby, M. D. Still, J. Stock, The Swift ultra-violet/optical telescope. *Space Sci. Rev.* **120**, 95– 142 (2005).

53. G. R. Ricker, Transiting exoplanet survey satellite (TESS). *J. Astron. Telesc. Instrum. Syst.* **1**, 014003 (2015).

54. I. Arcavi, StarDestroyers transient classification report for 2021-01-12. *Transient Name Server Classification Report* **2021**, 1–2460 (2021).

55. J. L. Marshall, Ground-based and airborne instrumentation for astronomy II, in *Society of Photo-Optical Instrumentation Engineers (SPIE) Conference Series*, I. S. McLean, M. M. Casali, Eds. (SPIE, 2008), vol. 7014, p. 701454.

56. R. M. Cutri, *et al.*, VizieR online data catalog: AllWISE data release (Cutri+ 2013). *VizieR Online Data Catalog* II/328 (2013).

57. T. M. C. Abbott, F. B. Abdalla, S. Allam, A. Amara, J. Annis, J. Asorey, S. Avila, O. Ballester, M. Banerji, W. Barkhouse, L. Baruah, M. Baumer, K. Bechtol, M. R. Becker, A. Benoit-Lévy, G. M. Bernstein, E. Bertin, J. Blazek, S. Bocquet, D. Brooks, D. Brout, E. Buckley-Geer, D. L. Burke, V. Busti, R. Campisano, L. Cardiel-Sas, A. C. Rosell, M. C. Kind, J. Carretero, F. J. Castander, R. Cawthon, C. Chang, X. Chen, C. Conselice, G. Costa, M. Crocce, C. E. Cunha, C. B. D'Andrea, L. N. . Costa, R. das, G. Daues, T. M. Davis, C. Davis, J. D. Vicente, D. L. DePoy, J. DeRose, S. Desai, H. T. Diehl, J. P. Dietrich, S. Dodelson, P. Doel, A. Drlica-Wagner, T. F. Eifler, A. E. Elliott, A. E. Evrard, A. Farahi, A. F. Neto, E. Fernandez, D. A. Finley, B. Flaugher, R. J. Foley, P. Fosalba, D. N. Friedel, J. Frieman, J. García-Bellido, E. Gaztanaga, D. W. Gerdes, T. Giannantonio, M. S. S. Gill, K. Glazebrook, D. A. Goldstein, M. Gower, D. Gruen, R. A. Gruendl, J. Gschwend, R. R. Gupta, G. Gutierrez, S. Hamilton, W. G. Hartley, S. R. Hinton, J. M. Hislop, D. Hollowood, K. Honscheid, B. Hoyle, D. Huterer, B. Jain, D. J. James, T. Jeltema, M. W. G. Johnson, M. D. Johnson, T. Kacprzak, S. Kent, G. Khullar, M. Klein, A. Kovacs, A. M. G. Koziol, E. Krause, A. Kremin, R. Kron, K. Kuehn, S. Kuhlmann, N. Kuropatkin, O. Lahav, J. Lasker, T. S. Li, R. T. Li, A. R. Liddle, M. Lima, H. Lin, P. López-Reyes, N. MacCrann, M. A. G. Maia, J. D. Maloney, M. Manera, M. March, J. Marriner, J. L. Marshall, P. Martini, T. McClintock, T. McKay, R. G. McMahon, P. Melchior, F. Menanteau, C. J. Miller, R. Miquel, J. J. Mohr, E. Morganson, J. Mould, E. Neilsen, R. C. Nichol, F. Nogueira, B. Nord, P. Nugent, L. Nunes, R. L. C. Ogando, L. Old, A. B. Pace, A. Palmese, F. Paz-Chinchón, H. V. Peiris, W. J. Percival, D. Petravick, A. A. Plazas, J. Poh, C. Pond, A. Porredon, A. Pujol, A. Refregier, K. Reil, P. M. Ricker, R. P. Rollins, A. K. Romer, A. Roodman, P. Rooney, A. J. Ross, E. S. Rykoff, M. Sako, M. L. Sanchez, E. Sanchez, B. Santiago, A. Saro, V. Scarpine, D. Scolnic, S. Serrano, I. Sevilla-Noarbe, E. Sheldon, N. Shipp, M. L. Silveira, M.

Smith, R. C. Smith, J. A. Smith, M. Soares-Santos, F. Sobreira, J. Song, A. Stebbins, E. Suchyta, M. Sullivan, M. E. C. Swanson, G. Tarle, J. Thaler, D. Thomas, R. C. Thomas, M. A. Troxel, D. L. Tucker, V. Vikram, A. K. Vivas, A. R. Walker, R. H. Wechsler, J. Weller, W. Wester, R. C. Wolf, H. Wu, B. Yanny, A. Zenteno, Y. Zhang, J. Zuntz, (DES Collaboration), S. Juneau, M. Fitzpatrick, R. Nikutta, D. Nidever, K. Olsen, A. Scott, (NOAO Data Lab), The dark energy survey: Data release 1. *Astrophys. J. Suppl.* **239**, 18 (2018).

58. L. Bianchi, J. Herald, B. Efremova, L. Girardi, A. Zabot, P. Marigo, A. Conti, B. Shiao, GALEX catalogs of UV sources: Statistical properties and sample science applications: Hot white dwarfs in the Milky Way. *Ap&SS* **335**, 161– 169 (2011).

59. C. Million, S. W. Fleming, B. Shiao, M. Seibert, P. Loyd, M. Tucker, M. Smith, R. Thompson, R. L. White, gPhoton: The GALEX photon data archive. *Astrophys. J.* **833**, 292 (2016).

60. T. M. Brown, N. Baliber, F. B. Bianco, M. Bowman, B. Burleson, P. Conway, M. Crellin, É. Depagne, J. de Vera, B. Dilday, D. Dragomir, M. Dubberley, J. D. Eastman, M. Elphick, M. Falarski, S. Foale, M. Ford, B. J. Fulton, J. Garza, E. L. Gomez, M. Graham, R. Greene, B. Haldeman, E. Hawkins, B. Haworth, R. Haynes, M. Hidas, A. E. Hjelstrom, D. A. Howell, J. Hygelund, T. A. Lister, R. Lobdill, J. Martinez, D. S. Mullins, M. Norbury, J. Parrent, R. Paulson, D. L. Petry, A. Pickles, V. Posner, W. E. Rosing, R. Ross, D. J. Sand, E. S. Saunders, J. Shobbrook, A. Shporer, R. A. Street, D. Thomas, Y. Tsapras, J. R. Tufts, S. Valenti, K. Vander Horst, Z. Walker, G. White, M. Willis, Las Cumbres observatory global telescope network. *Publ. Astron. Soc. Pac.* **125**, 1031– 1055 (2013).

61. M. M. Fausnaugh, P. J. Vallely, C. S. Kochanek, B. J. Shappee, K. Z. Stanek, M. A. Tucker, G. R. Ricker, R. Vanderspek, D. W. Latham, S. Seager, J. N. Winn, J. M. Jenkins, Z. K. Berta-

Thompson, T. Daylan, J. P. Doty, G. Fűrész, A. M. Levine, R. Morris, A. Pál, L. Sha, E. B. Ting, B. Wohler, Early-time light curves of type Ia supernovae observed with TESS. *Astrophys. J.* **908**, 51 (2021).

62. C. Alard, R. H. Lupton, A method for optimal image subtraction. *Astrophys. J.* **503**, 325 (1998), 331.

63. C. Alard, Image subtraction using a space-varying kernel. *Astron. Astrophys. Suppl.* **144**, 363– 370 (2000).

64. P. J. Vallely, M. Fausnaugh, S. W. Jha, M. A. Tucker, Y. Eweis, B. J. Shappee, C. S. Kochanek, K. Z. Stanek, P. Chen, S. Dong, J. L. Prieto, T. Sukhbold, T. A. Thompson, J. Brimacombe, M. D. Stritzinger, T. W.S. Holoien, D. A. H. Buckley, M. Gromadzki, S. Bose, ASASSN-18tb: A most unusual type Ia supernova observed by TESS and SALT. *Mon. Not. R. Astron. Soc.* **487**, 2372– 2384 (2019).

65. T. W. S. Holoien, P. J. Vallely, K. Auchettl, K. Z. Stanek, C. S. Kochanek, K. D. French, J. L. Prieto, B. J. Shappee, J. S. Brown, M. M. Fausnaugh, S. Dong, T. A. Thompson, S. Bose, J. M. M. Neustadt, P. Cacella, J. Brimacombe, M. R. Kendurkar, R. L. Beaton, K. Boutsia, L. Chomiuk, T. Connor, N. Morrell, A. B. Newman, G. C. Rudie, L. Shishkovksy, J. Strader, Discovery and early evolution of ASASSN-19bt, the first TDE detected by TESS. *Astrophys. J.* **883**–111 (2019).

66. A. G. Riess, The rise time of nearby type IA supernovae. *Astron. J.* **118**, 2675 (1999).

67. J. T. Hinkle, Discovery and follow-up of ASASSN-19dj: An X-ray and UV luminous TDE in an extreme post-starburst galaxy. *Mon. Not. R. Astron. Soc.* **500**, 1673 (2021).

68. M. Nicholl, T. Wevers, S. R. Oates, K. D. Alexander, G. Leloudas, F. Onori, A. Jerkstrand, S. Gomez, S. Campana, I. Arcavi, P. Charalampopoulos, M. Gromadzki, N. Ihanec, P. G. Jonker,

A. Lawrence, I. Mandel, S. Schulze, P. Short, J. Burke, C. McCully, D. Hiramatsu, D. A. Howell, C. Pellegrino, H. Abbot, J. P. Anderson, E. Berger, P. K. Blanchard, G. Cannizzaro, T.W. Chen, M. Dennefeld, L. Galbany, S. González-Gaitán, G. Hosseinzadeh, C. Inserra, I. Irani, P. Kuin, T. Müller-Bravo, J. Pineda, N. P. Ross, R. Roy, S. J. Smartt, K. W. Smith, B. Tucker, Ł. Wyrzykowski, D. R. Young, An outflow powers the optical rise of the nearby, fast-evolving tidal disruption event AT2019qiz. *Mon. Not. R. Astron. Soc.* **499**, 482–504 (2020).

69. A. V. Payne, K. Auchettl, B. J. Shappee, C. S. Kochanek, P. T. Boyd, T. W.-S. Holoien, M. M. Fausnaugh, C. Ashall, J. T. Hinkle, P. J. Vallely, K. Z. Stanek, T. A. Thompson, Chandra, HST/STIS, NICER, Swift, and TESS detail the flare evolution of the repeating nuclear transient ASASSN-14ko. *Astrophys. J.* **951**, 134 (2023).

70. J. T. Hinkle, T. W.S. Holoien, B. J. Shappee, J. M. M. Neustadt, K. Auchettl, P. J. Vallely, M. Shahbandeh, M. Kluge, C. S. Kochanek, K. Z. Stanek, M. E. Huber, R. S. Post, D. Bersier, C. Ashall, M. A. Tucker, J. P. Williams, T. de Jaeger, A. do, M. Fausnaugh, D. Gruen, U. Hopp, J. Myles, C. Obermeier, A. V. Payne, T. A. Thompson, The curious case of ASASSN-20hx: A slowly evolving, UV- and X-ray-luminous, ambiguous nuclear transient, *ambiguous nuclear transient. Astrophys. J.* **930**, 12 (2022).

71. S. Valenti, D. Sand, A. Pastorello, M. L. Graham, D. A. Howell, J. T. Parrent, L. Tomasella, P. Ochner, M. Fraser, S. Benetti, F. Yuan, S. J. Smartt, J. R. Maund, I. Arcavi, A. Gal-Yam, C. Inserra, D. Young, The first month of evolution of the slow-rising type IIP SN 2013ej in M74*. *Mon. Not. R. Astron. Soc.* **438**, L101– L105 (2014).

72. D. D. Kelson, G. D. Illingworth, P. G. van Dokkum, M. Franx, The evolution of early-type galaxies in distant clusters. II. Internal kinematics of 55 galaxies in the *z*=0.33 cluster CL 1358+62. *Astrophys. J.* **531**, 159– 183 (2000).

73. D. D. Kelson, Optimal techniques in two-dimensional spectroscopy: Background subtraction for the 21st century. *Publ. Astron. Soc. Pac.* **115**, 688– 699 (2003).

74. D. McConnell, The rapid ASKAP continuum survey I: Design and first results. *Publ. Astron. Soc. Aus.* **37**, e048 (2020).

75. C. L. Hale, D. McConnell, A. J. M. Thomson, E. Lenc, G. H. Heald, A. W. Hotan, J. K. Leung, V. A. Moss, T. Murphy, J. Pritchard, E. M. Sadler, A. J. Stewart, M. T. Whiting, The rapid ASKAP continuum survey paper II: First stokes I Source catalogue data release. *Publ. Astron. Soc. Aus.* **38**, e058 (2021).

76. T. Murphy, VAST: An ASKAP survey for variables and slow transients. *Publ. Astron. Soc. Aus.* **30**, e006 (2013).

77. T. Murphy, D. L. Kaplan, A. J. Stewart, A. O'Brien, E. Lenc, S. Pintaldi, J. Pritchard, D. Dobie, A. Fox, J. K. Leung, T. An, M. E. Bell, J. W. Broderick, S. Chatterjee, S. Dai, D. d'Antonio, G. Doyle, B. M. Gaensler, G. Heald, A. Horesh, M. L. Jones, D. McConnell, V. A. Moss, W. Raja, G. Ramsay, S. Ryder, E. M. Sadler, G. R. Sivakoff, Y. Wang, Z. Wang, M. S. Wheatland, M. Whiting, J. R. Allison, C. S. Anderson, L. Ball, K. Bannister, D. C.J. Bock, R. Bolton, J. D. Bunton, R. Chekkala, A. P. Chippendale, F. R. Cooray, N. Gupta, D. B. Hayman, K. Jeganathan, B. Koribalski, K. Lee-Waddell, E. K. Mahony, J. Marvil, N. M. McClure-Griffiths, P. Mirtschin, A. Ng, S. Pearce, C. Phillips, M. A. Voronkov, The ASKAP variables and slow transients (VAST) pilot survey. *Publ. Astron. Soc. Aus.* **38**, e054 (2021).

78. S. Pintaldi, A. Stewart, A. O'Brien, D. Kaplan, T. Murphy, A scalable transient detection pipeline for the Australian SKA Pathfinder VAST survey. arXiv:2101.05898 [astro-ph.IM] (2021).

79. H. Guo, Y. Shen, S. Wang, PyQSOFit: Python code to fit the spectrum of quasars, Astrophysics Source Code Library (2018).

80. S. Rakshit, C. S. Stalin, J. Kotilainen, Spectral properties of quasars from Sloan Digital Sky Survey Data Release 14: The catalog. *Astrophys. J. Suppl.* **249**, 17 (2020).

81. J. A. Cardelli, G. C. Clayton, J. S. Mathis, The relationship between infrared, optical, and ultraviolet extinction. *Astrophys. J.* **345**, 245 (1989).

82. D. J. Schlegel, D. P. Finkbeiner, M. Davis, Maps of dust infrared emission for use in estimation of reddening and cosmic microwave background radiation foregrounds. *Astrophys. J.* **500**, 525– 553 (1998).

83. T. A. Boroson, R. F. Green, The emission-line properties of low-redshift quasi-stellar objects. *Astrophys. J. Suppl.* **80**, 109 (1992).

84. S. Rakshit, C. S. Stalin, H. Chand, X.-G. Zhang, A catalog of narrow line Seyfert 1 galaxies from the Sloan Digital Sky Survey Data Release 12. *Astrophys. J. Suppl.* **229**, 39 (2017).

85. J.-H. Woo, Y. Yoon, S. Park, D. Park, S. C. Kim, The Black hole mass-stellar velocity dispersion relation of narrow-line Seyfert 1 galaxies. *Astrophys. J.* **801**, 38 (2015).

86. Y. Shen, The mass of quasars. *Bull. Astron. Soc. India* **41**, 61 (2013).

87. J. A. Baldwin, M. M. Phillips, R. Terlevich, Classification parameters for the emission-line spectra of extragalactic objects. *Publ. Astron. Soc. Pac.* **93**, 5 (1981).

88. R. Cid Fernandes, G. Stasinska, A. Mateus, N. Vale Asari, A comprehensive classification of galaxies in the Sloan Digital Sky Survey: How to tell true from fake AGN?. *Not. R. Astron. Soc.* **413**, 1687– 1699 (2011).

89. L. J. Kewley, M. A. Dopita, R. S. Sutherland, C. A. Heisler, J. Trevena, Theoretical modeling of starburst galaxies. *Astrophys. J.* **556**, 121– 140 (2001).

90. G. Kauffmann, T. M. Heckman, C. Tremonti, J. Brinchmann, S. Charlot, S. D. M. White, S. E. Ridgway, J. Brinkmann, M. Fukugita, P. B. Hall, Ž. Ivezić, G. T. Richards, D. P. Schneider, The host galaxies of active galactic nuclei. *Mon. Not. R. Astron. Soc.* **346**, 1055– 1077 (2003).

91. K. Schawinski, D. Thomas, M. Sarzi, C. Maraston, S. Kaviraj, S.J. Joo, S. K. Yi, J. Silk, Observational evidence for AGN feedback in early-type galaxies. *Mon. Not. R. Astron. Soc.* **382**, 1415– 1431 (2007).

92. C. Conroy, J. E. Gunn, M. White, The propagation of uncertainties in stellar population synthesis modeling. I. The relevance of uncertain aspects of stellar evolution and the initial mass function to the derived physical properties of galaxies. *Astrophys. J.* **699**, 486– 506 (2009).

93. B. D. Johnson, J. Leja, C. Conroy, J. S. Speagle, Stellar population inference with prospector. *Astrophys. J. Suppl.* **254**, 22 (2021).

94. D. Foreman-Mackey, D. W. Hogg, D. Lang, J. Goodman, emcee: The MCMC hammer. *Publ. Astron. Soc. Pac.* **125**, 306– 312 (2013).

95. D. Calzetti, L. Armus, R. C. Bohlin, A. L. Kinney, J. Koornneef, T. Storchi-Bergmann, The dust content and opacity of actively star-forming galaxies. *Astrophys. J.* **533**, 682 (2000), 695.

96. E. F. Schlafly, D. P. Finkbeiner, Measuring reddening with Sloan Digital Sky Survey stellar spectra and recalibrating SFD. *Astrophys. J.* **737**, 103 (2011).

97. J. E. Greene, J. Strader, L. C. Ho, Intermediate-mass black holes. *Annu. Rev. Astron. Astrophys.* **58**, 257 (2020).

98. E. Nardini, J. N. Reeves, J. Gofford, F. A. Harrison, G. Risaliti, V. Braito, M. T. Costa, G. A. Matzeu, D. J. Walton, E. Behar, S. E. Boggs, F. E. Christensen, W. W. Craig, C. J. Hailey, G. Matt, J. M. Miller, P. T. O'Brien, D. Stern, T. J. Turner, M. J. Ward, Black hole feedback in the luminous quasar PDS 456. *Science* **347**, 860– 863 (2015).

99. K. A. Arnaud, Astronomical data analysis software and systems V, in *Astronomical Society of the Pacific Conference Series*, G. H. Jacoby, J. Barnes, Eds. (1996), vol. 101, p. 17.

100. HI4PI Collaboration, HI4PI: A full-sky H I survey based on EBHIS and GASS. *Astron. Astrophys.* **594**, A116 (2016).

101. M. Dadina, Seyfert galaxies in the local Universe (z ≤0.1): The average X-ray spectrum as seen byBeppoSAX. *Astron. Astrophys.* **485**, 417– 424 (2008).

102. E. Kara, L. Dai, C. S. Reynolds, T. Kallman, Ultrafast outflow in tidal disruption event ASASSN-14li. *Mon. Not. R. Astron. Soc.* **474**, 3593– 3598 (2018).

103. M. Laurenti, A. Luminari, F. Tombesi, F. Vagnetti, R. Middei, E. Piconcelli, Location and energetics of the ultra-fast outflow in PG 1448+273. *Astron. Astrophys.* **645**, A118 (2021).

104. T. Kallman, M. Bautista, Photoionization and high-density gas. *Astrophys. J. Suppl.* **133**, 221– 253 (2001).

105. C. Ricci, B. Trakhtenbrot, M. J. Koss, Y. Ueda, K. Schawinski, K. Oh, I. Lamperti, R. Mushotzky, E. Treister, L. C. Ho, A. Weigel, F. E. Bauer, S. Paltani, A. C. Fabian, Y. Xie, N. Gehrels, The close environments of accreting massive black holes are shaped by radiative feedback. *Nature* **549**, 488– 491 (2017).

106. D. M. Crenshaw, S. B. Kraemer, Feedback from mass outflows in nearby active galactic nuclei. I. Ultraviolet and X-ray absorbers. *Astrophys. J.* **753**, 75 (2012).

107. F. Tombesi, M. Cappi, J. N. Reeves, R. S. Nemmen, V. Braito, M. Gaspari, C. S. Reynolds, Unification of X-ray winds in Seyfert galaxies: From ultra-fast outflows to warm absorbers. *Mon. Not. R. Astron. Soc.* **430**, 1102– 1117 (2013).

108. J. Gofford, J.N. Reeves, D.E. McLaughlin, V. Braito, T.J. Turner, F. Tombesi, M. Cappi, The Suzaku view of highly ionized outflows in AGN—II. Location, energetics and scalings with bolometric luminosity. *Mon. Not. R. Astron. Soc.* **451**, 4169– 4182 (2015).

109. P. Kosec, D. Pasham, E. Kara, F. Tombesi, Discovery of a variable multiphase outflow in the x-ray-emitting tidal disruption event ASASSN-20qc. *Astrophys. J.* **954**, 170 (2023).

110. R. G. Detmers, J. S. Kaastra, E. Costantini, F. Verbunt, M. Cappi, C. de Vries, The outflow in Mrk 509. *Astron. Astrophys.* **516**, A61 (2010).

111. G. Miniutti, R. D. Saxton, M. Giustini, K. D. Alexander, R. P. Fender, I. Heywood, I. Monageng, M. Coriat, A. K. Tzioumis, A. M. Read, C. Knigge, P. Gandhi, M. L. Pretorius, B. Agís-González, Nine-hour X-ray quasi-periodic eruptions from a low-mass black hole galactic nucleus. *Nature* **573**, 381– 384 (2019).

112. M. Giustini, G. Miniutti, R. D. Saxton, X-ray quasi-periodic eruptions from the galactic nucleus of RX J1301.9+2747. *Astron. Astrophys.* **636**, L2 (2020).

113. J. Garcia, Improved reflection models of black hole accretion disks: Treating the angular distribution of x-rays. *Astrophys. J.* **782**, 76 (2014).

114. C. Ricci, M. Loewenstein, E. Kara, R. Remillard, B. Trakhtenbrot, I. Arcavi, K. C. Gendreau, Z. Arzoumanian, A. C. Fabian, R. Li, L. C. Ho, C. L. MacLeod, E. Cackett, D. Altamirano, P. Gandhi, P. Kosec, D. Pasham, J. Steiner, C.H. Chan, The 450 day x-ray monitoring of the changing-look AGN 1ES 1927+654. *Astrophys. J. Suppl.* **255**, 7 (2021).

115. E. Agol, J. H. Krolik, Magnetic stress at the marginally stable orbit: Altered disk structure, radiation, and black hole spin evolution. *Astrophys. J.* **528**, 161– 170 (2000).

116. S. Gezari, Tidal disruption events. *Annu. Rev. Astron. Astrophys.* **59**, 21 (2021).

117. R. S. Nemmen, T. Storchi-Bergmann, M. Eracleous, Spectral models for low-luminosity active galactic nuclei in LINERs: The role of advection-dominated accretion and jets. *Mon. Not. R. Astron. Soc.* **438**, 2804– 2827 (2014).

118. A. Mummery, S. A. Balbus, The spectral evolution of disc dominated tidal disruption events. *Mon. Not. R. Astron. Soc.* **492**, 5655– 5674 (2020).

119. A. Mummery, A maximum X-ray luminosity scale of disc-dominated tidal destruction events. *Mon. Not. R. Astron. Soc.* **504**, 5144– 5154 (2021).

120. G. Foster, Wavelets for period analysis of unevenly sampled time series. *Astron. J.* **112**, 1709 (1996).

121. Pyleoclim: A python package for the analysis of paleoclimate data (2019).

122. D. R. Pasham, Evidence for a compact object in the aftermath of the extragalactic transient AT2018cow. *Nat. Astron.* **6**, 249 (2022).

123. J. Timmer, M. Koenig, On generating power law noise. *Astron. Astrophys.* **300**, 707 (1995).

124. F. Tombesi, M. Cappi, J. N. Reeves, G. G. C. Palumbo, V. Braito, M. Dadina, Evidence for ultra-fast outflows in radio-quiet active galactic nuclei. II. Detailed photoionization modeling of Fe K-shell absorption lines. *Astrophys. J.* **742**, 44 (2011).

125. K. Fukumura, F. Tombesi, Constraining X-ray coronal size with transverse motion of AGN ultra-fast outflows. *Astrophys. J.* **885**, L38 (2019).

126. S. Laha, M. Guainazzi, G. C. Dewangan, S. Chakravorty, A. K. Kembhavi, Warm absorbers in X-rays (WAX), a comprehensive high-resolution grating spectral study of a sample of Seyfert galaxies—I. A global view and frequency of occurrence of warm absorbers. *Mon. Not. R. Astron. Soc.* **441**, 2613– 2643 (2014).

127. T. W. S. Holoien, J. M. M. Neustadt, P. J. Vallely, K. Auchettl, J. T. Hinkle, C. Romero-Cañizales, B. J. Shappee, C. S. Kochanek, K. Z. Stanek, P. Chen, S. Dong, J. L. Prieto, T. A. Thompson, T. G. Brink, A. V. Filippenko, W.K. Zheng, D. Bersier, S. Bose, A. J. Burgasser, S. Channa, T. de Jaeger, J. Hestenes, M. Im, B. Jeffers, H. D. Jun, G. Lansbury, R. S. Post, T. W. Ross, D. Stern, K. Tang, M. A. Tucker, S. Valenti, S. Yunus, K. D. Zhang, Investigating the nature of the luminous ambiguous nuclear transient ASASSN-17jz. *Astrophys. J.* **933**, 196 (2022).

128. G. Chartas, M. Cappi, C. Vignali, M. Dadina, V. James, G. Lanzuisi, M. Giustini, M. Gaspari, S. Strickland, E. Bertola, Multiphase powerful outflows detected in high-z quasars. *Astrophys. J.* **920**, 24 (2021).

129. T. Di Matteo, V. Springel, L. Hernquist, Energy input from quasars regulates the growth and activity of black holes and their host galaxies. *Nature* **433**, 604– 607 (2005).

130. R. F. Stellingwerf, Period determination using phase dispersion minimization. *Astrophys. J.* **224**, 953 (1978).

131. N. Stone, A. Loeb, Observing lense-thirring precession in tidal disruption flares. *Phys. Rev. Lett.* **108**, 061302 (2012).

132. R. Arcodia, A. Merloni, K. Nandra, J. Buchner, M. Salvato, D. Pasham, R. Remillard, J. Comparat, G. Lamer, G. Ponti, A. Malyali, J. Wolf, Z. Arzoumanian, D. Bogensberger, D. A. H. Buckley, K. Gendreau, M. Gromadzki, E. Kara, M. Krumpe, C. Markwardt, M. E. Ramos-Ceja, A. Rau, M. Schramm, A. Schwope, X-ray quasi-periodic eruptions from two previously quiescent galaxies. *Nature* **592**, 704– 707 (2021).

133. G. Miniutti, Disappearance of quasi-periodic-eruptions (QPEs) in GSN 069, simultaneous X-ray re-brightening, and predicted QPE re-appearance. arXiv:2207.07511 [astro-ph.HE] (2022).

134. J. Dexter, A. Tchekhovskoy, A. Jiménez-Rosales, S. M. Ressler, M. Bauböck, Y. Dallilar, P. T. de Zeeuw, F. Eisenhauer, S. von Fellenberg, F. Gao, R. Genzel, S. Gillessen, M. Habibi, T. Ott, J. Stadler, O. Straub, F. Widmann, Sgr A* near-infrared flares from reconnection events in a magnetically arrested disc. *Mon. Not. R. Astron. Soc.* **497**, 4999– 5007 (2020).

135. N. Neumayer, A. Seth, T. Boker, Nuclear star *clusters. Astrophys. Rev.* **28**, 4 (2020).

136. R. Schodel, The Milky Way's nuclear star cluster: Old, metal-rich, and cuspy. Structure and star formation history from deep imaging. *Astron. Astrophys.* **641**, A102 (2020).

137. M. Zajacek, B. Czerny, R. Schodel, N. Werner, V. Karas, Black-hole activity feedback across vast *scales Astronomy* **6**, 1008– 1010 (2022).

138. F. Peißker, A. Eckart, M. Zajacek, B. Ali, M. Parsa, S62 and S4711: Indications of a population of faint fast-moving stars inside the S2 orbit—S4711 on a 7.6 yr orbit around Sgr A*. *Astrophys. J.* **899**, 50 (2020).

139. F. Peißker, A. Eckart, M. Zajacek, S. Britzen, Observation of S4716—A star with a 4 yr orbit around Sgr A*. *Astrophys. J.* **933**, 49 (2022).

140. S. F. Portegies Zwart, S. L. W. McMillan, The runaway growth of intermediate-mass black holes in dense star clusters. *Astrophys. J.* **576**, 899– 907 (2002).

141. F. A. Rasio, M. Freitag, M. A. Gurkan, in *Coevolution of Black Holes and Galaxies*, L. C. Ho, Ed. (2004), p. 138.

142. S. C. Rose, S. Naoz, R. Sari, I. Linial, The formation of intermediate-mass black holes in galactic nuclei. *Astrophys. J.* **929**, L22 (2022).

143. G. Fragione, B. Kocsis, F. A. Rasio, J. Silk, Repeated mergers, mass-gap black holes, and formation of intermediate-mass black holes in dense massive star clusters. *Astrophys. J.* **927**, 231 (2022).

144. H. Netzer, Revisiting the unified model of active galactic nuclei. *Annu. Rev. Astron. Astrophys.* **53**, 365 (2015).

145. I. Linial, B. D. Metzger, EMRI + TDE = QPE: Periodic x-ray flares from star-disk collisions in galactic nuclei. *Astrophys. J.* **957**, 34 (2023).

146. A. Franchini, M. Bonetti, A. Lupi, G. Miniutti, E. Bortolas, M. Giustini, M. Dotti, A. Sesana, R. Arcodia, T. Ryu, Quasi-periodic eruptions from impacts between the secondary and a rigidly precessing accretion disc in an extreme mass-ratio inspiral system. *Astron. Astrophys.* **675**, A100 (2023).

147. B. Czerny, A. Rozanska, J. Kuraszkiewicz, Constraints for the accretion disk evaporation´ rate in AGN from the existence of the Broad Line Region. *Astron. Astrophys.* **428**, 39– 49 (2004).

148. M. A. Abramowicz, X. Chen, S. Kato, J.-P. Lasota, O. Regev, Thermal equilibria of accretion disks. *Astrophys. J.* **438**, L37 (1995).

149. F. Honma, Global structure of bimodal accretion disks around a black hole. *Publ. Astron. Soc. Japan* **48**, 77– 87 (1996).

150. S. Kato, K. E. Nakamura, Transition radius from cooling-dominated to advection-dominated regimes in two temperature disks. *Publ. Astron. Soc. Japan* **50**, 559– 566 (1998).

151. Witzany, V., Jefremov, P., New closed analytical solutions for geometrically thick fluid tori around black holes. *Astron. Astrophy.* **614**, A75 (2018).

152. W. Schmidt, Celestial mechanics in Kerr spacetime. *Class. Quant. Grav.* **19**, 2743– 2764 (2002).

153. F. Hoyle, R. A. Lyttleton, The evolution of the stars. *Proc. Cambr. Philosop. Soc.* **35**, 592– 609 (1939).

154. H. Bondi, F. Hoyle, On the mechanism of accretion by stars. *Mon. Not. R. Astron. Soc.* **104**, 273– 282 (1944).

155. E. C. Ostriker, Dynamical friction in a gaseous medium. *Astrophys. J.* **513**, 252– 258 (1999).

156. R. Narayan, Hydrodynamic drag on a compact star orbiting a supermassive black hole. *Astrophys. J.* **536**, 663– 667 (2000).

157. P. C. Peters, Gravitational radiation and the motion of two point masses. *Phys. Rev.* **136**, 1224 (1964).

158. T. Robson, N. J. Cornish, C. Liu, The construction and use of lisa sensitivity curves. *Class. Quant. Grav.* **36**, 105011 (2019).

159. J. G. Hills, Hyper-velocity and tidal stars from binaries disrupted by a massive Galactic black hole. *Nature* **331**, 687– 689 (1988).

160. M. J. Graham, K. E. S. Ford, B. McKernan, N. P. Ross, D. Stern, K. Burdge, M. Coughlin, S. G. Djorgovski, A. J. Drake, D. Duev, M. Kasliwal, A. A. Mahabal, S. van Velzen, J. Belecki, E. C. Bellm, R. Burruss, S. B. Cenko, V. Cunningham, G. Helou, S. R. Kulkarni, F. J. Masci, T. Prince, D. Reiley, H. Rodriguez, B. Rusholme, R. M. Smith, M. T. Soumagnac, Candidate electromagnetic counterpart to the binary black hole merger gravitational-wave event S190521g∗. *Phys. Rev. Lett.* **124**, 251102 (2020).

161. P. Madau, M. J. Rees, Massive black holes as population III remnants. *Astrophys. J.* **551**, L27– L30 (2001).

162. A. Ferrara, S. Salvadori, B. Yue, D. Schleicher, Initial mass function of intermediate-mass black hole seeds. *Mon. Not. R. Astron. Soc.* **443**, 2410– 2425 (2014).

163. J. Spitzer, Equipartition and the formation of compact nuclei in spherical stellar systems. *Astrophys. J.* **158**, L139 (1969).

164. M. Arca-Sedda, R. Capuzzo-Dolcetta, The MEGaN project II. Gravitational waves from intermediate-mass and binary black holes around a supermassive black hole. *Mon. Not. R. Astron. Soc.* **483**, 152– 171 (2019).

165. J. M. Bellovary, M.-M. Mac Low, B. McKernan, K. E. S. Ford, Migration traps in disks around supermassive black holes. *Astrophys. J.* **819**, L17 (2016).

166. J. D. Schnittman, A. Buonanno, The distribution of recoil velocities from merging black holes. *Astrophys. J.* **662**, L63– L66 (2007).

167. V. Varma, S. Biscoveanu, T. Islam, F. H. Shaik, C.J. Haster, M. Isi, W. M. Farr, S. E. Field, S. Vitale, Evidence of large recoil velocity from a black hole merger signal. *Phys. Rev. Lett.* **128**, 191102 (2022).

168. S. F. Honig, Redefining the Torus: A unifying view of AGNs in the infrared and submillimeter. *Astrophys. J.* **884**, 171 (2019).

169. L. Subr, V. Karas, On highly eccentric stellar trajectories interacting with a self-gravitating disc in Sgr *A*. Astron. Astrophys.* **433**, 405– 413 (2005).

170. D. Syer, C. J. Clarke, M. J. Rees, Star-disc interactions near a massive black hole. *Mon. Not. R. Astron. Soc.* **250**, 505– 512 (1991).

171. N. I. Shakura, R. A. Sunyaev, Black holes in binary systems. Observational appearance. *Astron. Astrophys.* **24**, 337 (1973).

172. D. Vokrouhlicky, V. Karas, Stellar capture by an accretion disc. *Mon. Not. R. Astron. Soc.* **293**, L1– L5 (1998).

173. L. Subr, V. Karas, An orbiter crossing an accretion disc. *Astron. Astrophys.* **352**, 452 (1999).

174. W. H. Zurek, A. Siemiginowska, S. A. Colgate, Star-disk collisions and the origin of the broad lines in quasars. *Astrophys. J.* **434**, 46 (1994).

175. E. Y. Vilkoviskij, B. Czerny, The role of the central stellar cluster in active galactic nucleiI. Semi-analytical model. *Astron. Astrophys.* **387**, 804– 817 (2002).

176. D. Vokrouhlicky, V. Karas, Stellar dynamics in a galactic centre surrounded by a massive accretion disc—I. Newtonian description. *Mon. Not. R. Astron. Soc.* **298**, 53– 66 (1998).

177. T. Liu, X.-Q. Xu, X.-H. Liao, Orbital dynamics with the gravitational perturbation due to a disk. *Astrophys. J.* **901**, 170 (2020).

178. V. Karas, L. Subr, Orbital decay of satellites crossing an accretion disc *Astron. Astrophys.* **376**, 686– 696 (2001).

179. L. J. Dai, S. V. Fuerst, R. Blandford, Quasi-periodic flares from star-accretion-disc collisions. *Mon. Not. R. Astron. Soc.* **402**, 1614 (2010).

180. W. H. Zurek, A. Siemiginowska, S. A. Colgate, Star-disk collisions and the origin of the broad lines in quasars: Addendum. *Astrophys. J.* **470**, 652 (1996).

181. P. B. Ivanov, I. V. Igumenshchev, I. D. Novikov, Hydrodynamics of black hole-accretion disk collision. *Astrophys. J.* **507**, 131– 144 (1998).

182. M. T. P. Liska, G. Musoke, A. Tchekhovskoy, O. Porth, A. M. Beloborodov, Formation of magnetically truncated accretion disks in 3D radiation-transport two-temperature GRMHD simulations. *Astrophys. J.* **935**, L1 (2022).

183. B. Curd, R. Narayan, GRRMHD simulations of MAD accretion discs declining from super-Eddington to sub-Eddington accretion rates. *Mon. Not. R. Astron. Soc.* **518**, 3441 (2023).

184. F. Meyer, B. F. Liu, E. Meyer-Hofmeister, Re-condensation from an ADAF into an inner disk: The intermediate state of black hole accretion? *Astron. Astrophys.* **463**, 1– 9 (2007).

185. M. Gu, X. Cao, The anticorrelation between the hard X-ray photon index and the Eddington ratio in low-luminosity active galactic nuclei. *Mon. Not. R. Astron. Soc.* **399**, 349– 356 (2009).

186. S. Panda, M. Sniegowska, Changing-Look AGNs—I. Tracking the transition on the main´ sequence of quasars. arXiv:2206.10056 [astro-ph.HE] (2022).
